# Comparative genomic and transcriptomic analysis revealed genetic characteristics related to solvent formation and xylose utilization in *Clostridium acetobutylicum *EA 2018

**DOI:** 10.1186/1471-2164-12-93

**Published:** 2011-02-02

**Authors:** Shiyuan Hu, Huajun Zheng, Yang Gu, Jingbo Zhao, Weiwen Zhang, Yunliu Yang, Shengyue Wang, Guoping Zhao, Sheng Yang, Weihong Jiang

**Affiliations:** 1 Key Laboratory of Synthetic Biology, Institute of Plant Physiology and Ecology, Shanghai Institutes for Biological Sciences, Chinese Academy of Sciences, Shanghai 200032, China; 2 Research Center of Industrial Biotechnology, Shanghai Institutes for Biological Sciences, Chinese Academy of Sciences, Shanghai 200032, China; 3 Shanghai-MOST Key Laboratory of Health and Disease Genomics, Chinese National Human Genome Center at Shanghai, Shanghai, 200032, China; 4 Center for Ecogenomics, Biodesign Institute, Arizona State University, Tempe, Arizona 85287-6501, USA

## Abstract

**Background:**

*Clostridium acetobutylicum*, a gram-positive and spore-forming anaerobe, is a major strain for the fermentative production of acetone, butanol and ethanol. But a previously isolated hyper-butanol producing strain *C. acetobutylicum *EA 2018 does not produce spores and has greater capability of solvent production, especially for butanol, than the type strain *C. acetobutylicum *ATCC 824.

**Results:**

Complete genome of *C. acetobutylicum *EA 2018 was sequenced using Roche 454 pyrosequencing. Genomic comparison with ATCC 824 identified many variations which may contribute to the hyper-butanol producing characteristics in the EA 2018 strain, including a total of 46 deletion sites and 26 insertion sites. In addition, transcriptomic profiling of gene expression in EA 2018 relative to that of ATCC824 revealed expression-level changes of several key genes related to solvent formation. For example, *spo0A *and *adhEII *have higher expression level, and most of the acid formation related genes have lower expression level in EA 2018. Interestingly, the results also showed that the variation in CEA_G2622 (CAC2613 in ATCC 824), a putative transcriptional regulator involved in xylose utilization, might accelerate utilization of substrate xylose.

**Conclusions:**

Comparative analysis of *C. acetobutylicum *hyper-butanol producing strain EA 2018 and type strain ATCC 824 at both genomic and transcriptomic levels, for the first time, provides molecular-level understanding of non-sporulation, higher solvent production and enhanced xylose utilization in the mutant EA 2018. The information could be valuable for further genetic modification of *C. acetobutylicum *for more effective butanol production.

## Background

High oil prices, growing concerns over national security and climate change are driving investment and innovation in the renewable alternative fuels [1,2]. Among various potentially alternatives, butanol has been proposed as an excellent substitute or supplement for gasoline, and has been demonstrated to work in some vehicles designed for use with gasoline without any engine modification [1]. In addition to manufacture from petroleum through chemical refinery process, industry production of butanol is typically through a so-called ABE fermentation process employing gram-positive, spore forming and anaerobic organism *Clostridium acetobutylicum *[2]. *C. acetobutylicum *is capable of producing a mixture of acetone (A), butanol (B) and ethanol (E) from a variety of carbohydrate substrates such as starch [3]. According to an estimate in 2008, butanol accounted for a 7-8.4 billon US dollar market worldwide and has a projected market expansion of 3% per year in the near future [4].

Significant efforts have been spent on physiological and genetic characterization of solvent-producing *C. acetobutylicum *in the past decades [5-8], and tools for genetic manipulation of *C. acetobutylicum *were also developed [9-11]. In 2001, the whole genome of well studied *C. acetobutylicum *ATCC 824 was sequenced, revealing a 3.94 Mb chromosome which encodes 3740 open reading frames (ORF), and a 192 Kb megaplasmid which encodes 178 ORFs [12]. Afterwards, a series of studies employing global approaches have been performed [13-16], and the genome-scale metabolic model of *C. acetobutylicum *was also constructed [17-19]. These efforts have improved the understanding of regulatory and metabolic networks of this industry significant species.

However, most of the *C. acetobutylicum *strains are not optimized systems for butanol production because their spore-forming life cycle decreases the efficiency of industrial fermentation, and the ABE fermentation process also creates a number of by-products, such as H_2_, acetic, lactic and propionic acids, acetone, isopropanol and ethanol [20]. As a result, the butanol yield is difficult to control and a significant amount of energy is wasted in these by-products. Moreover, it also increases the cost of downstream butanol purification. To address these issues, various modification approaches, such as mutagenesis by chemical or radiation agents, and genetic engineering, have been performed to improve the butanol production [10,21]. Our laboratory has previously obtained a high butanol producing strain, *C. acetobutylicum *EA 2018, through butanol resistance screening of *N*-methyl-*N*-nitro-*N*-nitrosoguanidine (NTG) treated *Clostridium *strain isolated from soil [22]. Preliminary results in a 100-ton continuous fermenter showed that butanol ratio and starch conversion rates of EA 2018 strain were 10% and 5% higher than those reported in recent literature [23]. To explore the genetic difference between EA 2018 and ATCC 824, in this study, the *C. acetobutylicum *EA 2018 genome was sequenced using Roche 454 pyrosequencing together with traditional Sanger sequencing. In addition, comparative genomic and transcriptomic analyses of EA 2018 and ATCC 824 were also performed. The study, for the first time, provides a molecular-level understanding of higher solvent production, enhanced xylose utilization and non-sporulation in the mutant EA 2018. The information could be valuable for further genetic modification of *C. acetobutylicum *for more effective butanol production.

## Results and Discussion

### Characterization of isolate EA 2018

The original solvent producing strain was isolated by our laboratory previously [22]. After several rounds of mutagenesis using NTG (*N*-methyl-*N'*-nitro-*N*-nitrosoguanidine), we obtained a hyper butanol-producing strain designated as EA 2018. This strain was later identified as *Clostridium acetobutylicum *by the China Center for Type Culture Collection (CCTCC) and was kept in CCTCC under the preservation No. CCTCC M_94061. In this work, the 16S rDNA of *C. acetobutylicum *EA 2018 was cloned and sequenced. The 1399 bp 16S rDNA sequence of *C. acetobutylicum *EA 2018 was 100% identical to that of the type strain *C. acetobutylicum *ATCC 824 (Accession number NC_003030 for ATCC 824 genome sequence) [12]. Furthermore, the *sol *operon involved in butanol production was also cloned from *C. acetobutylicum *EA 2018 and sequenced, the comparative analysis showed that the *sol *operon of *C. acetobutylicum *EA 2018 was also 100% identical to that of *C. acetobutylicum *ATCC 824 (Accession number NC_001988 for ATCC 824 mega-plasmid sequence) [24]. The analysis demonstrated that EA 2018 and ATCC 824 belong to the same species.

**Figure 1:**
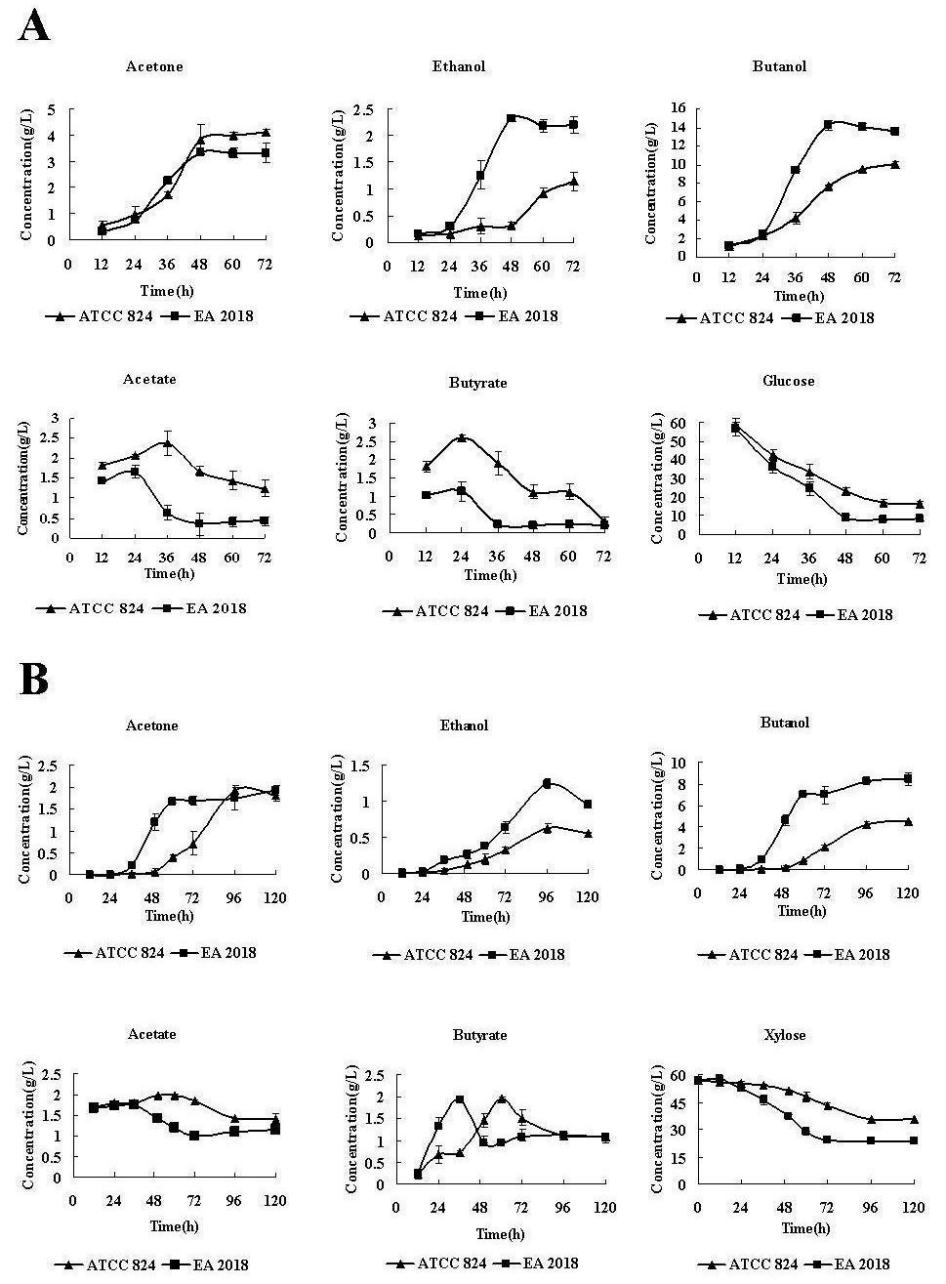
**Solvent and acid production, sugar utilization of *C. acetobutylicum *EA 2018 versus *C. acetobutylicum *ATCC 824 in P2 medium contained with 6% glucose (A) and 6% xylose (B)**.

Fermentation experiments were performed to compare the solvent production patterns of *C. acetobutylicum *EA 2018 and ATCC 824. EA 2018 exhibited higher solvent formation capacity than ATCC 824 strain in either 6% (*w/v*) glucose or xylose media (Figure 1A, B). After 48 h fermentation, 8.3 g/L glucose remained in the EA 2018 culture, while 16.5 g/L glucose still remained in the ATCC 824 culture after 72 h fermentation (Figure 1A). After 96 h fermentation, 23.6 g/L xylose was present in the EA 2018 culture, while 35.7 g/L xylose was still remained in the ATCC 824 culture (Figure 1B). In most *C. acetobutylicum *strains, solvent formation is always coupled with initiation of sporulation [10]. However, after fermentation of 72 h, there was no spore found in EA 2018 cultures, while significant spores were found in ATCC 824. With its higher solvent production and non-spore forming characteristics, EA 2018 strain could be an excellent strain for industrial application.

### Overview of *C. acetobutylicum *EA 2018 genome

**Figure 2:**
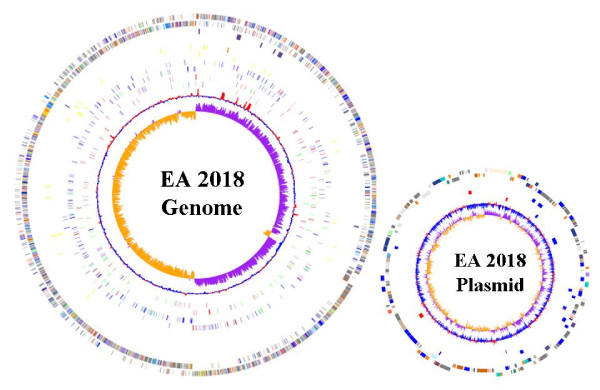
**Atlas of the chromosome and the megaplasmid of *C. acetobutylicum *EA 2018 and its comparison with *C. acetobutylicum *ATCC 824**. Moving inside, each concentric circle represents genomic data for *C. acetobutylicum *EA 2018 and its comparison with *C. acetobutylicum *ATCC 824. For chromosome atlas, the outer circle illustrates predicted coding sequences on the plus and minus strands, respectively, colored by functional categories according to COG classification. The 2nd circle represents EA 2018 variation genes compared with ATCC 824. The 3rd circle displays IS elements in EA 2018. The 4th circle shows rDNA genes in EA 2018, distinguished by plus strand (gold) and minus strand (red). The 5th circle shows tRNA genes in EA 2018, distinguished by plus strand (pink) and minus strand (blue). The 6th circle represent GC content, red for GC content above average and blue for GC content below average. The 7th circle (innermost) represents GC skew (G-C)/(G+C) calculated using a 100 kb window. For megaplasmid atlas, the outer circle illustrates predicted coding sequences on the plus and minus strands, respectively, colored by functional categories according to COG classification. 2nd circle represents EA 2018 variation genes compared with ATCC 824. 3th circle represent GC content, red for GC content above average and blue for GC content below average. The 4th circle (innermost) represents GC skew (G-C)/(G+C) calculated using a 100 kb window.

For better understanding of the genetic basis of improved butanol producing characteristics in EA 2018, the whole genome of EA 2018 was sequenced. The genome has a circular chromosome consisting of 3,940,230 bp with an average G+C content of 30.93% and a circular megaplasmid of 191,996 bp with an average G+C content of 30.91%. The genome finishing procedures were listed in Additional file 1, 2, 3. A total of 3923 protein coding sequence (CDS) including 3,746 in chromosome and 176 in megaplasmid were indentified in the EA 2018 genome, representing 86.8% of the genome and 83.8% of the megaplasmid, respectively. The functional classification of all EA 2018 genes was listed in Table 1. There are 11 copies of rDNA operons and a total of 75 tRNA genes scattered over the EA 2018 genome. Genomic comparison with type strain ATCC 824 revealed the highly conserved gene content and gene order between these two strains. The base numbering start point of EA 2018 were chosen as the same site in ATCC 824 (Figure 2).

**Table 1:** Function Classification of EA 2018 genes

Function	Numbers in plasmid	Numbers in genome	Function	Numbers in plasmid	Numbers in genome
Energy production and conversion	12	121	Cell envelope biogenesis, outer membrane	10	182
Cell division and chromosome partitioning	3	38	Cell motility and secretion	1	92
Amino acid transport and metabolism	1	212	Posttranslational modification, protein turnover, chaperones	2	78
Nucleotide transport and metabolism	1	73	Inorganic ion transport and metabolism	6	93
Carbohydrate transport and metabolism	22	221	Secondary metabolites biosynthesis, transport and catabolism	3	27
Coenzyme metabolism	1	110	General function prediction only	16	321
Lipid metabolism	6	76	Function unknown	5	258
translation,ribosomal structure and biogenesis	0	159	Signal transduction mechanisms	4	126
transcription	22	243	Intracellular trafficking and secretion	0	14
DNA replication, recombination and repair	5	137	Defense mechanisms	5	106
			Not in this system	55	1059

### Comparative genomic analysis of EA 2018 and ATCC 824

The size of the EA 2018 chromosome is 650 bp smaller than that of ATCC 824, and the size of the EA 2018 megaplasmid is 4 bp smaller than that of ATCC 824. Compared with ATCC 824, a total of 46 deletion sites and 26 insertion sites were found across the EA 2018 genome, including 1 deletion site in the megaplasmid (Additional file 4). Among them, 55 sites are single nucleotide indel (*i.e. *insertion or deletion), and only 7 indel sites are larger than 100 bp. The largest insertion is 1768 bp in 1276337-1278091 which is located within a hypothetical protein gene (CEA_G1125, corresponding to CAC1113 in ATCC 824), while the largest deletion is 1812 bp in 1112030-1112031 which is located within a predicted member protein gene (CEA_G0978, corresponding to CAC0966 in ATCC 824). Apart from those indels, 440 single nucleotide variations (SNVs) were identified between the two chromosomes, and 11 SNVs were identified between the megaplasmid of EA 2018 and ATCC 824 (Additional file 5). The 523 variations (including 72 indels and 451 SNVs) affected a total of 229 protein encoding genes (including 10 genes in megaplasmid) and 38 predicted promoters (including 1 in megaplasmid) in the EA 2018 genome. Excluding the synonymous substitution, a total of 189 proteins had amino acids changes, while 47 of those proteins were with similar amino acid variations (Additional file 6). The 38 genes with their putative promoters affected were listed in Additional file 7. In Table 2 and 3, variations within putative promoters and genes related to solvent formation, xylose utilization, and spore formation (*i.e. *three key aspects related to enhanced butanol production in EA 2018) were listed.

**Table 2:** EA 2018 gene variations associated with key phenotypes

Gene locus in EA 2018	Gene locus in ATCC 824	Gene variation sites in EA 2018	Gene variation sites in ATCC 824	Protein variation sites in EA 2018	Protein variation sites in ATCC 824	Product name
**Solvent formation related genes**						

CEA_G1048	CAC1036	899(T)	899(C)	300(V)	300(A)	Pyruvate kinase
CEA_G1755	CAC1742	391(A)	391(G)	131(N)	131(D)	phosphotransacetylase
CEA_G2463	CAC2449	256(A)	256(G)	86(S)	86(G)	Predicted flavoprotein
CEA_G2485	CAC2471	439(A)	438-439(-)	147-150(R,L,P,I)	147-150 (G,C,L,Stop codon)	Transcriptional regulator, TetR/AcrR family
CEA_G2556	CAC2543	241-249 (G,T,A,G, A,T,C,A,T)	240-241(---)	81-83(V,D,H)	80-81(---)	Electron-transferring flavoprotein large subunit
CEA_G2806	CAC2798	286(A)	286(G)	96(M)	96(V)	NADH:flavin oxidoreductase
CEA_P0058	CA_P0059	754(A)	754(C)	252(N)	252(H)	Alcohol dehydrogenase
CEA_P0077	CA_P0078	91(A)	91(G)	31(T)	31(A)	acetyl-CoA acetyltransferase
CEA_P0140	CA_P0141	566(T)	566(C)	189(I)	189(T)	Periplasmic hydrogenase small subunit, dehydrogenase

**Substrate utilization related genes**						

CEA_G0239	CAC0234	1072(T)	1072(C)	358(Stop codon)	358(Q)	PTS system, fructoso-specific IIBC component
CEA_G1333	CAC1319	5(T)	5(C)	2(I)	2(T)	Glycerol uptake facilitator protein, GLPF
CEA_G1472	CAC1456	974(T)	974(A)	325(M)	325(K)	Sugar-binding periplasmic protein
CEA_G2622	CAC2613	270(T)	270(G)	90(C)	90(W)	Transcriptional regulators of NagC/XylR family
CEA_G2919	CAC2912	97(A)	97(C)	33(T)	33(P)	Sugar-binding periplasmic protein
CEA_P0052	CA_P0053	317(C)	317(T)	106(P)	106(L)	Xylanase, glycosyl hydrolase family 10

**Sporulation related genes**						

CEA_G0080	CAC0080	1160(A)	1159-1160(-)	387-392(N,I,Q,D,L,Stop codon)	387-391(I,Y,K,I,Y,K)	Histidine kinase-like ATPase
CEA_G0656	CAC0644	1226(T)	1226(G)	409(V)	409(G)	Spore germination protein gerKA
CEA_G0710	CAC0699	416(C)	416(T)	139(T)	139(I)	Spore photoproduct lyase, splB
CEA_G2066	CAC2052	688(A)	687-688(-)	230-248 (18 amino acid)	230-238 (7 amino acid and a Stop Codon)	DNA-dependent RNA polymerase sigma subunit
CEA_G3736	CAC3729	374(T)	374(C)	125(L)	125(P)	Stage 0 sporulation J, ParB family of DNA-binding proteins
CEA_P0016	CA_P0017	11,245(G,A)	11,245(T,C)	4,82(E,G)	4,82(A,V)	Spore germination protein, GRKB
CEA_P0019	CA_P0020	1120(C)	1120(T)	407(A)	407(V)	Spore germination protein, GRKA
CEA_P0021	CA_P0022	-151(T)	104(C)	1(---)	1-85(85 amino acids insertion)	Spore germination protein, GRKB

Numbers in gene or protein variation sites lines indicated the variation sites in genes; the letters in bracket means the corresponding variation bases or amino acid; the symbol "-" means deletion in genes; the symbol "---" means consecutive deletion in genes; "No" means no amino acid variation; "Stop codon" means this site is mutated to stop codon.

**Table 3:** EA 2018 putative promoter variations related to key phenotypes

Gene locus in EA 2018	Gene locus in ATCC 824	Gene variation sites in EA 2018	Gene variation sites in ATCC 824	Product Name
**Solvent formation related genes**				

CEA_G0028	CAC0028	-12(T)	-12(C)	Hydrogen dehydrogenase
CEA_P0034	CA_P0035	-84(T)	-84(C)	Aldehyde-alcohol dehydrogenase, ADHEII

**Substrate utilization related genes**				

CEA_G3043	CAC3037	-80(T)	-80(C)	Catabolite control protein, LacI family transcriptional regulator
CEA_G3455	CAC3451	-84(T)	-84(G)	Sugar/Na+(H+) simporter
CEA_G1086	CAC1075	-56(T)	-56(G)	Beta-glucosidase family protein
CEA_G1365	CAC1351	-97(T)	-97(C)	Periplasmic sugar-binding protein

**Sporulation related genes**				

CEA_G1634	CAC1620	-136(T)	-136(G)	Small acid-soluble spore protein
CEA_G3742	CAC3735	-(8-7)(-)	-7(C)	Predicted RNA-binding protein Jag, SpoIIIJ-associated

Numbers in gene or protein variation sites lines indicated the variation sites in genes; the letters in bracket means the corresponding variation bases or amino acid; the symbol "-" means deletion in genes.

### Comparative transcriptomic analysis of EA 2018 and ATCC 824

To further explore the molecular mechanism of enhanced butanol production in the EA 2018 strain, DNA microarray of *C. acetobutylicum *were manufactured and used for a comparative analysis between EA 2018 and ATCC 824. The complete set of DNA array data was available in Additional file 8. Microarray analysis showed that a total of 2215 genes were differentially regulated at transcriptional level in at least 1 cultivation time point. Among them, differentially regulated genes related to some important metabolic pathways were listed in Table 4. And some putative promoter variation genes such as *adhEII *were found with differential expression level in the EA 2018 strain (Table 5). The detailed comparative analysis of transcriptomic data, along with genomic data (*i.e. *variation gene sequence) and biochemical phenotypes will be provided below from three key aspects related to enhanced butanol production (*i.e. *solvent formation, xylose utilization, and spore formation).

**Table 4:** List of differentially regulated genes in key functional groups.

Gene locus in EA 2018	Gene locus in ATCC 824	**9 h (Log**_**2**_**Ratio 2018/824)**	**13 h (Log**_2_**Ratio 2018/824)**	**17 h (Log**_2_**Ratio 2018/824)**	**21 h (Log**_2_**Ratio 2018/824)**	**24 h (Log**_2_**Ratio 2018/824)**	**30 h (Log**_2_**Ratio 2018/824)**	Product Name
**Carbohydrate transportant and metabolism**								

CEA_G0343	CA_C0332	-2.97436	-3.22501	-2.37758	-2.08064	-2.57556	-1.49158	Beta-mannanase
CEA_G0501	CA_C0490	2.331505	2.410311	2.244172	2.361906	2.703307	2.474026	sugar kinase, N-terminal region - uncharacterized protein
CEA_G0552	CA_C0539	-3.20909	-3.00324	-2.93013	-2.29917	-3.34939	-3.01922	ChW repeat-containing mannanase ManB
CEA_G0553	CA_C0540	-3.097	-3.03398	-2.7227	-2.28306	-3.3665	-2.72934	ChW repeat-containing mannanase ManB
CEA_G1677	CA_C1664	1.744408	2.013214	2.53088	1.134795	1.816259	2.062687	glycogen phosphorylase
CEA_G2012	CA_C1997	3.052977	3.503142	3.004392	2.8712	2.075025	1.372718	glycosyltransferase
CEA_G2022	CA_C2007	3.179932	3.308867	2.812136	2.509044	1.656072	1.958591	glycosyltransferase
CEA_G2528	CA_C2514	2.588809	2.748177	3.625871	4.329215	2.708288	1.042021	Beta galactosidase
CEA_G2815	CA_C2807	2.40568	2.124155	2.476058	2.653992	2.808752	1.737289	endo-1,3(4)-beta-glucanase family protein 16
CEA_G2818	CA_C2810	1.835243	2.287891	2.800041	3.929157	3.739976	4.256289	glucoamylase family protein
CEA_G3051	CA_C3045	-2.41601	-2.05151	-2.19775	-2.37262	-2.3321	-2.16444	PHP family hydrolase
CEA_G3060	CA_C3054	-3.63818	-4.38617	-4.64833	-5.02922	-5.12371	-3.50751	phosphoheptose isomerase
CEA_G3426	CA_C3422	-1.07396	-2.79221	-3.22016	-2.15086	-2.69786	-1.37931	sugar:proton symporter (xylulose)
CEA_P0052	CA_P0053	2.477697	2.868013	3.649329	3.695172	3.721454	5.987476	xylanase
CEA_P0053	CA_P0054	4.25084	2.735168	2.447001	3.424303	3.598697	5.185082	xylanase/chitin deacetylase family protein
CEA_P0065	CA_P0066	3.062167	1.145145	2.159875	4.902259	3.81378	2.953822	mannose-specific phosphotransferase system component IIAB
CEA_P0066	CA_P0067	2.925021	1.579742	2.174524	4.883877	3.683632	2.826095	mannose/fructose-specific phosphotransferase system component IIC
CEA_P0067	CA_P0068	3.006142	1.74298	2.611356	4.717371	3.502957	2.456002	mannose-specific phosphotransferase system component IID
CEA_P0115	CA_P0116	1.777221	2.342324	2.902238	3.126694	3.321994	5.378643	xylanase

**Amino acid transport and metabolism**								

CEA_G0180	CA_C0176	2.56498	2.063235	2.36635	2.08086	3.251555	3.705152	oligopeptide-binding protein, periplasmic component
CEA_G0327	CA_C0316	-4.09229	6.429377	7.957548	6.881528	4.185199	2.037144	ornithine carbomoyltransferase
CEA_G0390	CA_C0380	-4.23921	5.06975	5.365591	5.142555	3.394778	2.099342	periplasmic amino acid-binding protein
CEA_G0984	CA_C0973	-4.67305	6.390879	8.78089	7.141168	3.889621	2.522783	argininosuccinate synthase
CEA_G0985	CA_C0974	-4.03279	6.033303	8.738086	7.66813	4.662246	3.462605	argininosuccinate lyase
CEA_G2392	CA_C2377	-4.81354	-4.50847	-4.89131	-5.25303	-5.6598	-6.94396	oligopeptide ABC-type transporter, periplasmic binding component (frameshift)
CEA_G2403	CA_C2388	-3.653	6.58659	9.490741	7.214547	3.839977	1.277684	acetylornithine aminotransferase
CEA_G2405	CA_C2390	-4.029	7.044851	9.332151	8.593887	5.225523	1.729156	N-acetyl-gamma-glutamyl-phosphate reductase
CEA_G2406	CA_C2391	-3.63924	6.659273	9.430206	8.041522	4.889782	1.658329	bifunctional ornithine acetyltransferase/N-acetylglutamate synthase protein
CEA_G2531	CA_C2517	2.601974	2.875402	3.244092	4.199853	5.407919	5.659197	extracellular neutral metalloprotease, NPRE
CEA_G3059	CA_C3053	-3.80847	-4.46351	-4.85998	-5.20027	-5.64176	-4.16398	histidinol phosphatase related enzyme
CEA_G3625	CA_C3618	-2.33371	4.975742	4.579403	4.356899	3.79613	1.400104	ABC-type polar amino acid transport system, ATPase component
CEA_G3626	CA_C3619	-2.49499	4.94909	4.797836	4.831871	4.227636	1.349231	amino acid ABC transporter permease
CEA_G3627	CA_C3620	-2.79225	4.869307	2.77014	4.039679	3.923914	1.658129	amino acid ABC transporter periplasmic-binding protein
CEA_G3629	CA_C3622	3.744723	1.616914	-1.60643	-2.30924	-3.70552	-4.20925	benzoyl-CoA reductase/2-hydroxyglutaryl-CoA dehydratase
CEA_G3648	CA_C3641	3.877563	5.273677	6.532841	7.05381	6.150331	2.645978	oligopeptide ABC transporter, ATPase component
CEA_G3649	CA_C3642	3.862767	5.064748	6.213521	7.100308	6.123778	2.328286	oligopeptide ABC transporter, ATPase component
CEA_G3651	CA_C3644	3.540737	4.731217	4.796332	6.751584	6.151055	3.301733	oligopeptide ABC transporter, permease component

**Lipid transport and metabolism**								

CEA_P0077	CA_P0078	-1.03663	-2.39938	-2.90422	-5.61457	-6.37659	-6.669	acetyl-CoA acetyltransferase
CEA_G0500	CA_C0489	2.843362	2.727643	3.104514	2.258672	2.789674	2.994569	4'-phosphopantetheinyl transferase
CEA_G2024	CA_C2009	3.02793	3.412819	2.753976	2.880761	2.465438	1.317947	3-hydroxyacyl-CoA dehydrogenase
CEA_G2027	CA_C2012	3.139119	3.519089	2.760385	2.466674	1.747722	1.295802	enoyl-CoA hydratase
CEA_G2023	CA_C2008	3.21547	3.393795	2.65468	2.624962	1.849978	1.696553	3-oxoacyl-(acyl-carrier-protein) synthase
CEA_G0825	CA_C0814	3.447389	2.690101	3.341654	3.492821	2.738719	2.617536	3-oxoacyl-
CEA_G3630	CA_C3623	3.78405	1.698615	-1.62094	-2.13197	-3.66189	-4.47732	2-hydroxyglutaryl-CoA dehydratase activator

**Coenzyme transport and metabolism**								

CEA_G2539	CA_C2526	-5.80423	-4.29934	-2.18936	-3.20732	-2.04208	-2.09088	6-pyruvoyl-tetrahydropterin synthase related protein
CEA_G0110	CA_C0109	-1.93293	-1.10289	-4.51818	-4.22606	-3.40598	-5.95312	sulfate adenylyltransferase subunit 2
CEA_G2240	CA_C2226	1.175604	1.630568	1.933437	3.44313	4.132037	3.011011	branched-chain amino acid aminotransferase
CEA_G2817	CA_C2809	1.909506	2.362089	1.59545	3.297164	2.183049	1.029092	HD superfamily hydrolase
CEA_G2037	CA_C2022	2.45013	1.945668	2.521958	2.427533	2.187431	3.238565	molybdopterin biosynthesis protein MoaB
CEA_G3633	CA_C3626	2.627562	1.339719	-1.38437	-2.32524	-3.57634	-5.06491	GTP cyclohydrolase I
CEA_G2036	CA_C2021	2.849463	2.144804	2.666979	4.297463	3.319713	3.863173	molybdopterin biosynthesis protein MoeA
CEA_G2009	CA_C1994	3.136184	3.75827	3.408469	2.84809	1.881193	1.268359	molybdopterin biosynthesis protein MoaB
CEA_G2035	CA_C2020	3.146626	3.515592	5.182665	4.677354	4.692789	4.012931	molybdopterin biosynthesis protein MoeA
CEA_G3631	CA_C3624	3.416604	1.490697	-1.38827	-2.23319	-3.83641	-6.15768	6-pyruvoyl-tetrahydropterin synthase

**Signal transduction**								

CEA_G0078	CA_C0078	-4.99228	-8.71284	-9.43594	-9.21854	-9.14927	-7.97564	putative accessory gene regulator protein
CEA_G3328	CA_C3325	-1.00804	-2.85267	-4.3502	-5.19446	-4.39209	-5.05262	periplasmic amino acid binding protein
CEA_G0921	CA_C0909	-2.84748	-1.88833	-1.93256	-1.50557	-2.64087	-2.89144	methyl-accepting chemotaxis protein
CEA_G2085	CA_C2071	2.455498	2.263411	2.413649	1.803983	2.20351	2.550339	Spo0A protein
CEA_G2422	CA_C2407	3.784844	1.475039	1.927948	1.823945	2.526403	2.502499	CheY-like domain-containing protein
CEA_G0448	CA_C0437	2.192653	1.378372	2.665966	1.827148	2.252286	2.342264	sensory transduction histidine kinase
CEA_G3025	CA_C3019	-2.65448	3.491201	3.449102	2.735229	1.336433	1.266833	sensory transduction protein
CEA_G0296	CA_C0289	1.792323	1.978035	2.438903	2.751395	3.435751	3.052853	response regulator
CEA_G2782	CA_C2774	1.625932	1.895382	3.060796	2.846554	2.092881	1.726376	methyl-accepting chemotaxis protein
CEA_G0334	CA_C0323	3.362504	2.662176	2.652177	3.222052	4.952742	2.037345	sensory transduction histidine kinase
CEA_G3627	CA_C3620	-2.79225	4.869307	2.77014	4.039679	3.923914	1.658129	amino acid ABC transporter periplasmic-binding protein
CEA_G0390	CA_C0380	-4.23921	5.06975	5.365591	5.142555	3.394778	2.099342	periplasmic amino acid-binding protein

**Energy production and convertion**								

CEA_G1083	CA_C1072	3.271648	3.230747	4.254617	2.59071	2.723209	3.766376	Fe-S oxidoreductase
CEA_G2012	CA_C1997	3.052977	3.503142	3.004392	2.8712	2.075025	1.372718	glycosyltransferase
CEA_G2015	CA_C2000	2.963078	3.638083	2.745804	2.983214	2.587284	2.204476	indolepyruvate oxidoreductase subunit beta
CEA_G2016	CA_C2001	2.832561	3.688549	3.007107	3.355002	2.718563	2.206461	indolepyruvate ferredoxin oxidoreductase, subunit
CEA_G2022	CA_C2007	3.179932	3.308867	2.812136	2.509044	1.656072	1.958591	glycosyltransferase
CEA_G2025	CA_C2010	3.282342	3.533526	2.84142	3.003615	2.50221	2.005231	Fe-S oxidoreductase
CEA_G2555	CA_C2542	8.22595	5.876659	5.423124	5.135223	4.224852	4.280766	FAD/FMN-containing dehydrogenase
CEA_G2556	CA_C2543	8.451735	6.100494	5.401602	5.24343	4.426238	3.987954	electron-transferring flavoprotein large subunit
CEA_G2557	CA_C2544	7.904188	6.040402	4.848558	4.578106	4.214698	3.969443	electron-transferring flavoprotein small subunit
CEA_G3411	CA_C3408	-2.71993	-3.64684	-4.53359	-4.7967	-4.89809	-4.45863	NADH oxidase

**Cell mobility**								

CEA_G0921	CA_C0909	-2.84748	-1.88833	-1.93256	-1.50557	-2.64087	-2.89144	methyl-accepting chemotaxis protein
CEA_G3572	CA_C3565	1.783589	1.966743	1.626123	1.303128	1.522492	1.56009	cell adhesion domain-containing protein
CEA_G3091	CA_C3085	1.669031	1.879947	1.641572	3.329325	2.811493	2.558158	TPR repeat-containing cell adhesion protein
CEA_G3092	CA_C3086	1.689695	1.7646	1.823344	3.167841	2.629106	2.423856	cell adhesion domain-containing protein
CEA_G2782	CA_C2774	1.625932	1.895382	3.060796	2.846554	2.092881	1.726376	methyl-accepting chemotaxis protein
CEA_P0159	CA_P0160	2.878477	3.739495	3.515178	3.50976	3.19802	3.547315	cell-adhesion domain-containing protein

**Table 5:** DNA and transcriptional variations of putative promoter variations between EA 2018 and ATCC 824

Gene locus in EA 2018	Gene locus in ATCC 824	Gene varition site in 2018	Gene varition site in 824	**9 h (Log**_**2**_**Ratio 2018/824)**	**13 h (Log**_**2**_**Ratio 2018/824)**	**17 h (Log**_**2**_**Ratio 2018/824)**	**21 h (Log**_**2**_**Ratio 2018/824)**	**24 h (Log**_**2**_**Ratio 2018/824)**	**30 h (Log**_**2**_**Ratio 2018/824)**	Product Name
CEA_G0334	CA_C0323	-96(A)	-(97-96)(-)	3.362504	2.662176	2.652177	3.222052	4.952742	2.037345	Sensory transduction histidine kinase
CEA_G0390	CA_C0380	-135,-131(A,A)	-135,-131(T,T)	-4.23921	5.06975	5.365591	5.142555	3.394778	2.099342	Periplasmic amino acid-binding protein
CEA_G2504	CA_C2490	-(13-12),-53(-,T)	-12,-(54-53)(C,-)	-2.39555	-3.35613	-3.80832	-2.2032	-2.7562	-1.10185	Xre family DNA-binding domain and TPR repeats containing protein
CEA_G3701	CA_C3694	-43(T)	-43(C)	4.045439	3.430925	3.859781	3.69948	4.131629	3.75554	TPR-repeat-containing protein
CEA_P0034	CA_P0035	-84(T)	-84(C)	3.331902	5.740285	6.007483	8.056546	6.576379	7.664691	Aldehyde-alcohol dehydrogenase, AdhEII

### Changes in expression of *adhEII*, *spo0A *and hydrogenase gene may contribute to enhanced solvent formation in EA 2018

**Figure 3:**
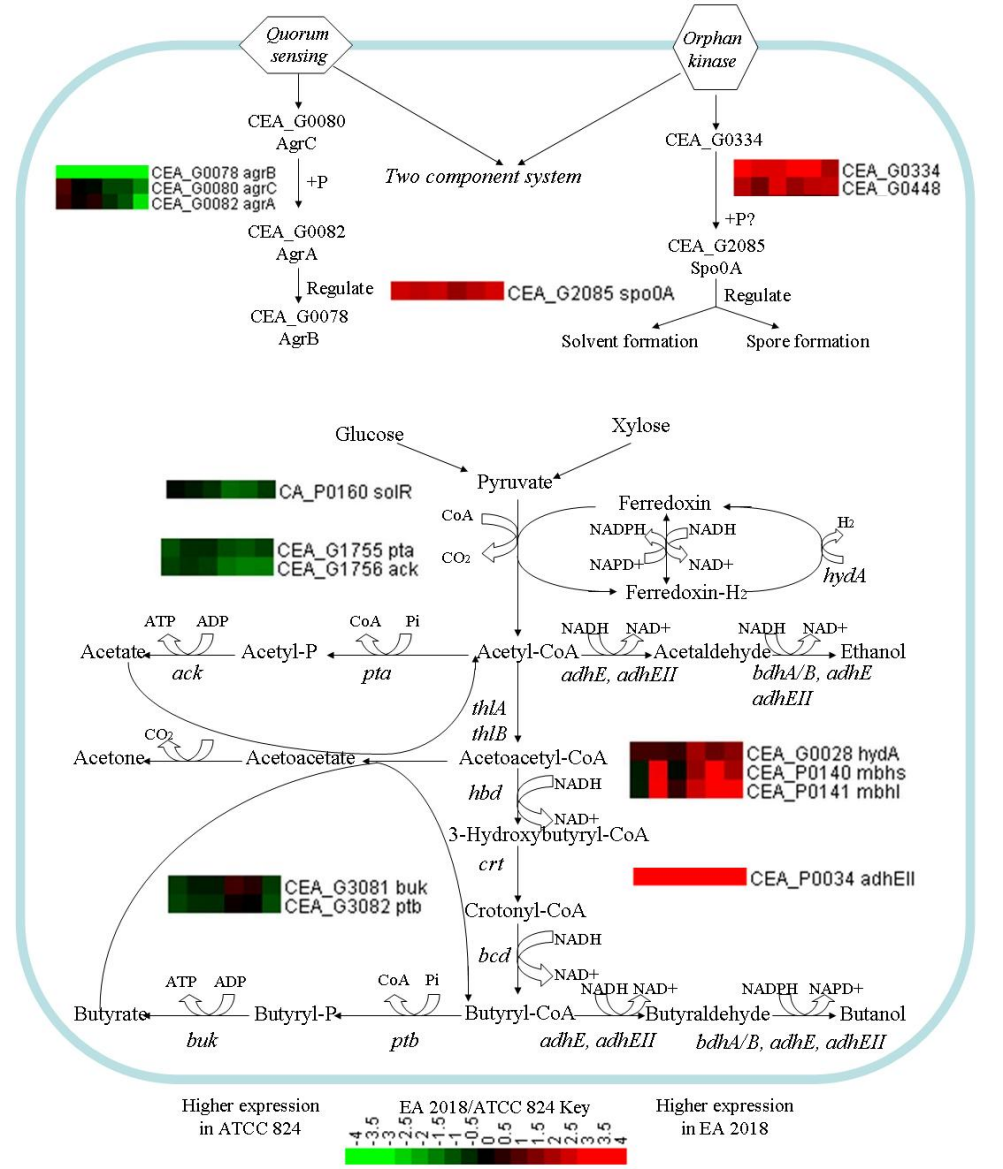
**Expression profiles of variation genes involved in sporulation related two-component system and solvent formation pathway of *C. acetobutylicum *EA 2018 versus ATCC 824**.

Comparative genomic analysis identified a set of solvent-relevant genes with variations within their coding sequences (Table 2). And most of which were SNV variations, such as genes encoding phosphotransacetylase and acetyl-CoA acetyltransferase. In addition, comparative genomics analysis also identified some variations up-stream of solvent-relevant genes (Table 3), which could potentially affect expression level of these genes. For example, a SNV site was found 84 bases upstream of the start codon of the AdhEII encoded gene CEA_P0034 (CA_P0035 in ATCC 824), under the *s*_*1 *_transcription start point [25]. Consistent with the variations at genomic level, we also found that transcription level of solvents formation genes, such as *adhEII *were highly expressed in EA 2018 relative to ATCC 824(Table 5 and Figure 3).

Spo0A is a central regulator of sporulation and solvent formation in *C. acetobutylicum*. Previous studies showed that *C. acetobutylicum spo0A *inactivation mutant stops producing spores and solvent, while over-expression of *spo0A *gene can enhance solvent production since 0A binding boxes have been identified in the promoter regions of solvent formation genes [26]. Therefore, it is speculative that the higher expression of *spo0A *could be one of the reasons for the higher butanol formation in EA 2018. Comparative transcriptomic analysis confirmed our speculation that higher transcriptional level of *spo0A *was found in EA 2018 (Table 4 and Figure 3). There were three ways for Spo0A to be phosphorylated (*i.e. *by a sensory kinase; through a novel phosphorylation system; by butyryl-P or acetyl-P) [27]. Using trans-membrane domain along with transcriptional analysis, four orphan kinases (CAC0437, CAC0323, CAC0903, and CAC2730) were identified as plausible kinases that might phosphorylate Spo0A in *B. subtilis *[27]. In our study, we also found that the transcriptional level of an orphan kinase CEA_G0344 (corresponding to CAC0323 in ATCC 824) was higher in EA 2018 (Figure 3), and the result is consistent well with the *spo0A *gene expression data.

During solvent fermentation process in *C. acetobutylicum*, a considerable amount of NADH was consumed by hydrogenase via reduced Fd (FeH_2_) to form hydrogen [28]. Previous reports showed that butanol production by *C. acetobutylicum *can be elevated by inhibiting hydrogen formation through adding viologen dyes or increasing hydrogen partial pressure [28], and knockdown of *hupCBA *cluster which encoded hydrogen uptake genes in *C. saccharoperbutylacetonicum *strain N1-4 decreased butanol formation (to 75.6% compared to the control strain) successfully [29]. Biochemical analysis showed that hydrogen formation in EA 2018 was nearly 29% lower than in ATCC 824 (Table 6). Interestingly, comparative genomic analysis also revealed SNVs in NiFe-hydrogenase coded gene CEA_P0140 (CA_P0141 in ATCC 824) and in the promoter of Fe-only hydrogenase coded gene CEA_G0028 (CAC 0028 in ATCC 824) (Table 2, 3). The variation site of CEA_G 0028 was located on the 12^th ^base upstream of the start codon of *hydA *and altered the ribosome binding site (RBS) of this important gene (GGGAGG in ATCC 824 versus AGGAGG in EA 2018). In addition, the higher expression level of hydrogen uptake genes *mbhs *and *mbhl *were also revealed in EA 2018 (Figure 3). The result showed that hydrogen uptake could be an important factor for butanol formation, and increased expression level of hydrogen uptake gene *mbhs *and *mbhl *was closely correlated to the lower hydrogen formation in EA 2018, which can eventually help balance the NAD(P)H needed for higher production of butanol.

**Table 6:** Hydrogen production of *C. acetobutylicum *EA 2018 and *C. acetobutylicum *ATCC 824 in 6% glucose contained P2 medium

	Fermentation Time (h)	Glucose consumed (mM)	Hydrogen production (mM)	Hydrogen formation ratio (mM/100 mM glucose)
EA 2018	72	260.6 ± 3.7	244.1 ± 1.3	93.9 ± 1.6
ATCC 824	72	239.4 ± 5.0	315.4 ± 0.5	131.7 ± 3.1

Using non-replicating plasmid pO1X, putative solvent formation repressor *solR *gene was inactivated in ATTC 824, and its fermentation experiment revealed that more solvent were produced in the *solR *inactivation mutant [5]. Although there are different speculations on the function of SolR [25], it has been confirmed that low expression of *solR *will enhance solvent formation [26]. Transcriptomic analysis revealed a lower expression level of *solR*, especially in the solventogenic phase in EA 2018, which might be related to hyper-butanol formation (Figure 3).

It has been suggested that the onset of solvent production is closely related to the accumulation of acid end products [30], and the addition of acetate and butyrate might result in a rapid induction of solventogenesis [31]. For example, it was reported that the concentration of undissociated butyric acid might play an important role in the induction of solventogenesis [32]. Transcriptomic analysis showed that expression of *ack*, *pta*, *buk *and *ptb *were all lower in EA 2018 than in ATCC 824 (Figure 3), consistent with the biochemical analysis (Figure 1). In addition, the results also suggested that the transition to the solventogenesis took place at a lower acetate and butyrate acid concentration in EA 2018 compared to ATCC 824.

### Analysis of substrate utilization genes and inactivation of CAC2613 revealed genetic bases of better xylose utilization in EA 2018

**Figure 4:**
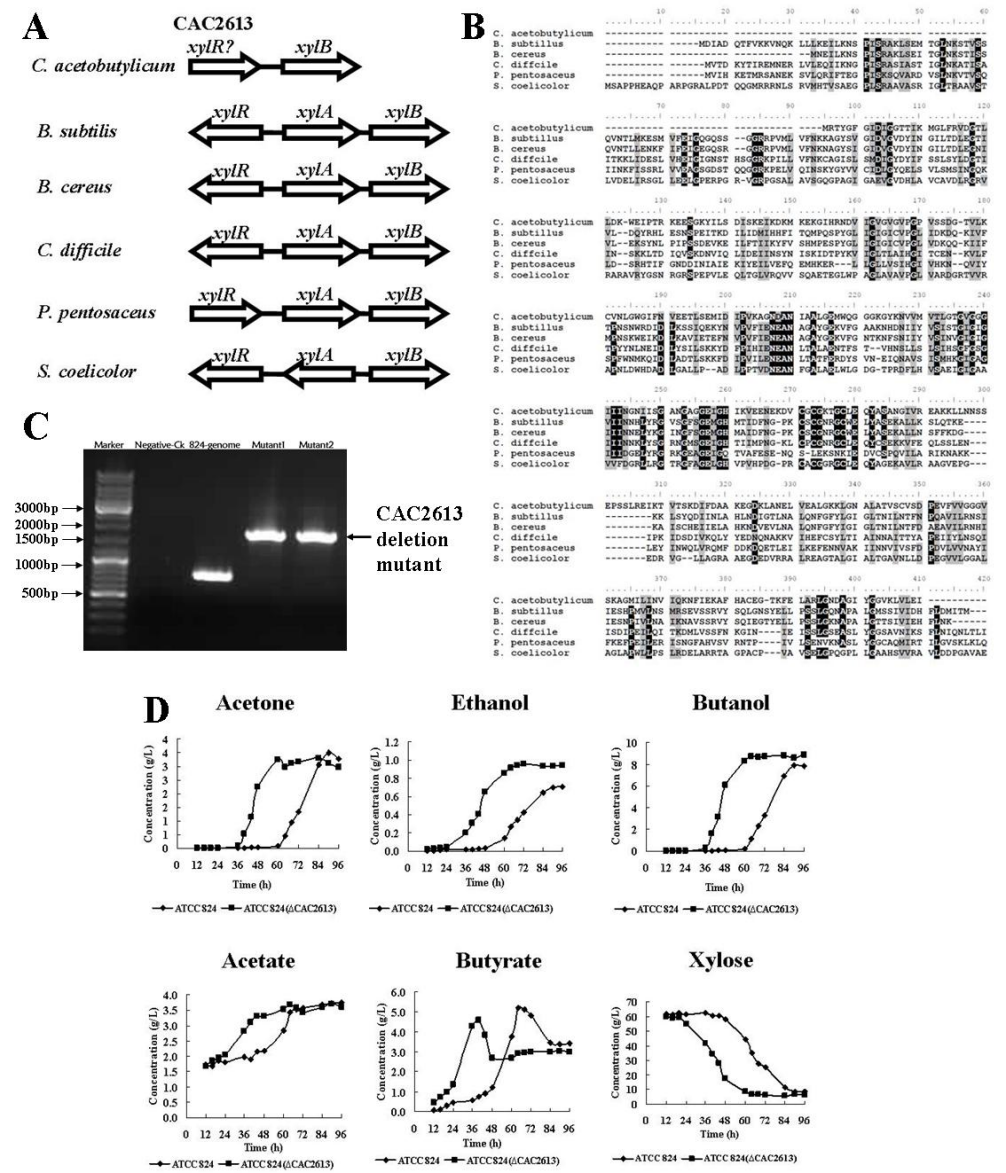
**Inactivation of putative *xylR *CAC2613 in ATCC 824 leaded to faster xylose utilization in *C. acetobutylicum***. A: location of CAC2613 in *C. acetobutylicum *and *xylR *in different organisms; B: Alignment of CAC2613 and *xylR *in different organisms; C: CAC2613 inactivation in ATCC 824 using Targetron system; D: Accurate pH-controlled fermentation profile of mutant strain versus wild type in 6% xylose contained P2 medium.

Solvent production from agriculturally based lignocellulosic substrates (*i.e. *cellulose or hemicellulose) was studied previously and results showed that a large part of the lignocellulosic substrates were hydrolyzed into glucose and xylose [33]. Therefore, utilization of these substrates, especially xylose, can be important in determining the efficiency of solvent production. Comparative genomic analysis identified several mutations in the putative promoters and within the coding region of genes which might be involved in substrates utilization (Table 2, 3). Among them, three out of seven mutated genes encode sugar-binding periplasmic proteins. One interesting gene was CEA_G2622 (CAC2613 in ATCC 824), which encodes a transcriptional regulator of NagC/XylR family and the sequence variation could cause a putative W90C substitution. The gene is located on the upstream of *xylB *(xylulose kinase) gene (Figure 4A). Since most of the known *xylR *genes in other AT-rich gram-positive species such as *B. subtillus *and *C. difficile *were located upstream of *xyl *operon [34,35], we speculated that this gene (CEA_G2622) may function similarly as *xylR *in EA 2018 (Figure 4B). Transcriptomic analysis showed that even in the glucose-based medium, the expression level of *xylB *was higher in EA 2018 (Figure 3). However, evidence is still needed to confirm the direct regulatory function of CEA_G2622 on *xyl *operon. To do so, we disrupted CAC2613 gene in *C. acetobutylicum *ATCC 824 (corresponding to CEA_G2622 in EA 2018) using Targetron system (Figure 4C). Batch fermentation showed that xylose utilization in CAC2613 disrupted mutant was faster than ATCC 824 (constant pH 5.0). In addition, the time of butanol formation and acids reassimilation in the mutant were 24 h earlier than ATCC 824 strain, although the final concentration of end products and xylose were nearly the same (Figure 4D). The similarities, in terms of the time of butanol formation and acid reassimilation, between EA 2018 and the CAC2613 disrupted ATCC 824 derived mutant, suggested that better xylose utilization in EA 2018 could be related to the mutation in CEA_G2622 (CAC2613 in ATCC 824).

**Figure 5:**
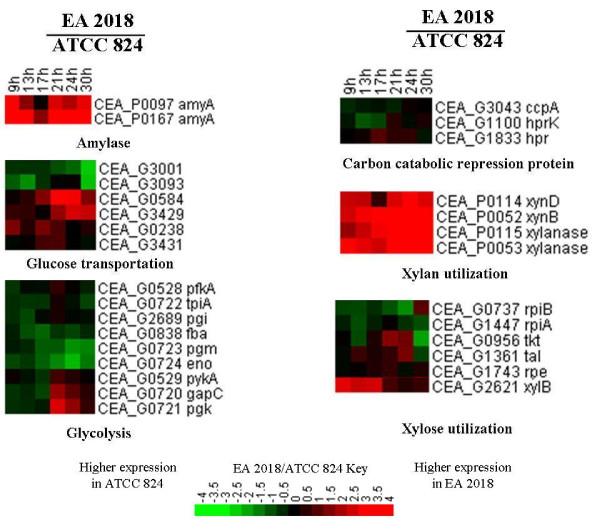
**Expression profiles of substrate utilization related genes**. Gene functions were shown below the expression profile. Red and green indicated higher or lower expression, respectively.

Previous study showed that ATCC 824 harbors extracellular and cell bound xylanase activities when grown under xylose or glucose-based media, and most of the putative xylanase encoded genes were located on the megaplasmid [36]. Two endoxylanase genes, thermostable xylanase 10A gene (CA_P0053 in ATCC 824 and CEA_P0052 in EA 2018) and xylanase 10B gene (CA_P0116 in ATCC 824 and CEA_P0115 in EA 2018) located on mega-plasmid were identified in EA 2018 [37]. In addition, transcriptomic analysis showed higher expression level of those two genes in EA 2018 (Figure 5). Xylan is the major component of hemicelluloses. The higher expression level of xylanase in EA 2018 could make it suitable for hemicellulosic fermentation, and could offer potential economic benefits in the future [1].

Among all putative promoter variations, there were 4 sites which may affect substrate utilization in EA 2018 (Table 3). Among them, CEA_G3043 (CAC3037 in ATCC 824) gene that encodes a catabolite control protein (CcpA) has a variation site located on 80 bp upstream of the start codon. CcpA play an important role in catabolite repression and inactivation of this gene will release catabolic repression in many gram-positive organisms [8,38,39]. However, no significant regulation was observed for the expression level of CcpA gene in EA 2018, and the potential effect of the variation in *ccpA *promoter region still needs further investigation.

### *agrC *and sigma factor variations may involve in spore formation in EA 2018

**Figure 6:**
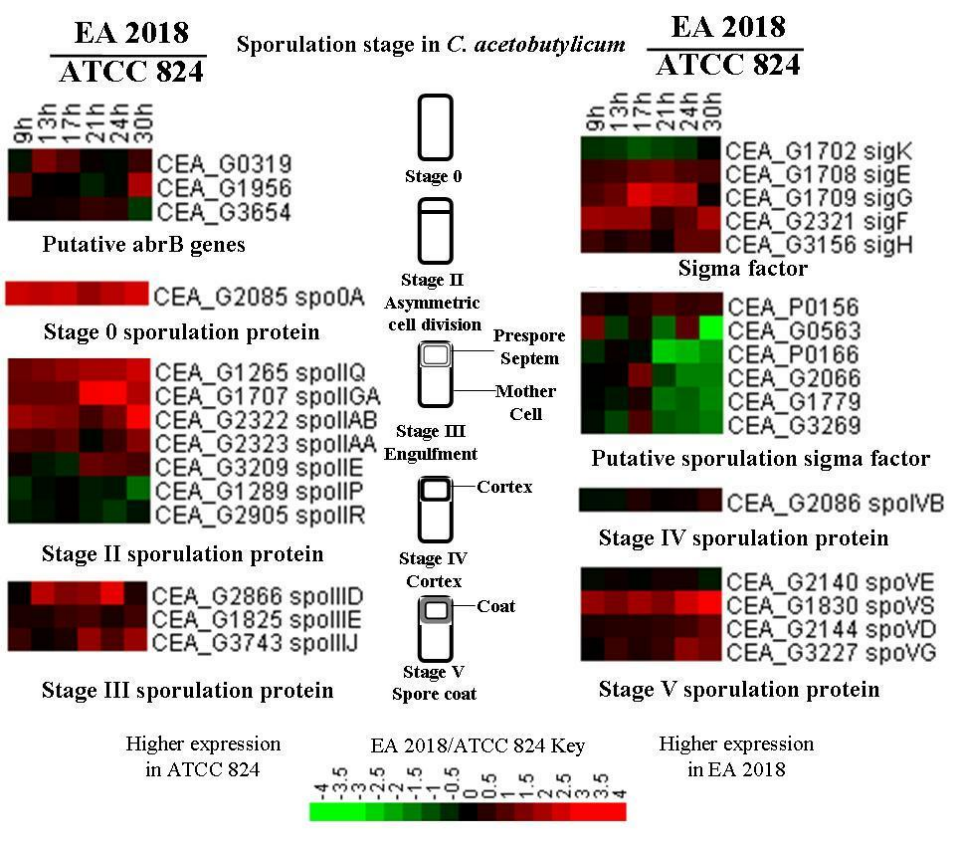
**Expression profiles of sporulation related genes**. Gene functions were shown below the expression profile. Red and green indicated higher or lower expression, respectively.

Comparative genomic analysis identified several genes which may be accounted for difference in terms of spore formation (Table 2). Among these muted genes, CEA_G2066 (CAC2052 in ATCC 824) encodes a putative sigma factor. It has been known that the transcription of this gene was closely related to spore-formation in ATCC 824 [40]. CEA_G2066 has a single nucleotide (A) insertion site in 687-688^th ^bases, which altered the C-terminal protein sequence. Transcriptomic analysis showed that the transcription level of CEA_G2066 and other 4 putative sporulation related sigma factors [40] was lower in EA 2018 at 21 h, 24 h and 30 h (Figure 6), which might contribute to non-sporulation property in EA 2018.

Quarum sensing is related to some important characteristics of bacteria, such as sporulation, virulence, and biofilm formation [41]. In *C. perfringens*, the virulence gene was regulated by *agr *system, and *agrBD *knockout mutant did not express theta-toxin gene, and transcription of the alpha- and kappa-toxin genes was also significantly decreased in the mutant strain [42]. A two-component system gene, CEA_G0080 (CAC0080 in ATCC 824), encoding a histidine kinase-like ATPase (AgrC), has a single nucleotide (A) insertion site in 1159-1160^th ^bases when compared with *agrC *gene in ATCC 824, which may truncate the protein encoding sequence. In addition, the expression level of *agrB *(CEA_G0078) was constantly lower in EA 2018 (Figure 3). Early studies suggested that deletion of any *agr *system genes could result in no spore formation in ATCC 824 [43]. Therefore, mutation in *agrC *gene and lower expression of *agrB *gene in EA 2018 might be responsible for to the non-sporulation property in EA 2018.

## Conclusion

A hyper-butanol, non spore-forming *C. acetobutylicum *EA 2018 strain we isolated previously can produce 10% more butanol than the type strain *C. acetobutylicum *ATCC 824 [23]. To seek molecular basis of these characteristics in EA 2018 strain, we completed the genome sequencing of this strain using 454 GS FLX pyrosequceing and performed a detailed genomic comparison with a *C. acetobutylicum *type strain ATCC 824. Although EA 2018 was found more than 99.8% identical to ATCC 824, 72 indels (*i.e. *insertions and deletions) and 451 SNVs were identified, some of which may be related to the enhanced butanol production in EA 2018. In addition, we performed a comparative transcriptomic analysis of *C. acetobutylicum *EA 2018 and ATCC 824 using oligonucleotide microarrays. The results showed that increased expression of several key genes related to solvent formation, and decreased expression of the acid formation related genes may be related to the enhanced butanol production in EA 2018. Furthermore, the results also showed that the variation in CEA_G2622 (CAC2613 in ATCC 824), a putative transcriptional regulator involved in xylose utilization, may be able to accelerate utilization of substarte xylose. The comparisons of hyper-butanol EA 2018 and type strain ATCC 824 at both genomic and transcriptomic levels not only improved our understanding of the hyper butanol-producing, xylose utilization as well as non-spore formation properties in EA 2018 strain, but also provided some useful clues for the future genetic modification of *C. acetobutylicum *to produce solvents, especially butanol more effectively.

## Methods

### Bacteria strain and genome sequencing

*C. acetobutylicum *EA 2018 (CCTCC M 94061) used for this study [22] is deposited in China Center for Type Culture Collection (http://www.cctcc.org/). *C. acetobutylicum *ATCC 824 was the wild type strain we purchased from American Type Culture Collection (ATCC) [23]. The *C. acetobutylicum *EA 2018 was grown anaerobically (Thermo Electron Crop., San Jose, USA). Colonies picked from Clostridia growth medium (CGM) plate were inoculated into 5 ml liquid CGM and cultured at 37°C overnight [44], and then the cells were transferred into 100 ml CGM and incubated at 37°C for 16-20 h until they reach late-exponential phase. Cells collected were used for chromosomal DNA isolation as described previously [45]. Roche 454 GS FLX pyrosequencing was used to sequence the DNA. A total of 60.3 Megabases was generated, with an average read length of 200 bp. The GS FLX reads were assembled into a total of 198 contigs using a GS de novo assembler, among them, 157 contigs are larger than 500 bp. The gaps were closed by PCR procedure using the ATCC 824 genome sequence as reference. The large PCR products were sequenced *via *primer walking. The whole sequence was assembled by using the software phredPhrap (http://www.phrap.org) and was visualized by Consed [46]. The low-quality sequences were verified by PCR resequencing using ABI 3730 (Applied Biosystem Inc.). The sequence accuracy of the final genome was 99.9919%. All the variation between EA 2018 and ATCC 824 were verified by PCR resequencing using ABI 3730.

### Genome annotation and bioinformatic analysis

CDSs were identified by combining the results of ZCURVE 1.0 [47] and Glimmer 3.2 (http://www.cbcb.umd.edu/software/glimmer). Transfer RNA genes were predicted by tRNAscan-SE [48]. Functional annotation of CDSs was performed through comparison with NCBI non-redundant protein database using BLASTP, followed by manual curation. Comparative genomic analysis was performed by using the Artemis Comparison Tool (ACT; http://www.sanger.ac.uk/resources/software/act/). The atlas of the genome is drawn by using GenomeViz1.1 [49].

### Nucleotide sequence accession number

The annotated genome sequence has been deposited into GenBank under accession no. CP002118 (Chromosome) and no. CP002119 (Plasmid).

### Oligonucleotide microarray experiments

Cells for RNA isolation were grown on P2 medium and collected at 9 h, 13 h, 17 h, 21 h, 24 h and 30 h by centrifugation at 4°C and 4500 × g for 10 min. Total RNA was extracted and purified by using Trizol (Invitrogen, Carlsbad, CA, USA) and RNeasy cleanup kit (Qiagen, Inc., Valencia, CA, USA) according to the manufacturer's protocol. The total RNA yield was quantified by spectrophotometric analysis (NanoDrop Technology, Cambridge, UK) and the quality was verified by gel electrophoresis. Agilent oligonucleotide microarrays technology was used for monochromic analysis, in which probes (size: 60 bp; three replicates for each ORF) from the two groups were labeled by incorporation of cyanine 3 (Cy3) (Agilent Technologies, Palo Alto, CA, USA). The experiment procedures and data normalization were performed using the methods described previously [50]. Average linkage hierarchical clustering was performed using Cluster 3.0, and gene clusters were visualized in Treeview [51].

### Oligonucleotide microarray accession number

The Oligonucleotide microarray data has been deposited into GEO under accession no. GSE23071.

### Gene disruption

Gene disruption in *C. acetobutylicum *ATCC 824 was performed as described previously [11]. The disruption procedures were shown in Additional file 9. The selected site for CAC2613 disruption was 532/533a, where the group II intron will insert into gene CAC2613 coding region between amino acid 532 and 533 sites in the antisense direction. The primers (CAC2613-532-533-IBS, CAC2613-532-533-EBS1d and CAC2613-532-533-EBS2), for retargeting the RNA portion of the intron for *C. acetobutylicum *CAC2613 gene disruption, are listed in Additional file 10. The 350 bp targetron fragment was obtained by PCR based on the plasmid pACD4K-C and protocol provided by the TargeTron™ Gene Knockout System Kit (Sigma-Aldrich, St Louis, MO, USA). The 350 bp PCR fragment was digested with *Xho*I and *Bsr*GI, and then inserted into pSY6 [11] digested with the same restriction enzymes, to generate the plasmid pSY6-2613. The plasmid pSY6-2613 was methylated in *E. coli *ER2275 (pANS1) first [52], and then electroporated into *C. acetobutylicum *ATCC 824. Cells were plated on CGM agar containing 50 μg/mL erythromycin and incubated at 37°C for about 2-3 days. The positive transformants containing the inserted intron were identified by colony PCR, using primers CAC2613-ID-fw and CAC2613-ID-rev (Additional file 10).

### Fermentation conditions

Solvent production and sugar utilization of *C. acetobutylicum *ATCC 824 and *C. acetobutylicum *EA 2018 were determined when the cultures were grown on P2 medium [53]. 6% Glucose or xylose contained P2 solution I (840 ml) and KH_2_PO_4 _(0.5 g/L), K_2_HPO_4 _(0.5 g/L), CH_3_COONH_4 _(2.2 g/L) contained P2 solution II (100 ml) were boiled for 20 min, then cooled by flushing O_2_-free N_2 _gas, and autoclaved at 121°C for 15 min separately. After autoclaving, 100 ml P2 solution II, 10 ml filter-sterilized P2 medium stock solution III (MgSO_4_·7H_2_O, 20 g/L; MnSO_4_·H_2_O, 1 g/L; FeSO_4_·7H_2_O, 1 g/L; NaCl, 1 g/L) and 1 ml solution IV (Para-amino-benzoicacid, 0.1 g/L; thiamin, 0.1 g/L; biotin, 0.001 g/L) were added into P2 solution I. The stock solutions were filter sterilized through a 0.2 μm pore-size filter. An inoculum of 5% from a CGM grown culture was typically used. Batch fermentation was carried out in 250 ml sealed bottle with 100 ml medium. 1 ml samples were taken every 12 h and analyzed for solvent and sugar. Accurate pH-controlled fermentations were carried out in BioFlo 110 bioreactors with 1.5 L working volume (New Brunswick Scientific, Edison, NJ). The pH control was achieved by using 9% (*w/v*) aqueous ammonia. Anaerobic conditions of fermentors were maintained through aeration of filtered nitrogen.

### Analytical methods

The surface morphology of *C. acetobutylicum *EA 2018 was studied using a JSM-6360 scanning electron microscope (JEOL Co. Ltd. Japan). Spore formation analysis was performed by growing the cells in P2 medium. After 48 h fermentation, cells were collected by centrifuging at 10,000 × g for 5 min, and stained with crystal violet for imaging analysis using a U-CTR30-2 microscope (Olympus Optical Co. Ltd. Japan). Butanol, acetone, ethanol, acetic acid and butyric acid were determined using a gas chromatograph (7890A, Agilent, Wilmington, DE, USA) equipped with a capillary column (Alltech EC™-WAX) and a flame ionization detector. The analysis was carried out under the following conditions: oven temperature, programmed from 80 to 140°C at a rate of 25°C/min; injector temperature, 200°C; detector temperature, 200°C; nitrogen (carrier gas) flow rate, 13 ml/min; hydrogen flow rate, 20 ml/min; air flow rate, 140 ml/min. Total solvent was defined as the sum of ABE. Isobutylalcohol and isobutyric acid were used as the internal standards for ABE and acid determination, respectively. Glucose and xylose were determined using a HPLC system (Model 1200, Agilent) equipped with a sugar-pak I column (Waters) and a refractive index detector. The analysis was carried out with water as mobile phase at a rate of 0.6 ml/min, and the column temperature was set up at 70°C. The composition of the gas produced (mainly H_2 _and CO_2_) was measured using the method described previously [54].

## Abbreviations

ABE: Acetone-Butanol-Ethanol; ATCC: American Type Culture Collection; CCTCC: China Center for Type Culture Collection; ORF: Open Reading Frames; NTG: *N*-methyl-*N*-nitro-*N*-nitrosoguanidine; SNVs: Single Nucleotide Variations; RBS: Ribosome Binding Site; CcpA: Catabolite Control Protein A; CGM: Clostridia Growth Medium.

## Authors' contributions

SYH, YLY, GPZ, SY, WHJ conceived of the study. SYH performed the genome sequencing, microarray samples preparation and gene disruption. SYH and YG performed the strain fermentation and analysis. JBZ performed the spore formation analysis. SYH, HJZ and SYW performed the genome annotation and analysis work. SYH and WWZ drafted the manuscript. All authors contributed to and approved the final manuscript.

## Supplementary Material

Additional file 1 **Genome finishing procedures of EA 2018**. The file describes the genome finishing process including gap closure, weak regions and homopolymer sites verification. Click here for file

Additional file 2 **Weak region verification primers**. The file lists the primers used in genome weak region verification procedure. Click here for file

Additional file 3 **Variation places between EA 2018 and ATCC 824 verification primers**. This file lists the verification primers for all of the indels sites and SNVs sites found between EA 2018 and ATCC 824. Click here for file

Additional file 4 **Deletion and insertion sites**. The file lists a total of 46 deletion sites and 26 insertion sites between EA 2018 and ATCC 824. Click here for file

Additional file 5 **SNV sites**. The file lists 440 single nucleotide variations between EA 2018 and ATCC 824. Click here for file

Additional file 6 **Protein variations**. The file shows the protein sequence variations affected by deletion, insertion or SNVs between EA 2018 and ATCC 824. Click here for file

Additional file 7 **Predicted promoter variations**. The file shows the predicted promoter variations between EA 2018 and ATCC 824. Click here for file

Additional file 8 **DNA array data**. The file lists the complete set of DNA array data of EA 2018 versus ATCC 824. Click here for file

Additional file 9 **The scheme of targeted gene disruption using group II intron**. The file displays the gene disruption procedure using group II intron. Click here for file

Additional file 10 **Targeted gene disruption and verification primers**. The file lists the primers for retargeting the RNA portion of the intron for *C. acetobutylicum *CAC2613 gene disruption and for identifying the insertion mutant.. Click here for file
