# Supplementary material for: Comparative genomic and transcriptomic analysis revealed genetic characteristics related to solvent formation and xylose utilization in Clostridium acetobutylicum EA 2018
Source: BMC Genomics. 2011 Feb 2;12:93. doi: 10.1186/1471-2164-12-93 (PMC3044671; doi:10.1186/1471-2164-12-93)
Supplement: Additional file 1 — Genome finishing procedures of EA 2018. The file describes the genome finishing process including gap closure, weak regions and homopolymer sites verification. [file 1471-2164-12-93-S1.PDF]

Additional file 1. Genome finishing procedures of EA 2018

Using Roche 454 pyrosequencing, a total of 261640 reads were obtained with the coverage of 15.1, which were assembled into 157 large contigs. After comparison with the genome of type strain ATCC 824, 165 gaps were found in draft seqence of EA 2018 genome, and the size distribution of gaps were visualized (Figure S1). The size of largest gap was 1629 bp. After gap closing, the error rate of EA 2018 genome sequence and megaplasmid were 2.2 bp/10 kb and 1.1 bp/10kb respectively. Then the weak regions (low value sites) were verified by PCR sequencing, and the primers used were listed in additional file 2. Meanwhile comparison with ATCC 824 revealed 447 indels and 487 SNVs, all of these indels sites and SNVs sites were also verified by PCR sequencing, the primers used were listed in additional file 3. The results showed that homopolymer sites (more than 4 consecutive same bases, *eg: AAAa*) caused 378 indels between EA 2018 and ATCC 824, and 355 of them were sequencing errors verified by PCR sequencing. The 454 low value sequence site before and after PCR re-sequencing were shown in Figure S2. The final error rate of EA 2018 genome and plasmid was 0.81 bp/10 kb and 0.74 bp/10 kb, respectively.

Figure legends

Figure S1. Gap sizes distribution of *C. acetobutylicum* EA 2018 whole genome after Roche 454 pyrosequencing and assemble.

Figure S2. Low value bases distribution of *C. acetobutylicum* EA 2018 whole genome before and after PCR verification. H, means high value bases; L, means low value bases. The value of base was analyzed by Consed.

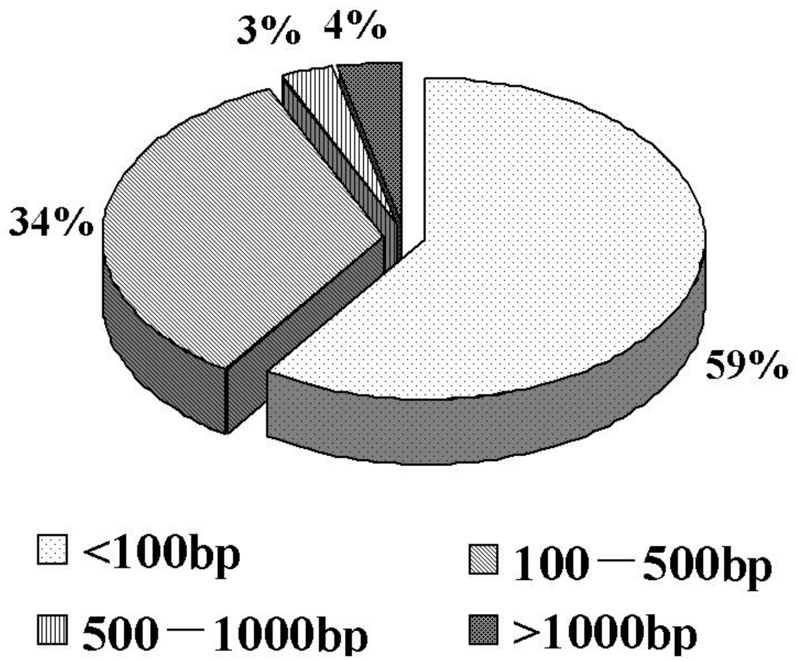

Figure S1

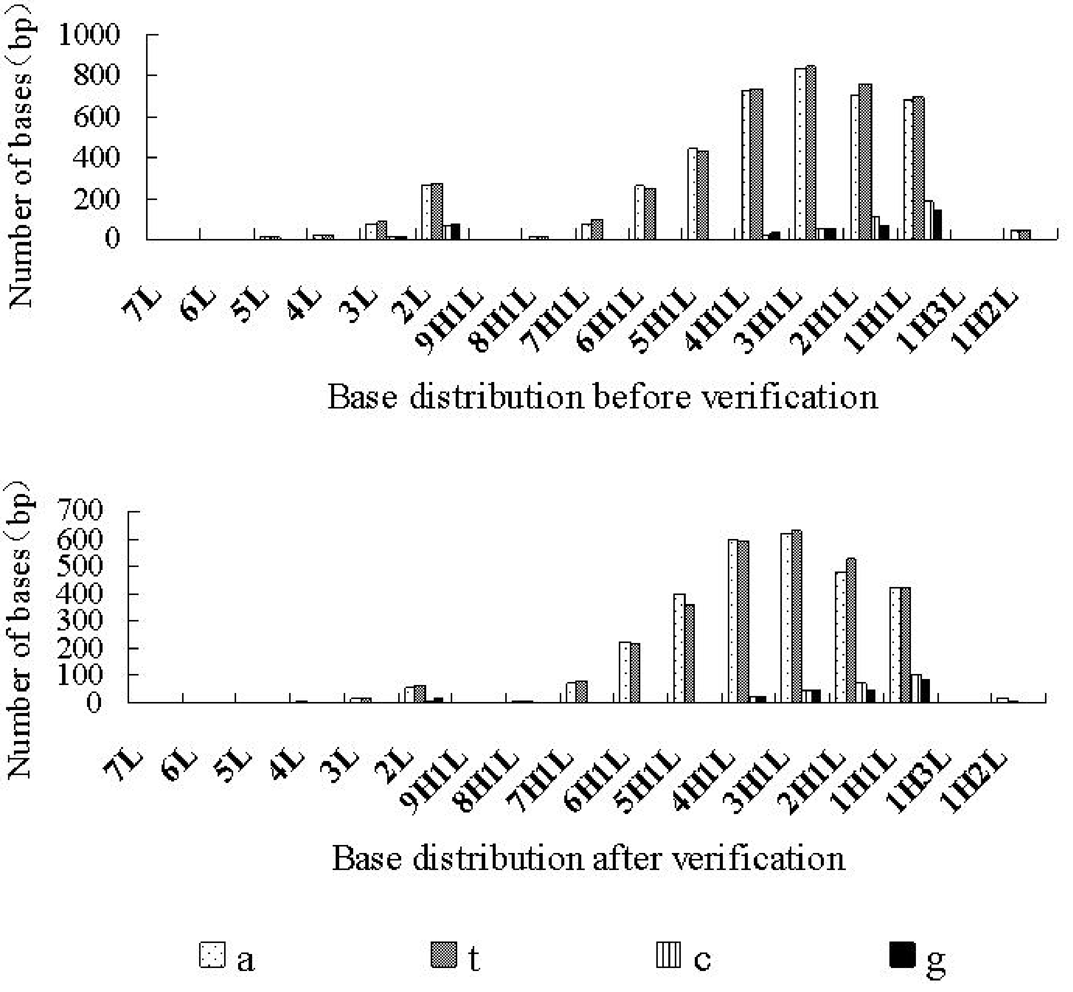
Number of bases（bp）
1000
800
600
400
200
0
7L
6L
5L
4L
3L
2L
9H1L
8H1L
7H1L
6H1L
5H1L
4H1L
3H1L
2H1L
1H1L
1H3L
1H2L
Base distribution before verification
Number of bases（bp）
700
600
500
400
300
200
100
0
7L
6L
5L
4L
3L
2L
9H1L
8H1L
7H1L
6H1L
5H1L
4H1L
3H1L
2H1L
1H1L
1H3L
1H2L
Base distribution after verification
a
t
c
g

Figure S2
