# Supplementary material for: Comparative genomic and transcriptomic analysis revealed genetic characteristics related to solvent formation and xylose utilization in Clostridium acetobutylicum EA 2018
Source: BMC Genomics. 2011 Feb 2;12:93. doi: 10.1186/1471-2164-12-93 (PMC3044671; doi:10.1186/1471-2164-12-93)
Supplement: Additional file 2 — Weak region verification primers. The file lists the primers used in genome weak region verification procedure. [file 1471-2164-12-93-S2.PDF]

Additional file 2. Weak region verification primers

Name	Sequence	Location in genome *	Name	Sequence	Location in genome *
CEA_WeakR1-p1	CTCTTATTCTCCACCAT	14550	CEA_WeakR269-p1	TTGTTCCTGCTGCTGTT	2033019
CEA_WeakR1-p2	TACAACTCCTCCAAACT	15469	CEA_WeakR269-p2	CTTGAGGCAATGGGTAT	2034296
CEA_WeakR2-p1	TCCCTGAATTGCCTCAA	130554	CEA_WeakR270-p1	CCCCTATTATGAAGTAT	2048045
CEA_WeakR2-p2	ATTCCATTGCCTGCTGA	131308	CEA_WeakR270-p2	TATTGAAAGGTGGAGAT	2049452
CEA_WeakR3-p1	CGGTTCCTATGGTGATT	189882	CEA_WeakR271-p1	TGATAATACAAGGTGCTAG	2051966
CEA_WeakR3-p2	TTGCGGCACAACTATCT	190601	CEA_WeakR271-p2	CAGAATCTACGGCTAAT	2053144
CEA_WeakR4-p1	TATTCCTGGCATAGTTG	330579	CEA_WeakR272-p1	TTGCCATCTACTCACCA	2054423
CEA_WeakR4-p2	GTACCGTCATTATCGTC	331377	CEA_WeakR272-p2	AAGCCAAGCATCTTACA	2055818
CEA_WeakR5-p1	CTAGTGCAGCGAACAAT	343085	CEA_WeakR273-p1	TTATTCCCTTTGTGATG	2055744
CEA_WeakR5-p2	CGTCCCATAAGACAGAGT	343085	CEA_WeakR273-p2	GGCGGACTAGGTATAGATT	2056590
CEA_WeakR6-p1	GAGTGAGCGTTCTTTAC	348443	CEA_WeakR274-p1	TTGCTGCCTCATCATTT	2071186
CEA_WeakR6-p2	TGAGCTGGTTTCCCTAT	349217	CEA_WeakR274-p2	GCTCAAGCGGTTGTTAT	2071407
CEA_WeakR7-p1	TGATTTTGAGCACCTCC	368009	CEA_WeakR275-p1	TCTTGTGGCAACCTACT	2071251
CEA_WeakR7-p2	AGCTTGTTTCCACGATA	368735	CEA_WeakR275-p2	ACTTCCTATTCGTGGAG	2072618
CEA_WeakR8-p1	ATAGCGGTTATTGTTGG	425685	CEA_WeakR276-p1	CCTTTACTGCCTTTCCA	2074301
CEA_WeakR8-p2	TACTCCTCATGCTCCTT	426273	CEA_WeakR276-p2	AACATTTGCGAGGATGA	2075146
CEA_WeakR9-p1	TTCTCAGATGGGAGTGA	478624	CEA_WeakR277-p1	CCCAAACATGGATTTAC	2117888
CEA_WeakR9-p2	CTGTTTGATACATTGCTTT	479613	CEA_WeakR277-p2	GGAAGGGTTTACAAATA	2118466
CEA_WeakR10-p1	CTGACTTTGTTATTCCCTC	484380	CEA_WeakR278-p1	AGGTAAAGTTCGAGGCT	2118061
CEA_WeakR10-p2	CTCTTTACACTGCCTCC	485120	CEA_WeakR278-p2	TGGCAGGGCTAGGATAT	2119024
CEA_WeakR11-p1	GTTAAGCAGGCAAAACC	487536	CEA_WeakR279-p1	TCATATTATTCCCTTCC	2125549
CEA_WeakR11-p2	TGAAGCATCTATTATGTGA	488117	CEA_WeakR279-p2	GAATTAGATTCATTTGGTC	2126556
CEA_WeakR12-p1	CAAAGGGGAGAAAATCT	497519	CEA_WeakR280-p1	TAGCAATGTCGTCATCA	2126102
CEA_WeakR12-p2	CAAACAAGGAAAACGAA	498325	CEA_WeakR280-p2	TGGGGTAGTTCAAAGTC	2126943
CEA_WeakR13-p1	GCAGCAGAAATGAGAAA	500215	CEA_WeakR281-p1	GCAATTTCTAAGCCTCC	2127548
CEA_WeakR13-p2	AATGCAGCAATAGAAGC	500955	CEA_WeakR281-p2	TTCCGTTTACATCAGTT	2128935
CEA_WeakR14-p1	CCTCCCATAGGCATTTA	506339	CEA_WeakR282-p1	AATGGGTTACCTTTGGG	2132090
CEA_WeakR14-p2	AAAGAATCCGCAGTAAG	507158	CEA_WeakR282-p2	GAGGCATTAGGAAGAGTTT	2133461
CEA_WeakR15-p1	TGGGCATTATCTCACTT	516449	CEA_WeakR283-p1	TTTTACTGTCAACGGCTAT	2133379
CEA_WeakR15-p2	ATCTTCAATGGCTGGTA	517163	CEA_WeakR283-p2	AGCATCGGGAGATAACG	2134438
CEA_WeakR16-p1	AAGAGGGTAGCAGATGA	542367	CEA_WeakR284-p1	TATTACAATCCGCTCTA	2134714
CEA_WeakR16-p2	GTTGATTGCCTTTGTAT	543245	CEA_WeakR284-p2	AAGTTGCCTTTGAAGTT	2136210
CEA_WeakR17-p1	AATTGTCGGAGGATTTT	544344	CEA_WeakR285-p1	CCTCCATAGGTAGGTGT	2136781
CEA_WeakR17-p2	TGACCACTCGCTTCTTT	545155	CEA_WeakR285-p2	CTGTGATGGCATTATTT	2137797
CEA_WeakR18-p1	ATTTAATGGTGGTCATG	619924	CEA_WeakR286-p1	CGGAATAACCTTTGTCA	2142702
CEA_WeakR18-p2	GAAACCGATAATCCTAT	620537	CEA_WeakR286-p2	GTCCAAGTTTTCTTCTGC	2146395
CEA_WeakR19-p1	TAAAGGATGCTCAAGGA	625543	CEA_WeakR287-p1	CCTGATTCTTCCCCACA	2144934
CEA_WeakR19-p2	TTTAATTGGCGTACAAA	626011	CEA_WeakR287-p2	TAAGTGATCCACCGAGT	2147991
CEA_WeakR20-p1	AGTTCAGAATAAGGGATG	629225	CEA_WeakR288-p1	TTTATGCAAAGACAGAG	2146650
CEA_WeakR20-p2	GTTTCTACCGATAAAGC	629946	CEA_WeakR288-p2	GGATATTGGCTAGAAGG	2149009

CEA_WeakR21-p1	AAGAAATGGCTGGAATG	659828	CEA_WeakR289-p1	TTTTATTACTCGGTGGA	2147984
CEA_WeakR21-p2	ATGCTTAGTTGGGAATA	660567	CEA_WeakR289-p2	GATACTGCGAGATGTGA	2150363
CEA_WeakR22-p1	AAGGCGGACTTGAACTT	660603	CEA_WeakR290-p1	ATTTAATGGTGCTGTTG	2148972
CEA_WeakR22-p2	GGTGGTCCTTCCGTAGA	661236	CEA_WeakR290-p2	TTCTGCTTTATGTGCTT	2157099
CEA_WeakR23-p1	GCTGCTGACGAAGAGTA	667695	CEA_WeakR291-p1	TAGGTCGTAATATCAATG	2155997
CEA_WeakR23-p2	AAGTGCTGGTAAGGTTG	668523	CEA_WeakR291-p2	CAAGAAATTGAGGGTAT	2159770
CEA_WeakR24-p1	TTGTGGAAGGGTAAAGT	675496	CEA_WeakR292-p1	TCTAGGCTTTCATTCAG	2158856
CEA_WeakR24-p2	GCAAATAGAAGTGGAGC	676391	CEA_WeakR292-p2	ATTGAGTTCCAGTTTGA	2160606
CEA_WeakR25-p1	AGCAGGCATTTTCGTAC	678863	CEA_WeakR293-p1	TAAGCACCCCATTAGAT	2159508
CEA_WeakR25-p2	CTCTGAGCCATTTCGTC	679553	CEA_WeakR293-p2	ATACGGACTAATGCTTC	2161822
CEA_WeakR26-p1	TGGCAAACATACTGGAA	690934	CEA_WeakR294-p1	GGGTGGATAAACTCCTA	2160412
CEA_WeakR26-p2	ATTCTTCGGTTCTTTCG	691466	CEA_WeakR294-p2	CAGCCAATAACAAGAATAG	2163628
CEA_WeakR27-p1	AGAATAGGTGCTGTGAT	691434	CEA_WeakR295-p1	AGTAGCCGGTGTAAGAA	2162112
CEA_WeakR27-p2	CATTTGGTCTTCCGTTA	692143	CEA_WeakR295-p2	TTATGGCTGCTACTTTA	2164527
CEA_WeakR28-p1	ACGATAAGTATTGGAGGTT	692562	CEA_WeakR296-p1	AGAGGTTCTCCGCTTAC	2163876
CEA_WeakR28-p2	TTGCATTTGAGGGATAG	693274	CEA_WeakR296-p2	TGTGAGGTGCGGATTTA	2170636
CEA_WeakR29-p1	AGAGCTATCCCTCAAAT	693270	CEA_WeakR297-p1	AAAGTAGCAGCCATAAC	2164528
CEA_WeakR29-p2	ATACCCGAGTATGTTTT	694137	CEA_WeakR297-p2	GTAAAATGCAAGAGTGA	2173696
CEA_WeakR30-p1	AAACTACTCATTGCTCCTT	714993	CEA_WeakR298-p1	AACTATTAGCCCGTCCAT	2170030
CEA_WeakR30-p2	TTCAACCTTCCTATCCC	715879	CEA_WeakR298-p2	AATAAGCGGCAGGATAG	2174817
CEA_WeakR31-p1	ACATGCAGCAATACTCAA	716995	CEA_WeakR299-p1	GAGAACAACAAGGTCAG	2172512
CEA_WeakR31-p2	AAGCAAGAAACCGAAAT	717895	CEA_WeakR299-p2	TTCGCCAATTAAGGGAC	2177674
CEA_WeakR32-p1	GGCATTGGTGTATAGTCAT	725352	CEA_WeakR300-p1	TAGCTGCTGGACTTTGC	2173871
CEA_WeakR32-p2	AAGTTTAGGCTGCTTTT	726211	CEA_WeakR300-p2	GAAGCCTTTGAGAAACTAA	2183476
CEA_WeakR33-p1	TTTTACATAAGGGAAGG	731119	CEA_WeakR301-p1	CATTAGCTGAACCCGATAT	2176406
CEA_WeakR33-p2	ATGAAATCTAGCCAAAG	731948	CEA_WeakR301-p2	AAATAGATGGAGTGGGT	2186834
CEA_WeakR34-p1	GGCTTACTTTATCCTTA	733997	CEA_WeakR302-p1	ATTCACCTTGCCATCCT	2182646
CEA_WeakR34-p2	GACCCTAAGGATAAAGTAA	734000	CEA_WeakR302-p2	AGCAGTTCCAATACTCA	2188957
CEA_WeakR35-p1	TGCAAAAGCTCTGATAA	742170	CEA_WeakR303-p1	ACAAAGTGCGTCCTTAA	2186035
CEA_WeakR35-p2	GTTACCTGATTATCCAA	734935	CEA_WeakR303-p2	TGCGAACCAGGAGTAAA	2191279
CEA_WeakR36-p1	AGGTCAAGTGATCCAAAT	772208	CEA_WeakR304-p1	TTTACGAACTCAAGCAC	2188247
CEA_WeakR36-p2	CCTTGTAAGCCTTCCTC	772901	CEA_WeakR304-p2	TACAGAGCAGGCAAAGC	2192650
CEA_WeakR37-p1	GTTAAAGCCAAAGACGG	776232	CEA_WeakR305-p1	TCAACAAGCGGTGTATG	2189863
CEA_WeakR37-p2	AATCAAGTTTCCCACCA	776801	CEA_WeakR305-p2	AAAGGGCAAGATGTATT	2195404
CEA_WeakR38-p1	GCTTATAGAAGCCAAGA	777020	CEA_WeakR306-p1	GTTACAGCACCAGTTAT	2191536
CEA_WeakR38-p2	AGTTATACGAAAGGGTG	777875	CEA_WeakR306-p2	GTGCATAGGCTTTGATA	2204376
CEA_WeakR39-p1	AGATGGGTTTTGGGTAT	778720	CEA_WeakR307-p1	GCCACTAACTTTCTCCA	2194871
CEA_WeakR39-p2	CATTATTTATGGAATGCTC	779608	CEA_WeakR307-p2	CGAAATAGTTACCCACA	2218729
CEA_WeakR40-p1	AAATAGGCTATGAGGAA	788282	CEA_WeakR308-p1	AAACTTAACTCGCTTGA	2203676
CEA_WeakR40-p2	GTAATATCTGCTGACCC	789049	CEA_WeakR308-p2	AGATGCGGATGTTGACG	2220305
CEA_WeakR41-p1	AAAGTGGGATGGTCTTG	796773	CEA_WeakR309-p1	GTTATAGCAACCACCTT	2217942
CEA_WeakR41-p2	CAGCGTATGATGATGTCTC	797619	CEA_WeakR309-p2	GAGCGACTTAAAGAGTA	2223478
CEA_WeakR42-p1	TAAGTAGGGCGATAAGA	819162	CEA_WeakR310-p1	TAACAGCCATTGCCTTTC	2219461
CEA_WeakR42-p2	AATTGGCAGCTAAAGTA	820170	CEA_WeakR310-p2	CAGGAGTGCTTATTGGA	2223771

CEA_WeakR43-p1	TAATAATCGTCGCAAGT	832326	CEA_WeakR311-p1	TTACCACTTATGGCTAG	2221966
CEA_WeakR43-p2	TAATACGGCTAAACCAC	833177	CEA_WeakR311-p2	TGGATGAACAATGGGAT	2225570
CEA_WeakR44-p1	GATGCTTCCGCAATGAA	839192	CEA_WeakR312-p1	GTCAAAGGAGCAAGCAA	2224559
CEA_WeakR44-p2	TAAACTCGCTCGCTCAC	839918	CEA_WeakR312-p2	TACTCCACCATCATCGA	2228317
CEA_WeakR45-p1	GGCTATGGCAGAACCTA	839862	CEA_WeakR313-p1	TCTTTTCCTGTAACATAAC	2227536
CEA_WeakR45-p2	TGATCCCATGAACAAGC	840537	CEA_WeakR313-p2	GTAGCAGCAGTAGAAGA	2229055
CEA_WeakR46-p1	CCGACTATCCAGTCCCT	843657	CEA_WeakR314-p1	AAAGCCAGATAGAAGTA	2228105
CEA_WeakR46-p2	GCAATACATCCTCCTAAAA	844596	CEA_WeakR314-p2	AGAAGTCAAAGGTGGTA	2232114
CEA_WeakR47-p1	ATTTTCCAGTGGTTTTG	846897	CEA_WeakR315-p1	TTGTTGGTGCTGTAAGT	2230636
CEA_WeakR47-p2	GTATTTCCGTATTAGGT	847725	CEA_WeakR315-p2	AAGTCCAAGCCCTGAAA	2234595
CEA_WeakR48-p1	CGGTTGATCCTTCATAT	849214	CEA_WeakR316-p1	AACCACTTCCTCCATCA	2233764
CEA_WeakR48-p2	CCAATTCTCCTCCACTT	849948	CEA_WeakR316-p2	ATTTAATTTGCGGTGAG	2235242
CEA_WeakR49-p1	TCAAATGCAAATGTTTCG	852298	CEA_WeakR317-p1	ATACCCTACTAAATCTTCG	2235017
CEA_WeakR49-p2	CCGCCTAAGGTAATCTGTG	853070	CEA_WeakR317-p2	TCTGACAACGCCTGAAC	2236207
CEA_WeakR50-p1	AGAAGTTTGAGGACCAT	868063	CEA_WeakR318-p1	GGGTTGCTTCTTCAGTT	2240955
CEA_WeakR50-p2	TGTTTCCGTAGTATCCA	868946	CEA_WeakR318-p2	AGATAATCAGTCAGGGA	2242312
CEA_WeakR51-p1	CCCAGTTGTTTGTTATT	870324	CEA_WeakR319-p1	GCTGCTTTCTTACTTCT	2243339
CEA_WeakR51-p2	CGCCCTTAGCACCTATT	871328	CEA_WeakR319-p2	TTAGGGTATTCTATGGT	2244862
CEA_WeakR52-p1	GTTATCGCCTTTCTTTG	874241	CEA_WeakR320-p1	TGCCTCCGATACATTAC	2248736
CEA_WeakR52-p2	GTTTGCTCCATCTACCT	875165	CEA_WeakR320-p2	GAACCACCAGCAACTAA	2248948
CEA_WeakR53-p1	CCCATTACCAGGAGGAA	893458	CEA_WeakR321-p1	CCTCGTTAAAGCCTACA	2249585
CEA_WeakR53-p2	ACCACTGCTGCCAATAT	894174	CEA_WeakR321-p2	ATTACCCTTGATGATGC	2250802
CEA_WeakR54-p1	ATCTACTCTTCTTTTGGTT	906466	CEA_WeakR322-p1	TAGAAGCCAAAAGAGGT	2256163
CEA_WeakR54-p2	AATTACATCCCCTACGT	907496	CEA_WeakR322-p2	TACAGAGGGTGGAGCAA	2257163
CEA_WeakR55-p1	GTCGCCACTTAGAGGAT	941469	CEA_WeakR323-p1	ACAAACTAAGTATTCCAT	2258021
CEA_WeakR55-p2	GTGCTGCATTATTTACT	933234	CEA_WeakR323-p2	GGTATCTTTATCAGCAT	2259322
CEA_WeakR56-p1	GTGATGACTACCTTTGG	965754	CEA_WeakR324-p1	CCACTGCTGTTTTAGAT	2263939
CEA_WeakR56-p2	AGAGTACGCTTCTTCTA	966503	CEA_WeakR324-p2	CTAGCGTGCAAGGTAGA	2265269
CEA_WeakR57-p1	CAGTTACCAAAACCATC	973235	CEA_WeakR325-p1	ACCATTCCCATGTTGTT	2265403
CEA_WeakR57-p2	ATTCTTTATCCCCTTCT	973939	CEA_WeakR325-p2	TGTAGGAGTTAAGGAGC	2265942
CEA_WeakR58-p1	AAAGCTAGTTTGGAGGAA	991264	CEA_WeakR326-p1	CGCCTATTACTATGTGG	2269561
CEA_WeakR58-p2	AATCACGGACAAGCAAC	992088	CEA_WeakR326-p2	TGACGACTATTGAGGGA	2270747
CEA_WeakR59-p1	ATCGGAGGAAATATGGA	993038	CEA_WeakR327-p1	GGCAAAGCTAATGAAGA	2270402
CEA_WeakR59-p2	CCTTGCACTATTAGACA	993957	CEA_WeakR327-p2	GTAAGCCAGATGGAATG	2271436
CEA_WeakR60-p1	AGGAGCTTGGAGGATAT	994110	CEA_WeakR328-p1	TGCGTAAGTCTATCTGC	2271221
CEA_WeakR60-p2	TTAAGGCTAATTGTTGC	995199	CEA_WeakR328-p2	ATCCTAGAAGCCCGTAT	2272872
CEA_WeakR61-p1	GGAAGCAGCAACTATTA	996372	CEA_WeakR329-p1	CATTTAAGTTTCCCAGTG	2272676
CEA_WeakR61-p2	CTTACATCATCCGTTTT	997300	CEA_WeakR329-p2	GGATAATAAGACGTGCC	2273232
CEA_WeakR62-p1	AAGATCAGCACCAGAAA	998791	CEA_WeakR330-p1	AAATAAGACCCATCCTG	2273287
CEA_WeakR62-p2	CAGCCTATACTTGTCCC	999478	CEA_WeakR330-p2	GGTGAAAGCGTATAAAGTA	2274006
CEA_WeakR63-p1	GGCTGTCTCAAGTTCTG	1000991	CEA_WeakR331-p1	CTGGATACCATGTGCTTTT	2273675
CEA_WeakR63-p2	ATCCTTATACGTCCATA	1001920	CEA_WeakR331-p2	ACGCCATAGTGTATTCATC	2274726
CEA_WeakR64-p1	TTCGGAGCAGACATAAC	1009850	CEA_WeakR332-p1	CGGAAAGAAGGTAATAGA	2274847
CEA_WeakR64-p2	TCCCACCAAGTGAGTAA	1010724	CEA_WeakR332-p2	TGTCGTATGCCAATGTG	2276061

CEA_WeakR65-p1	TGAGGCTTTGGGAATAA	1013175	CEA_WeakR333-p1	ACGGAAAGGAAGTAATA	2276156
CEA_WeakR65-p2	CATCCACAGGGTTTAGC	1013898	CEA_WeakR333-p2	TTTGTAGCGTAGGTGTT	2277278
CEA_WeakR66-p1	GCCAGTTCAGTTAAGGC	1014028	CEA_WeakR334-p1	TCCCATGCCATCAATAA	2277077
CEA_WeakR66-p2	CAATTGTCACCAGACCC	1015137	CEA_WeakR334-p2	TGGAGCCAGAAGAGTTT	2278353
CEA_WeakR67-p1	AAATATGTAGCTCCTGG	1015895	CEA_WeakR335-p1	GCTAAGGTAAGGGTTCT	2277983
CEA_WeakR67-p2	GACTTCTTATGCCTTTC	1016996	CEA_WeakR335-p2	GACTTCCAGTGATTTCG	2279367
CEA_WeakR68-p1	GGAGGAGGGATAACAAT	1017161	CEA_WeakR336-p1	ATTTCTACCGCTCTATC	2279019
CEA_WeakR68-p2	CTGAAGGTCGTCCAAAT	1017959	CEA_WeakR336-p2	TCAACTTATTAGGCTTG	2280380
CEA_WeakR69-p1	GGCCCTCAGTATCTCAA	1020345	CEA_WeakR337-p1	ATAGCATCTGTCCAACG	2280168
CEA_WeakR69-p2	TGCAGTAATTCTCAACG	1021148	CEA_WeakR337-p2	GAAACAAAGGATGAAAAGT	2281274
CEA_WeakR70-p1	TTCCGTCTGAGGTTTCT	1021196	CEA_WeakR338-p1	CTCCATCATAGGGTTCA	2280791
CEA_WeakR70-p2	AGTGTCTTCCAGCCTTT	1022017	CEA_WeakR338-p2	AAAGCAGGAGGAGATTA	2281471
CEA_WeakR71-p1	AAGTCAACTTCCACATG	1023423	CEA_WeakR339-p1	ATTCTTTACCGTTCTAC	2281662
CEA_WeakR71-p2	AGCAACTTTACCACCTA	1024123	CEA_WeakR339-p2	ATAAGGGTTACAGGAGA	2282779
CEA_WeakR72-p1	TATAAGGGATGGCGAATT	1024277	CEA_WeakR340-p1	TAACCCTACCTTCCTTG	2283240
CEA_WeakR72-p2	CCCTGACCGTCAAACAC	1025123	CEA_WeakR340-p2	CTTAGAAATGCCTCTTG	2284512
CEA_WeakR73-p1	ATCCTGATTTTGAAGCATA	1040279	CEA_WeakR341-p1	TTTTCATACCCGCTTTC	2284259
CEA_WeakR73-p2	TATCCTCCGCTTGTGAA	1041212	CEA_WeakR341-p2	TTTGGATTAGGTCTTGG	2285606
CEA_WeakR74-p1	AATGTCATCCAGCTCCTG	1051367	CEA_WeakR342-p1	GGCTTTAATGTTCTTCC	2289039
CEA_WeakR74-p2	CCATCCCGTCTTGTTGT	1052479	CEA_WeakR342-p2	GAACTGTTCAGCCTATT	2290283
CEA_WeakR75-p1	ATCCTCCAGAGCATCCA	1052140	CEA_WeakR343-p1	ATGGAATACCAAAACTC	2290102
CEA_WeakR75-p2	CACGACCATTTATTACCC	1053013	CEA_WeakR343-p2	ACCGATGAAGATAAGAA	2291474
CEA_WeakR76-p1	CTGTGGAGTCGTGGGTT	1065985	CEA_WeakR344-p1	TACCCCGCAAATACCAT	2291172
CEA_WeakR76-p2	TTGGCTTGATTTCACCT	1066865	CEA_WeakR344-p2	GGGAAGCGAGGAAAGAA	2292322
CEA_WeakR77-p1	TCCAGGTGAAATCAAGC	1066862	CEA_WeakR345-p1	TTTCCAAAATCCCCATA	2292192
CEA_WeakR77-p2	TCATAGCCAAGCAAAGC	1067729	CEA_WeakR345-p2	TTGTGCAGGTAGTGCTG	2293243
CEA_WeakR78-p1	TACGAAGCCTTAGAGTG	1068063	CEA_WeakR346-p1	TTCCCTGTTTCCACCAC	2293014
CEA_WeakR78-p2	TTTACAGCCGCTACATC	1069172	CEA_WeakR346-p2	TAGGAGGATAAAGATGAGC	2294273
CEA_WeakR79-p1	AAAGCAGTTGGGGAAAA	1069013	CEA_WeakR347-p1	TATCCACTGAAACCCAT	2294108
CEA_WeakR79-p2	GCTTTAATCGGGCGTAC	1070082	CEA_WeakR347-p2	CAGCAGGTGATACAAAT	2295015
CEA_WeakR80-p1	TGATAACATTGCCGCTAG	1070304	CEA_WeakR348-p1	TGGCTCCTCCCTCAGTT	2294845
CEA_WeakR80-p2	AGCTGCCATTCCAGAAG	1071139	CEA_WeakR348-p2	TACGCTTATGGTTCATTCA	2295561
CEA_WeakR81-p1	TAGGATAGATTGGGTAA	1072974	CEA_WeakR349-p1	GAAACTACCACCACAGG	2295467
CEA_WeakR81-p2	TCTTCTTCTTCGCATTT	1073906	CEA_WeakR349-p2	AGGCAGCAAACTCTATC	2296978
CEA_WeakR82-p1	ATACAAATAGAGGCAAGTC	1073701	CEA_WeakR350-p1	AGCTCCTATCTGGAATG	2296619
CEA_WeakR82-p2	CTAAGTTCGCCTACAAC	1074420	CEA_WeakR350-p2	AGGCTGGACAAATGAAA	2298043
CEA_WeakR83-p1	GGCCTCTTGAGGATGAA	1074172	CEA_WeakR351-p1	GTCTTCTAGCCATAGTTT	2297889
CEA_WeakR83-p2	AAACGATACCAACAGCA	1075376	CEA_WeakR351-p2	TTGTTGGTGTAGGAGGT	2298734
CEA_WeakR84-p1	TAAATTACTACACGGGTCT	1076630	CEA_WeakR352-p1	TCTATTTCCGTAACCAT	2304751
CEA_WeakR84-p2	GCTTCATACTTGCCCTA	1077586	CEA_WeakR352-p2	GACCTATGCAGCAGTCA	2305714
CEA_WeakR85-p1	GGATGTGGAAGGCTTGA	1081537	CEA_WeakR353-p1	GAGACCCTGTACCAAGA	2305503
CEA_WeakR85-p2	GTACCTGGATGCCCTGA	1082531	CEA_WeakR353-p2	AGGGCAAACTGAAATAA	2306716
CEA_WeakR86-p1	GTTGAAGATTGGGAATA	1090720	CEA_WeakR354-p1	GAGGAGCTAAAGTTTGA	2306941
CEA_WeakR86-p2	GCTGCACCTAGAAGACC	1091755	CEA_WeakR354-p2	AAGCAAGTAACGGTCTA	2308301

CEA_WeakR87-p1	ATTTCAGCCTGGCTTTC	1091443	CEA_WeakR355-p1	GTCCCATTGCAGAACAC	2308156
CEA_WeakR87-p2	CTGGCGGTTACACCTTA	1092420	CEA_WeakR355-p2	GGAGGCAGAAATAAAGAAA	2309310
CEA_WeakR88-p1	CATCTTCGGATACCTTT	1093478	CEA_WeakR356-p1	CTGCCTTAGTTCCTTCT	2309696
CEA_WeakR88-p2	TTCCTTTACTCGCTCAT	1094358	CEA_WeakR356-p2	TATGGGCTACAATACGA	2310930
CEA_WeakR89-p1	TCCGTGTCAAGGCTTAT	1098974	CEA_WeakR357-p1	CTGGCTCCTGCTCTAAT	2311027
CEA_WeakR89-p2	GATCCACCTGGCATTCT	1100140	CEA_WeakR357-p2	ATATGCCTGTTGGTTTT	2312354
CEA_WeakR90-p1	CATGCCATCTATTGTTT	1130703	CEA_WeakR358-p1	CGGATTCAACCTTCATA	2314751
CEA_WeakR90-p2	TAATCCCATAGGCTCAA	1131298	CEA_WeakR358-p2	AAAGCGTCGATAGCAGT	2315701
CEA_WeakR91-p1	TAAATAGCCACAGAAAC	1166891	CEA_WeakR359-p1	TTATCATCTCAGTTCCCTG	2316097
CEA_WeakR91-p2	CGAATACTCCTCCTAAA	1167628	CEA_WeakR359-p2	GCTTCTGCGGAGTTCTA	2317265
CEA_WeakR92-p1	CAGGCTTCTTGATTTCC	1178085	CEA_WeakR360-p1	ATATCAGATAGGGAATC	2317074
CEA_WeakR92-p2	CACCCATAGGTGCTTTT	1179245	CEA_WeakR360-p2	TACTCAATTAAGGGTAG	2318398
CEA_WeakR93-p1	TGTCGGTATTTGAAGTT	1185314	CEA_WeakR361-p1	ACTTGGCATGATACTACAT	2318188
CEA_WeakR93-p2	CATCTATTTGGGACTGC	1186589	CEA_WeakR361-p2	GTTTCTAACGCAACTGG	2319548
CEA_WeakR94-p1	TATCCATATTTTCCTGATG	1215977	CEA_WeakR362-p1	AGTCATTAAAGCTGGTC	2319934
CEA_WeakR94-p2	CAATCTTATTTCTTAGCCT	1216829	CEA_WeakR362-p2	ACTATGGTGCTGAAGAT	2321263
CEA_WeakR95-p1	CTTTGCCTGAACTTACA	1264006	CEA_WeakR363-p1	ATTTCTCCCATGTAGTGC	2321302
CEA_WeakR95-p2	AGATGTCTAGCACCCTC	1264749	CEA_WeakR363-p2	GGGACATAGAGCGAATA	2322465
CEA_WeakR96-p1	CCGTTATTAGATTCCAT	1289255	CEA_WeakR364-p1	GTAGGCGATGTATGAGA	2322602
CEA_WeakR96-p2	ATTTACTACTGCCACAT	1290312	CEA_WeakR364-p2	ATAGTAATGACGGGTGG	2324099
CEA_WeakR97-p1	CCTTTATCAGAAGGAGGTA	1290944	CEA_WeakR365-p1	ATATCGGGATAAAATGC	2324291
CEA_WeakR97-p2	TCAAGTGGATTGGCAGA	1291990	CEA_WeakR365-p2	TATGTGGAGCTGGAGTT	2325395
CEA_WeakR98-p1	TAACAGATAGTCCGCAAAA	1291926	CEA_WeakR366-p1	ACATATTTCTTCGCTATCC	2326520
CEA_WeakR98-p2	AAGCAGGTGATGATGAGAT	1292914	CEA_WeakR366-p2	AGCAGCTAAGGCACTAA	2327508
CEA_WeakR99-p1	TTCTAACAGCCTTTTCA	1305466	CEA_WeakR367-p1	TGCCATCTTACTTTCAT	2328121
CEA_WeakR99-p2	TTATGTATGGAGGGATT	1306166	CEA_WeakR367-p2	TAACAGTAGCGGACATA	2329596
CEA_WeakR100-p1	ATGGGTATTATCCCTAA	1329408	CEA_WeakR368-p1	TATCTCGTTTTCATCTC	2329542
CEA_WeakR100-p2	CCTTTACTCCTCCTTGA	1330241	CEA_WeakR368-p2	ATTCTATCGTCTTCGTC	2330621
CEA_WeakR101-p1	GAATCTTAGCGGATGTA	1346745	CEA_WeakR369-p1	AACCAGCGGTGGAGTGA	2330353
CEA_WeakR101-p2	AAAGTCTACTGGCACAA	1347511	CEA_WeakR369-p2	GGATGGAGGCTAGAGGC	2331368
CEA_WeakR102-p1	AGAAGAATTACGAAGTATG	1349663	CEA_WeakR370-p1	TTGGAAGGGATACAATT	2331085
CEA_WeakR102-p2	CTTTATCTGTTCCACCC	1350341	CEA_WeakR370-p2	GAAGGGCATAGGATACA	2332527
CEA_WeakR103-p1	GACTGTTGGGACTTATT	1357099	CEA_WeakR371-p1	TATGACTTATGGGGTAT	2333201
CEA_WeakR103-p2	AGTTAGTCTTGCCTTTC	1357881	CEA_WeakR371-p2	TATTGGCTTTAGGTGTA	2334637
CEA_WeakR104-p1	GAAAGTATAGCTTGTATG	1359286	CEA_WeakR372-p1	TTTTAATGCCTCAATAGC	2335009
CEA_WeakR104-p2	ATTTAAGTTCTCCTCGA	1360053	CEA_WeakR372-p2	TGTTGAAAGATCAAGGA	2336052
CEA_WeakR105-p1	CGCTCAAGGAGAAAAGT	1360238	CEA_WeakR373-p1	TTCCAAACAACCCAAAA	2337178
CEA_WeakR105-p2	CTTGCTGCTCACCCTCT	1360943	CEA_WeakR373-p2	GTCCTCCTGAAAGAAAAGT	2338083
CEA_WeakR106-p1	TGAACTATCCTCCATTT	1364588	CEA_WeakR374-p1	CCCAAGACTTACTAACA	2338907
CEA_WeakR106-p2	CTCCTGAACCACGACTA	1365361	CEA_WeakR374-p2	GTAGATACGGGAAGATA	2340259
CEA_WeakR107-p1	AATGACTTATAGCGTTTAG	1368888	CEA_WeakR375-p1	GCATGGCCTCTATGTTC	2340486
CEA_WeakR107-p2	TCAGTTTTCTGCTCCTC	1369703	CEA_WeakR375-p2	CATAAAGGGATTTGGGT	2342049
CEA_WeakR108-p1	ACCAGGAGCAGATCCAA	1426656	CEA_WeakR376-p1	CCTTGACTTTACCTTAA	2350966
CEA_WeakR108-p2	ACCGCATGTTTCACAGTTT	1427324	CEA_WeakR376-p2	GTTCCTTTCTTCTGATA	2351856

CEA_WeakR109-p1	GAAGTGCAGAACTTATT	1430936	CEA_WeakR377-p1	TGCAATCATGTGCTATA	2382843
CEA_WeakR109-p2	TAGCCATACTGATTACA	1431995	CEA_WeakR377-p2	AACAGTTGAAACGAGAA	2384101
CEA_WeakR110-p1	TGAATGGGTAAACTAAG	1441036	CEA_WeakR378-p1	TTCCTGCTTTATGCTCT	2388650
CEA_WeakR110-p2	ATGTATCCCTCTACAAT	1441935	CEA_WeakR378-p2	ATTGTCTTTCGGCTTCT	2389641
CEA_WeakR111-p1	AGCATATGATGTACGGA	1444950	CEA_WeakR379-p1	GAAGCCGAAAGACAATG	2389642
CEA_WeakR111-p2	CCATAAGTCCAACATTG	1445901	CEA_WeakR379-p2	GACAAGCGGTAAAGGTG	2390453
CEA_WeakR112-p1	AAGATGGGCATAAATAC	1446567	CEA_WeakR380-p1	TAAGACTCTGTTGGCTTGT	2391270
CEA_WeakR112-p2	ATTGTCTGGCTTACTCG	1447646	CEA_WeakR380-p2	AGGGACTGTCGGTAAAA	2392566
CEA_WeakR113-p1	ATTCAGAGTGCCGTAAA	1454160	CEA_WeakR381-p1	ATACTTGTCCGTTATTTTG	2394705
CEA_WeakR113-p2	TTGCTTCCACCATTTAC	1455234	CEA_WeakR381-p2	AGCAATCGCAGTTTTAC	2395850
CEA_WeakR114-p1	CATGTTATTGCTCCTTT	1457143	CEA_WeakR382-p1	CCACATGCAGGATCTAC	2412103
CEA_WeakR114-p2	TAGAACAATTCCCACTT	1458402	CEA_WeakR382-p2	AAATGAAGGCTATGGAA	2413411
CEA_WeakR115-p1	ACATAGCATCGTTATCA	1468759	CEA_WeakR383-p1	TTTCTTTCGTCATCCAC	2413608
CEA_WeakR115-p2	AAACTTACTTCCCTCAC	1469921	CEA_WeakR383-p2	GCTTGCCTTCATACAAT	2414629
CEA_WeakR116-p1	TCTCCAGTAAGAGGGTC	1471524	CEA_WeakR384-p1	ACTATTCCGACATCCTC	2414509
CEA_WeakR116-p2	AATCCGCAATTAGCACA	1472463	CEA_WeakR384-p2	ATCGCCTACAAACTTAA	2415871
CEA_WeakR117-p1	TCCTACTATACGAGCAA	1477497	CEA_WeakR385-p1	TGTCCATTTGCTCCTGT	2418272
CEA_WeakR117-p2	CAAGGCATAAACAGAAT	1478537	CEA_WeakR385-p2	CTGGCTCTGATTTGTTTC	2419750
CEA_WeakR118-p1	TTCAGGCGATTTAGTTG	1485630	CEA_WeakR386-p1	TAGTTGGTTCACCCGTTAT	2426390
CEA_WeakR118-p2	TCAATCCTTTCGGCAAT	1486403	CEA_WeakR386-p2	TTGGGTTTCAGCAGATAGA	2427322
CEA_WeakR119-p1	GGAACCCAACTAATCAA	1508232	CEA_WeakR387-p1	TACAATCTCCCAGAGGC	2437273
CEA_WeakR119-p2	TCCTCCTATCGCTCGTA	1509504	CEA_WeakR387-p2	ATCTGAAGAAGCCGAAT	2438111
CEA_WeakR120-p1	AGAGTAATAAGGAGGGTT	1510280	CEA_WeakR388-p1	GGTGAGAAACGGCAAGT	2470663
CEA_WeakR120-p2	ATACATAGGCTGCTGTT	1511208	CEA_WeakR388-p2	AATGGGAAGTGACGAAG	2471356
CEA_WeakR121-p1	CTTGTCTTTACCACCCC	1511007	CEA_WeakR389-p1	CTAGTTCAGTAAGCTCCTT	2481574
CEA_WeakR121-p2	ACTTCCTTTACTCCTCC	1512117	CEA_WeakR389-p2	ATACTTGGCATTGTTTC	2482604
CEA_WeakR122-p1	ACAAGGAGGAGTAAAGG	1512113	CEA_WeakR390-p1	TTCCTCGCAAGATTTAC	2484892
CEA_WeakR122-p2	TAACTGCTATTCCCGTA	1513517	CEA_WeakR390-p2	AAGCATCACAACCGTAT	2485526
CEA_WeakR123-p1	AGGGCTTGATGATAGCT	1513254	CEA_WeakR391-p1	CGTGGTCATAAAGAAGA	2492047
CEA_WeakR123-p2	AGTCACTTCCCTCGTAT	1514177	CEA_WeakR391-p2	GAAGCCATAGAGGAAAT	2493389
CEA_WeakR124-p1	GTATCCTGCTAGTGTTG	1514341	CEA_WeakR392-p1	TTATCCACGAGTTCCTT	2494486
CEA_WeakR124-p2	CTCTATGTCTGCTCCTG	1515485	CEA_WeakR392-p2	AGTTCAAATGGTGCTTT	2495960
CEA_WeakR125-p1	CAGAACCTGGAAGCAAG	1516046	CEA_WeakR393-p1	TTCCCTGAAAGCACCAT	2495937
CEA_WeakR125-p2	TATGGACAAACGGAAAA	1517161	CEA_WeakR393-p2	TTGAACGGCTTGACTTG	2497352
CEA_WeakR126-p1	TATGGCAGGTAACTCAG	1516897	CEA_WeakR394-p1	ATTATTTGAGGGAGGGC	2504289
CEA_WeakR126-p2	CTATAAGCCTTCCAAGC	1518066	CEA_WeakR394-p2	CATGGAGCAATTATGAAGA	2505539
CEA_WeakR127-p1	TTTTGGAACACCTGATT	1517916	CEA_WeakR395-p1	TTCAAGACAACGGTGCT	2510801
CEA_WeakR127-p2	AAGTCCCATTACTCCCT	1518793	CEA_WeakR395-p2	TGGGCAAATGCTAGAGG	2512081
CEA_WeakR128-p1	GGGAAATGGAGACTACGG	1518767	CEA_WeakR396-p1	CAATTCATGTATCCGTCAG	2518307
CEA_WeakR128-p2	TAAGCCGCCACATTCAG	1519524	CEA_WeakR396-p2	ATAGCAGGAGGCGTAAT	2519419
CEA_WeakR129-p1	AAGGACGACTACCATTT	1519234	CEA_WeakR397-p1	ATACCATCAAGCGTCTT	2524095
CEA_WeakR129-p2	ATTCCCTCACTTACAGC	1520237	CEA_WeakR397-p2	GACTTGGGCAAATAAAG	2525012
CEA_WeakR130-p1	ACCCTGGAAAGATTGAA	1519887	CEA_WeakR398-p1	TTCCCTCCTAATTCATC	2571922
CEA_WeakR130-p2	TATCCTTGGGAAGAACC	1520913	CEA_WeakR398-p2	AGCCTTGCGTAAGTTTT	2573045

CEA_WeakR131-p1	CGGATACACTACTGGTT	1520547	CEA_WeakR399-p1	GTTTCCATTCCCTGTCC	2575273
CEA_WeakR131-p2	TAGCCCTATAAAGATTG	1521997	CEA_WeakR399-p2	GTGTTGCGGTTTGTTGA	2575850
CEA_WeakR132-p1	GATTGCCCGATGACTAA	1521626	CEA_WeakR400-p1	TTTCCCTCATCTATTTC	2582965
CEA_WeakR132-p2	CTTCCTGCTCTTCTCCC	1522347	CEA_WeakR400-p2	TAGTTCCCTTTCTCCTA	2584146
CEA_WeakR133-p1	ACCAGGAGATAGTGAGC	1522184	CEA_WeakR401-p1	CATCCCATAAGAGCATT	2611050
CEA_WeakR133-p2	CTGCTGCTGTAAAGGAA	1523161	CEA_WeakR401-p2	GGTTTGCCATTGTAGTT	2612454
CEA_WeakR134-p1	CAGGCGATGTGGATTTA	1522857	CEA_WeakR402-p1	GAAGAATCAAGCCACCA	2631104
CEA_WeakR134-p2	TCTCGGCAACTGATGGT	1523880	CEA_WeakR402-p2	AGGGCAGTAGTAAGGAAAT	2632242
CEA_WeakR135-p1	CAGGAGGTAATAATGTA	1524008	CEA_WeakR403-p1	TATTTGAGGCATAACTTCC	2632834
CEA_WeakR135-p2	GGCTTCGAGGTTGCTAT	1525215	CEA_WeakR403-p2	TATTGGGCTCCATTTCC	2634198
CEA_WeakR136-p1	TGCAGCAGAAGGTGATT	1524819	CEA_WeakR404-p1	GATAGCCAGTACAATAG	2634060
CEA_WeakR136-p2	TGTATCCTCGGCTCAAT	1526277	CEA_WeakR404-p2	TATGAGGCGATAACTAC	2635292
CEA_WeakR137-p1	CGGAATAACGGTAATAGA	1526921	CEA_WeakR405-p1	ATGCCTGTATTGGAAGC	2635116
CEA_WeakR137-p2	TACCAATGCCTGTCCAT	1527809	CEA_WeakR405-p2	ATTGCAGTTGGAGGTTC	2636266
CEA_WeakR138-p1	TGTTAGCAGACCCACTT	1527640	CEA_WeakR406-p1	CTTGGTGAGCACGTTTA	2636114
CEA_WeakR138-p2	GAATTATTACTTCCATCCA	1529205	CEA_WeakR406-p2	GAATAGAGCGGTGAAAA	2637013
CEA_WeakR139-p1	CTACCTGATAATGCCACTG	1531891	CEA_WeakR407-p1	TTCACCGCTCTATTCAC	2637015
CEA_WeakR139-p2	GAACACTTCCGCTTTGA	1533299	CEA_WeakR407-p2	TTAAAGCCTGGTGGTCT	2638164
CEA_WeakR140-p1	TTGATTATGATTTGCTGAC	1533166	CEA_WeakR408-p1	ACTTCCAAAGCCTCAAT	2639712
CEA_WeakR140-p2	CTCCCTTATCCTCTATTTG	1534266	CEA_WeakR408-p2	TGTTACTCCAGCACCAG	2640631
CEA_WeakR141-p1	AACCGTAGGTATCAACA	1533994	CEA_WeakR409-p1	AGAAACTCCCACATTAT	2641479
CEA_WeakR141-p2	ATTTCAACATAGGCTTC	1534813	CEA_WeakR409-p2	CTTAAATATCACCACGA	2642812
CEA_WeakR142-p1	GATACTCACGCTGTTGC	1534546	CEA_WeakR410-p1	ATTTGCGATAAAAGAGG	2642733
CEA_WeakR142-p2	ACTTCCACCTGTTGTCG	1535242	CEA_WeakR410-p2	GATTATCAACAAGAAAACG	2643352
CEA_WeakR143-p1	GCTCTGCCATAGGTCAT	1535215	CEA_WeakR411-p1	TAACATTCAACCACCCT	2643149
CEA_WeakR143-p2	ATCCCAAACTATCTGCTC	1536243	CEA_WeakR411-p2	AGGAAACACTCCGATTA	2643956
CEA_WeakR144-p1	CCTTTATAGCTCCAACA	1535952	CEA_WeakR412-p1	GAGCCTTTCCTCTTATT	2644382
CEA_WeakR144-p2	GCCATACCCTTTACATC	1537100	CEA_WeakR412-p2	TAGTGGGAGATGTCTTT	2645741
CEA_WeakR145-p1	TTGGGGTAGATGAAAAT	1537235	CEA_WeakR413-p1	AAATCCCAGCCTTACCT	2645488
CEA_WeakR145-p2	AGTTGGACCACCGATAT	1538430	CEA_WeakR413-p2	AGCGGACTTGGACTTTC	2646786
CEA_WeakR146-p1	GTATTCCCGTAATAGAG	1538190	CEA_WeakR414-p1	TATTGGCTCTATCGTGT	2646569
CEA_WeakR146-p2	GTTTACTTGACCTCCTC	1539321	CEA_WeakR414-p2	CTCTTATCTTTGGCTTG	2647232
CEA_WeakR147-p1	GAATTGGAGGAGGTCAA	1539315	CEA_WeakR415-p1	TTTCGCTATGTTTGAATAC	2647013
CEA_WeakR147-p2	ATTACAACGCCTTTACC	1540249	CEA_WeakR415-p2	TTGCTTTGATTGGTCTT	2648335
CEA_WeakR148-p1	TCCTTGTTTTGGTATTC	1541464	CEA_WeakR416-p1	TAGTGCCTGAACTCCTA	2648401
CEA_WeakR148-p2	TATCTTAGGAAGCGGTC	1542675	CEA_WeakR416-p2	GACTTAGCCTACCTTTT	2649313
CEA_WeakR149-p1	GTTTAGGAGCATGAAGG	1542778	CEA_WeakR417-p1	ATATCCTATTCCGTGTT	2649480
CEA_WeakR149-p2	GCTTAGCAGCATATTGA	1543833	CEA_WeakR417-p2	TCAGGTGCAGTTCAGTA	2650958
CEA_WeakR150-p1	AATAATGCTTCTTGATG	1544515	CEA_WeakR418-p1	TCCAAAGGCTTTTATCT	2651759
CEA_WeakR150-p2	CCTTAATAGACTCGACA	1545454	CEA_WeakR418-p2	AATGAAGCAGCATAGGT	2653073
CEA_WeakR151-p1	GTGTTATACCACCATTC	1545296	CEA_WeakR419-p1	AGCCACCACAGCAAATC	2654269
CEA_WeakR151-p2	TTATTGCACTAGCTCCA	1546380	CEA_WeakR419-p2	AACTGCTTCGGGTAAAT	2655475
CEA_WeakR152-p1	TAAATGCCCCTACTAAT	1546106	CEA_WeakR420-p1	GAATTGATTTGGCATAG	2655778
CEA_WeakR152-p2	TACTTTCCACGTCTTCG	1547509	CEA_WeakR420-p2	GAGTTTCATAAGGAGCA	2657268

CEA_WeakR153-p1	GACTAGAGGCGGAGTTG	1547213	CEA_WeakR421-p1	CATTGGCACCTGACTGA	2657356
CEA_WeakR153-p2	TACCCATCCATTGTATTTC	1548356	CEA_WeakR421-p2	GGACTAAAGACGCAGAG	2658475
CEA_WeakR154-p1	AACCTAATGAGGCTGGAC	1548241	CEA_WeakR422-p1	CTCTGCGTCTTTAGTCC	2658459
CEA_WeakR154-p2	GAATCAGTAACAATCCC	1549559	CEA_WeakR422-p2	AGAATGTCAGCCAGGAG	2659860
CEA_WeakR155-p1	CTATGTGGGGATGTATA	1549189	CEA_WeakR423-p1	AGAATTGGGAATCAAGA	2659783
CEA_WeakR155-p2	AATCTTCCTCTGCTGTA	1550348	CEA_WeakR423-p2	CATAACAGCAGTAATAAAG	2661219
CEA_WeakR156-p1	ACTTGCCAGAAGATGAG	1550113	CEA_WeakR424-p1	CCCTACTTTCTATTTCA	2661427
CEA_WeakR156-p2	GCTGCTGAATACCCTAA	1551146	CEA_WeakR424-p2	ATGGATCTAAGGACGAT	2662630
CEA_WeakR157-p1	TTGCTGCAACAGGTATG	1551015	CEA_WeakR425-p1	ACTTAATGTTCGTGGCA	2662415
CEA_WeakR157-p2	CTCGGTTCCTTTATCACTT	1552291	CEA_WeakR425-p2	AATGGATGAAGGGACTA	2663513
CEA_WeakR158-p1	ACAGGCTCACTTGGAAA	1552178	CEA_WeakR426-p1	ACTTGCCTTCAAAACAC	2663367
CEA_WeakR158-p2	ATCAAATACCGCATCGT	1552963	CEA_WeakR426-p2	AGGAAATACAAATAGCG	2664659
CEA_WeakR159-p1	TTGCTTGGTCACGAATA	1552898	CEA_WeakR427-p1	ACCCTAAATGCCCAAAC	2664548
CEA_WeakR159-p2	TAGCCTTAATGTGCTCT	1553817	CEA_WeakR427-p2	AGACCCTAAACCAAATGAA	2666083
CEA_WeakR160-p1	TACAGTGGCTTTGATGA	1554533	CEA_WeakR428-p1	ATTCTTCCACTTTCTTC	2665727
CEA_WeakR160-p2	CTTTTATAGGAGCGTTT	1555703	CEA_WeakR428-p2	TAAACATCCCCATCTAC	2666834
CEA_WeakR161-p1	CAATAAGCTATTTCCAAAG	1555539	CEA_WeakR429-p1	GCAGGTTCACCACAATA	2666404
CEA_WeakR161-p2	CAGCCACAATTCTCACA	1556491	CEA_WeakR429-p2	CGAATAAGGATAGGAGG	2668003
CEA_WeakR162-p1	CCCATGAGGATGTATGT	1556198	CEA_WeakR430-p1	AAAGATAACGAAACCAG	2676806
CEA_WeakR162-p2	ATTTGATGCAGCAGATT	1557047	CEA_WeakR430-p2	AACTACCCAGTGAAGCT	2678391
CEA_WeakR163-p1	TATTAGCTCCTACAGGC	1556668	CEA_WeakR431-p1	CCTCTGCCTTTGCGTAT	2678572
CEA_WeakR163-p2	CATCTAACGCTGAAAGT	1557689	CEA_WeakR431-p2	TGAGGGTGCTTGTAGTC	2680005
CEA_WeakR164-p1	TGCAGTATAGTAATTTTGG	1557564	CEA_WeakR432-p1	CCCACTAACATTTCCTC	2682797
CEA_WeakR164-p2	GGATTTTCGTTGTAGCT	1558962	CEA_WeakR432-p2	ATTCAGTTGTGATTTCG	2684266
CEA_WeakR165-p1	GCGGCTCTATACCTATT	1560375	CEA_WeakR433-p1	TACACCGCTATGATTGA	2684124
CEA_WeakR165-p2	CTTTACTTCTCCCTTGC	1561113	CEA_WeakR433-p2	GCTTATTAAAGGTCCAG	2685798
CEA_WeakR166-p1	AACTTTCCGCAGTTTGA	1560833	CEA_WeakR434-p1	AGCGGTTCTACTTATCC	2685711
CEA_WeakR166-p2	ATCCTTTCGCCTCACTA	1561616	CEA_WeakR434-p2	TTGCCACTGAGTCTATT	2687343
CEA_WeakR167-p1	TACCTTCAGTAATGGCTAC	1563977	CEA_WeakR435-p1	TTGAGGTGCAGAACAAA	2711349
CEA_WeakR167-p2	CCTTCCTCCACAAAGAC	1565635	CEA_WeakR435-p2	AATAGGCCAAAGGAAAT	2712242
CEA_WeakR168-p1	ACCTGGTAGTAAGATTC	1565276	CEA_WeakR436-p1	TTTCCCACGATTTTCTA	2717412
CEA_WeakR168-p2	GTTCCACCATATTCATC	1566907	CEA_WeakR436-p2	ATGACTTTTAGCGTCCT	2718472
CEA_WeakR169-p1	GCACCAGGACCTATCTG	1568019	CEA_WeakR437-p1	TTCCATTCTACCAACCC	2718124
CEA_WeakR169-p2	AACCTCCAGTGCCTCTA	1569438	CEA_WeakR437-p2	GTCCTTTGTTTTATGCTTT	2719506
CEA_WeakR170-p1	GGGGAGAAGTAAAGATA	1569299	CEA_WeakR438-p1	CAACCTCCTTCCGTTAC	2719188
CEA_WeakR170-p2	ATGTGCCTCACTAACAC	1570485	CEA_WeakR438-p2	GTGGTCGTTAGCAGTCATA	2720735
CEA_WeakR171-p1	GTAGAGCTGTGGCCTAT	1570155	CEA_WeakR439-p1	AGCTGTCATCGCTATTT	2722017
CEA_WeakR171-p2	TCCAAATCCGTTCTTAT	1571594	CEA_WeakR439-p2	TTTGCCTATGTTTCCTC	2723471
CEA_WeakR172-p1	ACCTATAAAGCGGACTA	1571610	CEA_WeakR440-p1	TACCGTAACCGTCATTT	2723214
CEA_WeakR172-p2	TATACTGAGGCAAACTG	1572986	CEA_WeakR440-p2	TTCATACCAAGCCATTT	2724038
CEA_WeakR173-p1	CTTCAAGTTATGGGAGG	1572771	CEA_WeakR441-p1	CTTTCTGAAACATTTCGTC	2728094
CEA_WeakR173-p2	GCTATAATACGCTTTCC	1574284	CEA_WeakR441-p2	GTATTGGATTCGCTCAA	2729789
CEA_WeakR174-p1	TCAAGAAGGGTGAGTAAG	1574289	CEA_WeakR442-p1	TTCAAAATCGACTATCC	2729454
CEA_WeakR174-p2	CAGCCATAGAAGCAATA	1575396	CEA_WeakR442-p2	GAACAATTACACCTCCA	2730936

CEA_WeakR175-p1	CACGGTAATAGGGCTCG	1575138	CEA_WeakR443-p1	GTCAATAATTGGTGCTA	2731694
CEA_WeakR175-p2	TCTTCCTCCCCATCTGA	1576303	CEA_WeakR443-p2	AACTTACGAAAGAGGAG	2733034
CEA_WeakR176-p1	CCTGGGCTTCAACTTCT	1575940	CEA_WeakR444-p1	TGAACTATTATTCGCTCTA	2737126
CEA_WeakR176-p2	AGCTGGCTCCTTTATTT	1576894	CEA_WeakR444-p2	TAAGGATACCTCCCCTA	2738487
CEA_WeakR177-p1	TATTACCTTAGGTGGTT	1576445	CEA_WeakR445-p1	TTACCTCCATATTTCGC	2759694
CEA_WeakR177-p2	GTGTATTTCCCTTATCT	1577261	CEA_WeakR445-p2	CTATTAGTCCGCTGGTT	2760745
CEA_WeakR178-p1	GGATGTTAGTATCGGAGAA	1592472	CEA_WeakR446-p1	TTATCACTTTTCTGCGTAT	2762434
CEA_WeakR178-p2	CAACTTCCAAAGCAATC	1593157	CEA_WeakR446-p2	GTTATTGCTGCTTCTGG	2763547
CEA_WeakR179-p1	ACTTTTGGTTATCTTTGTG	1607609	CEA_WeakR447-p1	TCAACTTTTAGGCTCTT	2768588
CEA_WeakR179-p2	TCTTCCATACTGATTCCTC	1608354	CEA_WeakR447-p2	ACAATGATAGATATGGC	2769701
CEA_WeakR180-p1	ACATGGAGGATAAAGAT	1617234	CEA_WeakR448-p1	TATGAAGGCTGGTGAAG	2771056
CEA_WeakR180-p2	TTTGTAGCCTGATCTAA	1618246	CEA_WeakR448-p2	GGTAATAGAGGCAATGAAA	2772212
CEA_WeakR181-p1	TAGGAGGAACTACAGCA	1630156	CEA_WeakR449-p1	ACCATATCAACGGTTCT	2773967
CEA_WeakR181-p2	TGTGCCTATCTGGAGAC	1630982	CEA_WeakR449-p2	GATGTACTGAGGCTGGA	2775330
CEA_WeakR182-p1	TAATAATGAGCAGAAAC	1632975	CEA_WeakR450-p1	TATCCAGGATGTATAGCG	2775392
CEA_WeakR182-p2	ATATGCTATCACCTCAA	1633762	CEA_WeakR450-p2	CAATCTGTTGCACCAAG	2776506
CEA_WeakR183-p1	CTCCATTACGAGGTGAA	1640060	CEA_WeakR451-p1	TTAGATGGCCCTTCATT	2776544
CEA_WeakR183-p2	GTGAATAGCCCTCTTTG	1640681	CEA_WeakR451-p2	TAATCGGCATCAGGATA	2777485
CEA_WeakR184-p1	AGATAAAGGGTATAGGA	1643367	CEA_WeakR452-p1	CCCCGACCTTCAGATTC	2778223
CEA_WeakR184-p2	ATGAACCGAATCAAGTA	1644721	CEA_WeakR452-p2	CCGATAAGCCACCAAAT	2778967
CEA_WeakR185-p1	CTTGGACGTGTATCTGG	1648359	CEA_WeakR453-p1	CACTTCCCGTCATACCT	2795315
CEA_WeakR185-p2	CATCAGCACCCATTCTA	1649804	CEA_WeakR453-p2	ACCTAAGTTCGCCACAG	2796559
CEA_WeakR186-p1	TTTGTTCCTTCCTCCAT	1650243	CEA_WeakR454-p1	ACGAAATCCCTCAATAA	2797359
CEA_WeakR186-p2	GCCCTCTTAACCCTAAA	1651194	CEA_WeakR454-p2	CAGCCTTCTCCATACAA	2798972
CEA_WeakR187-p1	AAGTCGCTAATGTTTGT	1652108	CEA_WeakR455-p1	TTGATCTCATGGGAATT	2798908
CEA_WeakR187-p2	AATATCGTCCTCACCTC	1653668	CEA_WeakR455-p2	AGGCATTTATGGGAACT	2800143
CEA_WeakR188-p1	ATACAATGGGAGGTGAG	1653643	CEA_WeakR456-p1	AAGCTACAATCCTCCAT	2801237
CEA_WeakR188-p2	CTTATTGAATTGGTGGT	1654346	CEA_WeakR456-p2	CATAATAACTTACCTCCAT	2802213
CEA_WeakR189-p1	CTGGCAGGAATGTATGT	1654776	CEA_WeakR457-p1	TCCTCCTTCAAGGTTAT	2803841
CEA_WeakR189-p2	TACAGCGTCCACAGTTA	1656125	CEA_WeakR457-p2	ATAGGAATGGAGCAATA	2804807
CEA_WeakR190-p1	CTCCAGCAATGATGAGA	1657064	CEA_WeakR458-p1	GGTGATTATACACGCTTTG	2806946
CEA_WeakR190-p2	ATATCCCTGCACCAATA	1658499	CEA_WeakR458-p2	TGTCAACCTGGTCCTTC	2808030
CEA_WeakR191-p1	ATAACCAAGCCTTTCTC	1658610	CEA_WeakR459-p1	ACAATGAAACGGTGCTT	2814642
CEA_WeakR191-p2	TTCCCATACCTGTAGAT	1659931	CEA_WeakR459-p2	ATTCGGATCTGGGTATG	2815327
CEA_WeakR192-p1	TGGAAGAACTTAGTATAG	1659590	CEA_WeakR460-p1	CAGCAACCTCATTAGCA	2816040
CEA_WeakR192-p2	ATATTAGGGTAGGATTG	1660667	CEA_WeakR460-p2	GCCCAAGTTACAGCACC	2817296
CEA_WeakR193-p1	AAAACTATGGGACAAATGG	1661095	CEA_WeakR461-p1	GATCACAGAACATGCCTAC	2817048
CEA_WeakR193-p2	AGGAACTTGTGCCTTCA	1662446	CEA_WeakR461-p2	GAAGCTAACGCATTGAA	2818602
CEA_WeakR194-p1	CAGGTATGCCGCTAACA	1666784	CEA_WeakR462-p1	CATAGAACCCATCATAA	2822819
CEA_WeakR194-p2	ATCTGCCTTTGGCTTTT	1667715	CEA_WeakR462-p2	GAGTGCATACATAGTGC	2824282
CEA_WeakR195-p1	AAAGCATTGTAGGGTAA	1668144	CEA_WeakR463-p1	ACATCCATATCTGCCATCA	2833964
CEA_WeakR195-p2	TCTCCACTTTCGTGTCT	1669777	CEA_WeakR463-p2	CAACGCAAGATTTGGT	2835221
CEA_WeakR196-p1	CTTACAGGCGTATGGGG	1682295	CEA_WeakR464-p1	ATAAATAGGAGGACCAA	2839598
CEA_WeakR196-p2	TGATTTAAGCGGTGGTG	1683193	CEA_WeakR464-p2	TCAGTAGATGCTGGAAA	2840456

CEA_WeakR197-p1	AATACATCAAGGAGGAG	1682958	CEA_WeakR465-p1	CTAACCCATAGCATCTT	2841548
CEA_WeakR197-p2	TCAACACTAATGCCAAC	1684068	CEA_WeakR465-p2	TAGCTCTAGGGACTTTT	2843100
CEA_WeakR198-p1	AGGAATTGAGGGCTTTG	1708462	CEA_WeakR466-p1	GCAAATACCTGTCTTGA	2842915
CEA_WeakR198-p2	ATCCGAACATAAGTCCAAG	1709331	CEA_WeakR466-p2	AATGATAATGGAAGTGG	2844184
CEA_WeakR199-p1	GGAAATATCCCTTAGTA	1714220	CEA_WeakR467-p1	CCACAATCCCATACACT	2846255
CEA_WeakR199-p2	AATAGCCTGTAGATTGA	1715528	CEA_WeakR467-p2	TAGGCGGAGGAACTTAT	2847632
CEA_WeakR200-p1	ATAAGGGAGAACAAACA	1719544	CEA_WeakR468-p1	ACTGTAGGTCCCTTGTC	2848517
CEA_WeakR200-p2	TCTTCCTAAGCCATCTA	1720209	CEA_WeakR468-p2	AACTCTTGGGTATTTCG	2849965
CEA_WeakR201-p1	CGGCATTGGTATTTCTA	1726229	CEA_WeakR469-p1	AAAAGAAGTAGCCTATG	2853194
CEA_WeakR201-p2	ATTAACACCCACCTTCC	1727268	CEA_WeakR469-p2	GAAGCAATGAAAGATGA	2854524
CEA_WeakR202-p1	TTTCCAGATTCGGTTAG	1731343	CEA_WeakR470-p1	CTGTCAAAAGCTCATTG	2854313
CEA_WeakR202-p2	CTTTAACTGCCTTTGTC	1731494	CEA_WeakR470-p2	TAGACGAAAAGCATCAT	2854940
CEA_WeakR203-p1	TACCAGCAGTACCACCG	1732390	CEA_WeakR471-p1	AAGGCTTCATCATTCAA	2855418
CEA_WeakR203-p2	ACTGCTGGTATTGGTGT	1732384	CEA_WeakR471-p2	AGTCATGGGCACGTTTA	2856256
CEA_WeakR204-p1	ACTAAAGCCTAGCAGTC	1734478	CEA_WeakR472-p1	TTTTCACGAAATACGCT	2858672
CEA_WeakR204-p2	CCAGCACCGATTCTATT	1733432	CEA_WeakR472-p2	AGACGGGGCAGATGACT	2860047
CEA_WeakR205-p1	ACAGTTAGGGCTTATTT	1737864	CEA_WeakR473-p1	AAGTTTCCCAAGTTCCT	2861090
CEA_WeakR205-p2	TACTATGCGTTTGTTCT	1735842	CEA_WeakR473-p2	TGGGCTAAGTATAGTGAGT	2862498
CEA_WeakR206-p1	CATTTGCCTTGCTTGAC	1738148	CEA_WeakR474-p1	CTTGCTCTGAATTTTCT	2862447
CEA_WeakR206-p2	CCAGGCGTCTAGGAGTA	1738418	CEA_WeakR474-p2	TTGATATTACAGGGGAT	2863699
CEA_WeakR207-p1	TTGGTATTGCTCGTATG	1738577	CEA_WeakR475-p1	CATACCTTCCACCTGAA	2867237
CEA_WeakR207-p2	TATTCGTAGTTTGGGTT	1738919	CEA_WeakR475-p2	TTTAACAGTGCCGTTTT	2867887
CEA_WeakR208-p1	AATAGGTATTGGTGGAG	1740318	CEA_WeakR476-p1	TGTAACCGAAAGCATAG	2875789
CEA_WeakR208-p2	GTTCTTGGTTTATTCCTAT	1739270	CEA_WeakR476-p2	AGTAAGAGCGGAGAAAG	2876372
CEA_WeakR209-p1	AGACCTTTACTGATTTTAC	1741497	CEA_WeakR477-p1	GTGCTGGAACATTTGGA	2962051
CEA_WeakR209-p2	GACATTCATTACCACCT	1743146	CEA_WeakR477-p2	ACAGGACTTTGGCTTCG	2962891
CEA_WeakR210-p1	CATTATGCAAATTAGAGG	1742831	CEA_WeakR478-p1	CTTCTCGCAGTCATTTA	2966430
CEA_WeakR210-p2	GCTATCAAGAACAAACG	1743484	CEA_WeakR478-p2	TCCATAGTTATCCTCCA	2967295
CEA_WeakR211-p1	TTTCTGCTTCCACTAAT	1744411	CEA_WeakR479-p1	TTCCCACATCCTACAAG	2966950
CEA_WeakR211-p2	CTTCCCTCCTACAACTA	1745520	CEA_WeakR479-p2	GACTCCCATTATTCTGC	2968354
CEA_WeakR212-p1	GAAAGTATTCCCAAAGG	1745375	CEA_WeakR480-p1	GTTTGGATATTTGCTTTTC	2980222
CEA_WeakR212-p2	GTTCATCAAATGCTCCC	1745940	CEA_WeakR480-p2	AGGTTTAGACGGAGGCT	2981111
CEA_WeakR213-p1	TGCTGAAGGGATGAGTC	1745581	CEA_WeakR481-p1	AAGGTAACAATTCCAAG	2982595
CEA_WeakR213-p2	TTCTTAAAAGGTCGGTC	1747139	CEA_WeakR481-p2	TTATAGCGATAGGTGAT	2983728
CEA_WeakR214-p1	GAAGGGCATACTTTGAT	1749992	CEA_WeakR482-p1	CAAAGGCTTATCAACTC	2984844
CEA_WeakR214-p2	GGCTTAATAACCTCCTG	1751239	CEA_WeakR482-p2	TAAACTCACCTCCATAT	2986151
CEA_WeakR215-p1	GCTATTGATACGCTTTC	1752286	CEA_WeakR483-p1	TTACCAGCATCAATAGC	2993804
CEA_WeakR215-p2	CCTATTGTTCTGGCTGT	1753459	CEA_WeakR483-p2	AGATAAGGCAAACGAAT	2995087
CEA_WeakR216-p1	TAGTTGGAATGGGAACG	1753649	CEA_WeakR484-p1	TGTTCCTGCTTCTATCC	2996329
CEA_WeakR216-p2	ATTTCTCCCGTATTGTT	1754680	CEA_WeakR484-p2	GTCTTGAGGAAGCTGTG	2997397
CEA_WeakR217-p1	AAACAATACGGGAGAAA	1754679	CEA_WeakR485-p1	GCTTCCTCAAGACTATT	2997401
CEA_WeakR217-p2	ATCTGTGGGCTATTACA	1755924	CEA_WeakR485-p2	GAAGTTGCTAACAAGGT	2998739
CEA_WeakR218-p1	AGATAGAGGGATAAAGG	1755981	CEA_WeakR486-p1	CATCTTCAGCCTTTTCT	3002930
CEA_WeakR218-p2	CTACAGGTATAAGGAGC	1757523	CEA_WeakR486-p2	CTCATTTTATAGCCCTTAT	3004350

CEA_WeakR219-p1	GCTCCTTATACCTGTAG	1757507	CEA_WeakR487-p1	GTTTTCTATTCCCTTTC	3007115
CEA_WeakR219-p2	GTTGATAAATCCACCTT	1758335	CEA_WeakR487-p2	TCTTGGAGGATTTATTG	3008590
CEA_WeakR220-p1	TTTGGCAGTAAAACATA	1758299	CEA_WeakR488-p1	TTATTGGCTCGTAGTCT	3016027
CEA_WeakR220-p2	GTCCATCTAACATTCTATC	1759185	CEA_WeakR488-p2	GGAAGTATTAAGGTGGA	3017482
CEA_WeakR221-p1	ACCTAGAAAGGCTGAAC	1833859	CEA_WeakR489-p1	CTGCGAAGGTGAAACAT	3019813
CEA_WeakR221-p2	CTTGAAAACTAACATCG	1834549	CEA_WeakR489-p2	ACAGGACCAGAGTAGCC	3021242
CEA_WeakR222-p1	TGGCATAAGCAGGGAGT	1836491	CEA_WeakR490-p1	TAAGCGTTGTGGAACAT	3020952
CEA_WeakR222-p2	ATCATTTGCGCTACAGG	1837244	CEA_WeakR490-p2	AAACAGCAGCAGGTAGA	3022063
CEA_WeakR223-p1	GGAGGAAATCTTAAACC	1848684	CEA_WeakR491-p1	TGTCTGACTTCCAGCAT	3021504
CEA_WeakR223-p2	CAAACTCATAGCAGCAT	1849452	CEA_WeakR491-p2	ATAAAGAGCGGAGCAAT	3022498
CEA_WeakR224-p1	ATAGAAACAAGCAGCGTAG	1863636	CEA_WeakR492-p1	CATTTGTTACCCTTATTCG	3040389
CEA_WeakR224-p2	TAGCCTGGATTTGGAAC	1864509	CEA_WeakR492-p2	TGCCGTCAATTCCTATT	3041289
CEA_WeakR225-p1	AAGAAATAGCACCAGAT	1871601	CEA_WeakR493-p1	AAAGCTGGCTTGGTTAA	3157307
CEA_WeakR225-p2	AGTCCCATAATAAACCT	1872580	CEA_WeakR493-p2	CTCCTGGTACTATTGTTGA	3158173
CEA_WeakR226-p1	TTCCAAATGCCAGTGTA	1884832	CEA_WeakR494-p1	GTGAAGCCTATGTACTGT	3290889
CEA_WeakR226-p2	TAGCATAAGGAGGGTCT	1885684	CEA_WeakR494-p2	ATGGGATACTTTGGATG	3291698
CEA_WeakR227-p1	TGGAGGATGTATCAAAG	1886297	CEA_WeakR495-p1	CATACTGTCCTCCAAAT	3293149
CEA_WeakR227-p2	TCAAGGTGTAATAAGCA	1887122	CEA_WeakR495-p2	AAGTGAACTCTAAACCC	3294260
CEA_WeakR228-p1	TATGGACCCAGCAGTAG	1891385	CEA_WeakR496-p1	TTTTGAAACGCCTTACC	3294611
CEA_WeakR228-p2	AAGACAGCGTGAACAAG	1892180	CEA_WeakR496-p2	GGAACAGGAGGAGATGG	3295334
CEA_WeakR229-p1	ATAATAGAACTTGGTGTAC	1895142	CEA_WeakR497-p1	GTTCCTCCATTGGTGTC	3309733
CEA_WeakR229-p2	GAAACATAAAAGCACTC	1896531	CEA_WeakR497-p2	CGAATATCTGTCGCTGT	3311069
CEA_WeakR230-p1	TGAGATTGACGATTTTG	1899098	CEA_WeakR498-p1	TTCCACCAACACCATCC	3319153
CEA_WeakR230-p2	GGTCTTTTACCTTGTTTT	1900335	CEA_WeakR498-p2	CCGTCAACAGCCCTAAC	3320399
CEA_WeakR231-p1	TCGCTTTGATGATTTAG	1903585	CEA_WeakR499-p1	CTTTAGAAACGCCCTTAG	3322629
CEA_WeakR231-p2	GCCATTTCTCCTCCTGT	1904395	CEA_WeakR499-p2	TTTGGTATTTGCTGCTT	3323700
CEA_WeakR232-p1	AGCCGTAGGTATCACTC	1907639	CEA_WeakR500-p1	CTGCGTATTTCAACCTC	3374832
CEA_WeakR232-p2	ATTTATGTCTTCGCCAC	1908150	CEA_WeakR500-p2	CTTGTAGGACCGCTTAT	3375369
CEA_WeakR233-p1	CATAGAATGCTACGGAA	1910815	CEA_WeakR501-p1	TGTTAATGTTCCCACTG	3477524
CEA_WeakR233-p2	TCCTGACCTCATAATCG	1911947	CEA_WeakR501-p2	ATGCAGCAAGTCAACTA	3478777
CEA_WeakR234-p1	TAAGCATCTCCACCAGC	1912946	CEA_WeakR502-p1	CCAAATAAAACAACTACTC	3485643
CEA_WeakR234-p2	GCAAATAATGCCGAAAA	1914188	CEA_WeakR502-p2	GGTTAAAGACAAAGGATA	3486557
CEA_WeakR235-p1	AAAACGTACAAATACTAGC	1914044	CEA_WeakR503-p1	GCTTTAAGTGAAGTGGC	3513139
CEA_WeakR235-p2	CAAATTCAATTCCCCAT	1915206	CEA_WeakR503-p2	GTATGTTGACCCGAACC	3514353
CEA_WeakR236-p1	ACATAACCAAGACATTC	1915306	CEA_WeakR504-p1	AAACTTCATCTTCGCCTCT	3530633
CEA_WeakR236-p2	CACTACTAAAACCTCCA	1916332	CEA_WeakR504-p2	TGGTGCGGATTATTGGT	3531504
CEA_WeakR237-p1	TGAAAGGAAGAAAATAGTG	1916291	CEA_WeakR505-p1	TAGCCATTCTACTCATC	3533847
CEA_WeakR237-p2	TAGTTTGAGGAACAGCA	1917632	CEA_WeakR505-p2	CACATAGTTGTAAGCAA	3535336
CEA_WeakR238-p1	ACCAAATTCATCAACAC	1921147	CEA_WeakR506-p1	TCAAATAGTGATCCTGCTA	3535153
CEA_WeakR238-p2	ACTAAGCATAGCCTCAA	1922190	CEA_WeakR506-p2	AGATGCTCTAGGGCTTG	3536732
CEA_WeakR239-p1	TTACAGGGCCTACTACA	1922634	CEA_WeakR507-p1	TCTACCCGCTTGACTTG	3539136
CEA_WeakR239-p2	ACATAAGCCTATCCTCA	1923488	CEA_WeakR507-p2	TAGAAACGGAGTTAGGT	3539968
CEA_WeakR240-p1	GTTACATGGTCGCTTTA	1923002	CEA_WeakR508-p1	ATACCATTGCCACTATCC	3543306
CEA_WeakR240-p2	ATTACCTCCCTTCTTTT	1924015	CEA_WeakR508-p2	ATGGGAACTCCACTCAA	3544455

CEA_WeakR241-p1	TCATATTGCCAAGGGAT	1923857	CEA_WeakR509-p1	ATCTTAGGCATTTTGTC	3546173
CEA_WeakR241-p2	TATGCTGCCAATCTTCA	1925152	CEA_WeakR509-p2	TCATAAGTACCCTCCTC	3547082
CEA_WeakR242-p1	GATTGGCAGCATACTTT	1925172	CEA_WeakR510-p1	GATGAAACTGGCGATGC	3553891
CEA_WeakR242-p2	TTATCACCGAATCCTTT	1926546	CEA_WeakR510-p2	GTGGCGGTAGATGTGAG	3554889
CEA_WeakR243-p1	AGGCATCAAATGATTAA	1926406	CEA_WeakR511-p1	GCATCGAAACACCTATT	3562804
CEA_WeakR243-p2	ATTGTTCCAGAAGCTGG	1927056	CEA_WeakR511-p2	TAGTGCCAGGGTTATTG	3564026
CEA_WeakR244-p1	CGGTATTGGAATTTGGT	1926947	CEA_WeakR512-p1	CAGCCAGCAGTTATCAT	3609568
CEA_WeakR244-p2	ATCCCTGAGATTATCTGTA	1928240	CEA_WeakR512-p2	GTTAATCCAACGCAAAA	3610251
CEA_WeakR245-p1	CTCCCTCAGCTTCACAT	1927966	CEA_WeakR513-p1	GCAGTAGCTCCCGTTTC	3618081
CEA_WeakR245-p2	CTCTATTCTACCCATAT	1929434	CEA_WeakR513-p2	GTGGCGTGGGTAGTTTT	3619549
CEA_WeakR246-p1	CCCGATAAAGAGGGTAA	1930193	CEA_WeakR514-p1	CTCTTATCTAAAACGTG	3621450
CEA_WeakR246-p2	CGACAACAAGCGTAGAA	1930974	CEA_WeakR514-p2	TAGTAAAGACAGCAGAA	3622139
CEA_WeakR247-p1	GAAAATAGTAGCCTGAT	1931172	CEA_WeakR515-p1	AAAACTTTCCTCTTACA	3623630
CEA_WeakR247-p2	GTAGCAAACCATAAATC	1932397	CEA_WeakR515-p2	CGTTATGGATACAGCAA	3624579
CEA_WeakR248-p1	CATTAGCCTCTGTGGTA	1931974	CEA_WeakR516-p1	GGAGCAGACGGAGGATT	3624161
CEA_WeakR248-p2	GACGCTGTTATTATCGA	1933073	CEA_WeakR516-p2	CTTGCTTGGCAGGTCAT	3624920
CEA_WeakR249-p1	ATGGTACAATCGGTCTT	1934355	CEA_WeakR517-p1	CTGGCTTATTAGTTTCC	3627046
CEA_WeakR249-p2	TATCAGCGGTAAACTCA	1935049	CEA_WeakR517-p2	AATTGTAGAAGGGCTTA	3628476
CEA_WeakR250-p1	TGCGGTAGCTTTATGGT	1935464	CEA_WeakR518-p1	ATTTGACGCTCTGAAGG	3632472
CEA_WeakR250-p2	TTGCTTCTTGAGCCTGT	1936956	CEA_WeakR518-p2	TGAGGTGCCAGAATGGT	3633803
CEA_WeakR251-p1	ATTTCGGACATCAAACA	1936724	CEA_WeakR519-p1	ATGGAATACACCCAAAT	3643004
CEA_WeakR251-p2	CTCCAATTCTTCCATCA	1937966	CEA_WeakR519-p2	TAATGGCAAACTAAGGA	3644085
CEA_WeakR252-p1	GGAACAATCCAAAGCTA	1937924	CEA_WeakR520-p1	TCATAACGCACAAGTAA	3673143
CEA_WeakR252-p2	GAAACATCAAGACCAAATA	1939099	CEA_WeakR520-p2	AAGCCACATAAGGATAA	3674188
CEA_WeakR253-p1	TCGGGAAACATAAGGAA	1939116	CEA_WeakR521-p1	CTTTCAGCACTTGTCTG	3729141
CEA_WeakR253-p2	ACAAGCCTCTGGCAATC	1940215	CEA_WeakR521-p2	GCATTGGAAATGGTAAT	3730012
CEA_WeakR254-p1	TCTTCTTTGGCAATGTG	1940501	CEA_WeakR522-p1	GCGATTTCACCCTATGT	3788036
CEA_WeakR254-p2	AAAGTTTGCGTCGTCAT	1941681	CEA_WeakR522-p2	AAAAGCCACTGTTTTCA	3788969
CEA_WeakR255-p1	CAGAGGACAGAAAGCAT	1941495	CEA_WeakR523-p1	CGGTGTTTATGATTTGA	3815096
CEA_WeakR255-p2	CTTTAAGACACCTACGG	1942880	CEA_WeakR523-p2	TGTTGGTGCTTTGATTT	3816125
CEA_WeakR256-p1	TAAAGTTTTCGGGGATA	1943147	CEA_WeakR524-p1	AACCCTTACCTGCTTCC	3892253
CEA_WeakR256-p2	TTAAATGCTTCGCTGTC	1944037	CEA_WeakR524-p2	ATTACAATACGGCTACT	3893040
CEA_WeakR257-p1	TGCTTTCAAACTGTGCT	1955612	CEA_WeakR525-p1	GCCACCAATGATATTCA	p23836
CEA_WeakR257-p2	TTCGTTCATCCCTATCC	1956331	CEA_WeakR525-p2	CAAAGGAGGACACCAAC	p24518
CEA_WeakR258-p1	ATATTCCCCTTAACCAC	1992704	CEA_WeakR526-p1	CGCCTTGTGAGGTAAAG	p69895
CEA_WeakR258-p2	AAATGGAAGTAATGCTG	1993345	CEA_WeakR526-p2	CAAATGAAGCTGGAAAA	p70751
CEA_WeakR259-p1	GTCCTGTAATAATGCCTAT	1997700	CEA_WeakR527-p1	ACTTTACCTTCTGGCTGTT	p73721
CEA_WeakR259-p2	AAGTTGTTGAGGGAGAA	1998435	CEA_WeakR527-p2	GGCGTGCTAATTTGTTT	p74853
CEA_WeakR260-p1	TAATTGCCACATCAAAG	1998566	CEA_WeakR528-p1	TTTAGGTAGCCAAGTAC	p94304
CEA_WeakR260-p2	GATAATCTAAAGGAAAACG	1999697	CEA_WeakR528-p2	ATTATCTCATAAGTGCC	p95217
CEA_WeakR261-p1	GAGTTAAAAGTGCCATA	1999826	CEA_WeakR529-p1	CATTTGCAGCGTGTTCT	p97167
CEA_WeakR261-p2	TTTGCGGAGAAATAGTG	2001259	CEA_WeakR529-p2	TTGACCCAAGCCTCATC	p98380
CEA_WeakR262-p1	GTCTGTGGGTATGATTT	2001506	CEA_WeakR530-p1	CGGTTGGCTACTTTCTA	p120898
CEA_WeakR262-p2	AACTTCTGATGATTTGC	2002354	CEA_WeakR530-p2	GTTTCACAGGCATCTAC	p121984

CEA_WeakR263-p1	AGGATACCACGGAAACT	2003259	CEA_WeakR531-p1	CAGCCTTTATTGGTTCT	p131519
CEA_WeakR263-p2	ATGACAACTGCCACAAA	2004255	CEA_WeakR531-p2	CTTTTACATTAGGGTTTTG	p132599
CEA_WeakR264-p1	ATTTAGCCCATTCGTTG	2007067	CEA_WeakR532-p1	AAATCGGGCTAAGACAA	p136952
CEA_WeakR264-p2	ATATTGCATCTGCCTGT	2008218	CEA_WeakR532-p2	TAATAACGCTGCACTCC	p137937
CEA_WeakR265-p1	TATTCCAAGCCTTCTTA	2010125	CEA_WeakR533-p1	TGTCGTATAAATCCCTT	p143919
CEA_WeakR265-p2	ATTACTACATGCTCCAC	2011406	CEA_WeakR533-p2	CCTTAACTGCGATGAGT	p145173
CEA_WeakR266-p1	GTTGCCTTATCTGTTTC	2012333	CEA_WeakR534-p1	TTGGGAAATATGGAATG	p159975
CEA_WeakR266-p2	TGTGATGCTATGGGAGA	2013500	CEA_WeakR534-p2	AAACGCCCTAAGTATGA	p161086
CEA_WeakR267-p1	TTAAACGAATCGGAAGT	2019785	CEA_WeakR535-p1	GTTAATACCCCTGTACC	p168684
CEA_WeakR267-p2	ATATGCCAAGTATAGCC	2020860	CEA_WeakR535-p2	ACAGAAAACCAAAACTC	p169826
CEA_WeakR268-p1	ATTAGCCATTTCACCAA	2020648	CEA_WeakR536-p1	GTTTCGTCTTTCATCTT	p170899
CEA_WeakR268-p2	AAAGGCACAATTACAGG	2021496	CEA_WeakR536-p2	CATCCCACTTCTTATCT	p171737

* The location in genome: the number means the primer starting sites in EA 2018 genome. “p” indicates large plasmid in EA 2018.
