# Supplementary material for: Comparative genomic and transcriptomic analysis revealed genetic characteristics related to solvent formation and xylose utilization in Clostridium acetobutylicum EA 2018
Source: BMC Genomics. 2011 Feb 2;12:93. doi: 10.1186/1471-2164-12-93 (PMC3044671; doi:10.1186/1471-2164-12-93)
Supplement: Additional file 3 — Variation places between EA 2018 and ATCC 824 verification primers. This file lists the verification primers for all of the indels sites and SNVs sites found between EA 2018 and ATCC 824. [file 1471-2164-12-93-S3.PDF]

Additional file 3. Variation places between EA 2018 and ATCC 824 verification primers

Name	Sequence	Location in genome *	Name	Sequence	Location in genome *
CEA_Homo1-p1	GATTCTGCCAACCTTTA	12	CEA_Homo224-p2	CACCCATTAAATTGTCC	1907275
CEA_Homo1-p2	TTTTGCCACAGTTCATT	483	CEA_Homo225-p1	GGCTCTAACTCTTTCTC	1910090
CEA_Homo2-p1	TACGGAAGAAGCAAGAG	23727	CEA_Homo225-p2	ATGCCTTCTACTATTTC	1910810
CEA_Homo2-p2	ATGACCATCGGCAATAT	24303	CEA_Homo226-p1	TTTTGGGCAGAAACCTA	1911543
CEA_Homo3-p1	CAAACCGTAGATAAACC	71395	CEA_Homo226-p2	TTAATAATGGCCTCACT	1912168
CEA_Homo3-p2	CTAACGCAAACCTGTAA	72287	CEA_Homo227-p1	AAAGGGGAATGGAGGTA	1913808
CEA_Homo4-p1	GTTATCCAAAACCATAC	75776	CEA_Homo227-p2	AAGGGTTCTTGAAATCG	1914446
CEA_Homo4-p2	GAGTTTTATTCTGCTTATC	76288	CEA_Homo228-p1	TATCTTCGCTAATGGTT	1919569
CEA_Homo5-p1	GCAGAAAGCATAAAGAG	88438	CEA_Homo228-p2	TTCAAGTAGTGGTGGTA	1920403
CEA_Homo5-p2	TCTTCGCATACAAATAC	88979	CEA_Homo229-p1	TAAAGAAACAGGAAAGG	1927596
CEA_Homo6-p1	TGTAGCAGAAAGCATAA	88585	CEA_Homo229-p2	TCTATGGTAAGCAGGTA	1928106
CEA_Homo6-p2	AGAGTCGTCCAATTCAC	89474	CEA_Homo230-p1	TTTAGTTACTGAGGCAGAT	1933042
CEA_Homo7-p1	AGATGCAGCTACTCTTG	120801	CEA_Homo230-p2	TTAAGAGCAACGGATTG	1933998
CEA_Homo7-p2	ATTCGCCAATTATCAAC	121413	CEA_Homo231-p1	CGATTGGTGGAGTTCTA	1941178
CEA_Homo8-p1	GTAAGGTATAGTAAGGGAT	135640	CEA_Homo231-p2	AGTTTGCGTCGTCATAT	1941723
CEA_Homo8-p2	ACCAAGATGAGGCAAAC	136130	CEA_Homo232-p1	AGTTTATTAGGGACAAC	1942459
CEA_Homo9-p1	TGGAATAAGTGCCTGTA	155163	CEA_Homo232-p2	GTTAGTATGCCATCAGT	1943432
CEA_Homo9-p2	CAGACGGAATGAGAAGT	155695	CEA_Homo233-p1	AGTTTATTAGGGACAAC	1942475
CEA_Homo10-p1	GGAGTTTGGCAATGTAA	196724	CEA_Homo233-p2	GTTAGTATGCCATCAGT	1943438
CEA_Homo10-p2	CACGTTCTAAAAGGAAA	197163	CEA_Homo234-p1	TGATGTTAGAGGCTGGTG	1956125
CEA_Homo11-p1	GGAGTTTGGCAATGTAA	196774	CEA_Homo234-p2	CTTGCTTGGGATGTTGA	1956875
CEA_Homo11-p2	AAGAATGGAGCTGATGG	197306	CEA_Homo235-p1	TGTTGAGGCTGGTCTTG	1959610
CEA_Homo12-p1	TTGGCATACATCCTTGA	205406	CEA_Homo235-p2	AATTTCTCCTGCCGATT	1960580
CEA_Homo12-p2	GCGTTGCTAAATACCCT	205957	CEA_Homo236-p1	GGCAAGAGGTATTGTTA	1967263
CEA_Homo13-p1	TGCCACTTCCAACAATA	238454	CEA_Homo236-p2	TCAATGTCAGTGAAAGC	1967769
CEA_Homo13-p2	CATCTCCGTGAGCCATA	238886	CEA_Homo237-p1	GCAGGGTAAGAGTATGA	1968615
CEA_Homo14-p1	TGATTTGCTCAACCATT	247560	CEA_Homo237-p2	ATAGCCTAATCTCCATC	1969559
CEA_Homo14-p2	TCCATTTCGTCACCTTC	247958	CEA_Homo238-p1	TGGGATACCTAAATACG	1976403
CEA_Homo15-p1	TAATGCAGGAACTTTGG	249437	CEA_Homo238-p2	ATAAACGGAACTCTTGC	1977115
CEA_Homo15-p2	CACTTCTTGTCCGTCTC	250233	CEA_Homo239-p1	GTAGAACCCCTAAAGGC	1980748
CEA_Homo16-p1	ATTCTACGGAAAGGATA	269362	CEA_Homo239-p2	TCTTGCATAGCTTCCGT	1981315
CEA_Homo16-p2	ACCATTAGTACAAAGCT	270187	CEA_Homo240-p1	TCAAAAGAAGAAGGATT	1980805
CEA_Homo17-p1	GTGGGTTGTTGATGGTA	270050	CEA_Homo240-p2	ACTTTCTTCAAGGGTTA	1981441
CEA_Homo17-p2	ATAACGCCAGGAAGTAA	270437	CEA_Homo241-p1	TAGCGCCTGCATCAATA	1987707
CEA_Homo18-p1	ATCAAATAAACTTCCAGAG	305882	CEA_Homo241-p2	AAGTAGGGCGAGGAACA	1988355
CEA_Homo18-p2	CAGAAGGAAATCCAATC	306301	CEA_Homo242-p1	ATCTCATCCTTGCTTAT	1992496
CEA_Homo19-p1	TACCTGCATAGTCAAAG	321271	CEA_Homo242-p2	GAGGGTATTATGTTTACTT	1993103
CEA_Homo19-p2	TGTTCTTATCCACCTCT	321790	CEA_Homo243-p1	ATTCCTAAAGTTTCTAAC	1995829
CEA_Homo20-p1	ATAGGAACAGCTCCTTA	322835	CEA_Homo243-p2	TTCAATACAAGCATACG	1996352

CEA_Homo20-p2	ATTGAATCCATCACATC	323221	CEA_Homo244-p1	TGAAGGCTTCACCTCTG	2012847
CEA_Homo21-p1	ATTTGCTCACCATCTAC	363920	CEA_Homo244-p2	GGTTGTGATGCTATGGG	2013558
CEA_Homo21-p2	AACATTTCGACACTTTG	364409	CEA_Homo245-p1	TTTTCTGCCATGTCAAT	2014290
CEA_Homo22-p1	GTATCGTTTGATTTATC	377944	CEA_Homo245-p2	GCGGTGGAGTAATAGTT	2014831
CEA_Homo22-p2	GATGAAATTAAGATACC	378362	CEA_Homo246-p1	AAAAGAAATTCGGATGC	2016858
CEA_Homo23-p1	GGGGACTTGATATTCTA	423461	CEA_Homo246-p2	CAAAATGGTACGGACTG	2016580
CEA_Homo23-p2	GAGACATTAAAGCCCTC	424001	CEA_Homo247-p1	ATCTATCATGCGCCATAC	2019131
CEA_Homo24-p1	AAATGTATGAGCTTGTTAG	423624	CEA_Homo247-p2	GAACTTCCGATTCGTTT	2019825
CEA_Homo24-p2	TTATCCTGCTTATTCTG	424484	CEA_Homo248-p1	TACCCAATGGTCTAAAT	2029175
CEA_Homo25-p1	TTTGCTATGGATAAGAG	425458	CEA_Homo248-p2	AAGTGGGACCCTCAACC	2029694
CEA_Homo25-p2	TAAAATCTACACCTGCT	425827	CEA_Homo249-p1	TTGCCTCTATCTGTGAA	2042755
CEA_Homo26-p1	TGTTTATTGGGAAAATAG	433535	CEA_Homo249-p2	CTTGAAGAACCCTATCC	2043315
CEA_Homo26-p2	CTTTGCCAAAAGTAATT	433993	CEA_Homo250-p1	GGCATAATTCCCACCAC	2043868
CEA_Homo27-p1	GGAAGTAGTTGTAATCA	438844	CEA_Homo250-p2	TACGCAAGCAGGTTTCT	2044514
CEA_Homo27-p2	ACTACGTTCCTTATTTA	439317	CEA_Homo251-p1	TGCATACTGTATTTGCTC	2048198
CEA_Homo28-p1	TATTCTACTATGGGCTTCC	440440	CEA_Homo251-p2	TCAGATTGTTGCGTTAG	2049071
CEA_Homo28-p2	CACCTCCTAATACAAAGAT	440952	CEA_Homo252-p1	TATGAATGCCCTATCTT	2050748
CEA_Homo29-p1	CCCCACTCCTACTACAA	440486	CEA_Homo252-p2	GAGGCTTTAGAGCTTCT	2051319
CEA_Homo29-p2	ATCAATTCTTCCGCTCT	441258	CEA_Homo253-p1	ATTCCAAGCCACATACA	2061971
CEA_Homo30-p1	AATCCTGGCAAGTATGA	443181	CEA_Homo253-p2	AAGGCAGATTTGAGCAGT	2062493
CEA_Homo30-p2	GCTTTAGCGTTGTAGCT	443886	CEA_Homo254-p1	TTATGACGAACCCCTAT	2063963
CEA_Homo31-p1	AATCCTGGCAAGTATGA	443181	CEA_Homo254-p2	ACTTGCCTCAGGATTAT	2064635
CEA_Homo31-p2	GCTTTAGCGTTGTAGCT	443886	CEA_Homo255-p1	CTTTCCCACGATTAGGT	2068349
CEA_Homo32-p1	ACCAGCCTTCAGTGTTG	448916	CEA_Homo255-p2	AGGATAGAAGGAGGTCA	2069114
CEA_Homo32-p2	TTGGCGGTATGAGTTTT	449742	CEA_Homo256-p1	TTAAGGATTTAGCGAAGC	2079665
CEA_Homo33-p1	GAAGGAACAGAACAAGA	451404	CEA_Homo256-p2	TGTAGATTGTGGCGTAG	2080551
CEA_Homo33-p2	GGTAAAGCAGGTAGATA	452055	CEA_Homo257-p1	GTCTTACAAAGCTCCTA	2085533
CEA_Homo34-p1	ACTTCGAGTTACGCTAT	452402	CEA_Homo257-p2	ATAATGCCTAAAGTTGG	2086261
CEA_Homo34-p2	ATGTACTTTGTCACCCT	452866	CEA_Homo258-p1	CCTCCTTTGAACCATTT	2092273
CEA_Homo35-p1	GCAATCTACTTGTCTTA	456928	CEA_Homo258-p2	AAGACTCCCATCCACTA	2093050
CEA_Homo35-p2	GATACAGGAATACCTCT	457432	CEA_Homo259-p1	CATCATCGCAATTAGTA	2094536
CEA_Homo36-p1	GTGCCTGGGGCTGTAAA	460989	CEA_Homo259-p2	AAGCGTTAGATAGGTTA	2095255
CEA_Homo36-p2	TCAAACGCATCATGTCC	461586	CEA_Homo260-p1	TTTCAATAAAGCTCCTG	2096313
CEA_Homo37-p1	TCCCATAAAAGCTATTG	503723	CEA_Homo260-p2	AAAGTTGTTGGGTAATC	2096858
CEA_Homo37-p2	TACATTCAAGCCAAGTT	504190	CEA_Homo261-p1	CCCACAGTTCAAGTAAA	2099664
CEA_Homo38-p1	GCTCAACCGAAATGTTA	513217	CEA_Homo261-p2	GATAGATATGGTGCTGA	2100320
CEA_Homo38-p2	TTGCTTTTCTTTCTCCTTA	513761	CEA_Homo262-p1	GCTTCCTTATTTCCTAC	2113227
CEA_Homo39-p1	ATAGAATATGGCTTTATGG	514086	CEA_Homo262-p2	CTCTTACTTTGTTTTCCA	2114060
CEA_Homo39-p2	ACAACCTCATCCCTCTG	514582	CEA_Homo263-p1	CGCTTTTACTACATACG	2116238
CEA_Homo40-p1	TACACCAACTAAAGCAA	519766	CEA_Homo263-p2	CACAAATACATAACTGCTT	2116830
CEA_Homo40-p2	TACCAAAGCAGTATCAA	520295	CEA_Homo264-p1	ACTTACCAGAAACAATC	2127404
CEA_Homo41-p1	TCTAAGATGTCCGATTT	519783	CEA_Homo264-p2	GTTGATGATTTAGATGG	2128119
CEA_Homo41-p2	TTCTAACACCTTATCCC	520527	CEA_Homo265-p1	CCCCTAGATCCTTTATC	2135274
CEA_Homo42-p1	AGGCAGCTTTAATAAATGG	557841	CEA_Homo265-p2	TGTTGTGGCAGACTTAT	2135755

CEA_Homo42-p2	AGCAACCGCAGGAGTAG	558641	CEA_Homo266-p1	AAACTGTCCGCATCCTT	2149727
CEA_Homo43-p1	TTAATGTAGCGACTCCG	573002	CEA_Homo266-p2	ATTCATGGGCTTTGGTG	2150426
CEA_Homo43-p2	GCTATCTGCCTTTATTGTT	573866	CEA_Homo267-p1	CGCCCTGAAGTGGATTA	2155168
CEA_Homo44-p1	CCGCTTATAGCGACTAAC	587661	CEA_Homo267-p2	GCGGAGGATTTAGATGAA	2155877
CEA_Homo44-p2	CAACCTCACCGTAGGAA	588356	CEA_Homo268-p1	GTGAAACGGGCTTTATT	2158037
CEA_Homo45-p1	AGATTAGCCATTCGTTT	600140	CEA_Homo268-p2	GAGGACCTTATGCAAGAA	2158677
CEA_Homo45-p2	AAGTCTCGCATTCAGTA	601153	CEA_Homo269-p1	TATCTTCCATGTAGTCA	2160681
CEA_Homo46-p1	GTTATGACGAAAGGTGGAG	606779	CEA_Homo269-p2	TAAGTTCAAAGGATGTG	2161626
CEA_Homo46-p2	TTCAAACCCGTAAATGC	607662	CEA_Homo270-p1	TGAAAAGCAAGACCTAA	2181755
CEA_Homo47-p1	TCATATTCTCATGGCTTGT	608768	CEA_Homo270-p2	TGTGAGCAGTATGGAAA	2182547
CEA_Homo47-p2	CCTCTACCGTTTCTTCC	609549	CEA_Homo271-p1	ATGAGCCTGTCTTTGGA	2193176
CEA_Homo48-p1	CGTATGATTTATGGAGCAA	614198	CEA_Homo271-p2	TTAAATTGGGACACCGT	2193631
CEA_Homo48-p2	CGCACAGGCTATCTTTT	614730	CEA_Homo272-p1	CTTAACCTCAATTCCAT	2195985
CEA_Homo49-p1	AAGATGAAGGCTGGTAC	632177	CEA_Homo272-p2	GAGTGCGGTAACATAGA	2196666
CEA_Homo49-p2	TAAAGGTTACTTGTGCC	632622	CEA_Homo273-p1	CTCTAAAATCCTTACCA	2196668
CEA_Homo50-p1	CTGCGGCTAGTGAAGAG	633962	CEA_Homo273-p2	TTCAAGGAAATATGAGA	2197366
CEA_Homo50-p2	AAACCAGCCTTGGAACT	634612	CEA_Homo274-p1	AAACTGAATACCTCGTT	2201132
CEA_Homo51-p1	AAAGAGGGAAGAGTTCA	656228	CEA_Homo274-p2	TTTAGACATTAGGCTTG	2201997
CEA_Homo51-p2	GTATGGGCATAAGCATT	656760	CEA_Homo275-p1	TTCCTTAACATTTGCTA	2201444
CEA_Homo52-p1	GTGGGAAAGAAATGTAA	674872	CEA_Homo275-p2	AAGTGTCTCCAGGTAGT	2202056
CEA_Homo52-p2	AAGACTGCCACAAATAA	675708	CEA_Homo276-p1	CCTTTGACTTCTCCTTT	2201468
CEA_Homo53-p1	AGGAAAAGAGTACAGGA	675544	CEA_Homo276-p2	TTCCGCTGCTTACTATA	2202069
CEA_Homo53-p2	CAAATACAAGAAGCCAC	676091	CEA_Homo277-p1	CTAACTCCACCCATTAC	2205081
CEA_Homo54-p1	TCGTTAGGCCACTTTAT	681406	CEA_Homo277-p2	GACTTAGTTTGGCATCA	2205715
CEA_Homo54-p2	GAGCCCTCTGCATCTTT	682275	CEA_Homo278-p1	AGGAGCAAGCAAAGGAG	2224609
CEA_Homo55-p1	AGAGCTATCCCTCAAAT	693283	CEA_Homo278-p2	ACCAGCGGTTAAGAAGC	2225244
CEA_Homo55-p2	AGAGTTCCCTTTCCTAC	693780	CEA_Homo279-p1	TTGTTGGTGCTGTAAGT	2230661
CEA_Homo56-p1	ATTAGACTTTGGAGCAT	719291	CEA_Homo279-p2	ATCTGTATCTGGGTTTT	2231268
CEA_Homo56-p2	AATGGCTAAGTGTATGG	719958	CEA_Homo280-p1	CATAACGCCAAGACCAT	2240864
CEA_Homo57-p1	AGAATTTGAGCCTACAT	722808	CEA_Homo280-p2	AGCAGCTTTAATTCAGT	2241371
CEA_Homo57-p2	ATTACAGAGGGAACCTG	723487	CEA_Homo281-p1	CCCGCATTACGAACTAT	2246838
CEA_Homo58-p1	AGAATTTGAGCCTACAT	722867	CEA_Homo281-p2	TCAAAGGATGACAACACTG	2247195
CEA_Homo58-p2	ATTACAGAGGGAACCTG	723578	CEA_Homo282-p1	CCAAGTGGCATCAATAT	2249601
CEA_Homo59-p1	GCAGAAGCAAATAGAAC	750629	CEA_Homo282-p2	TCAAGAGGTGGAGAAGA	2250557
CEA_Homo59-p2	CAGAAGAACAATGGAAT	751176	CEA_Homo283-p1	GCATCCAAGTCCAAATA	2258252
CEA_Homo60-p1	CCCATTGGTTGAAATAC	753107	CEA_Homo283-p2	GAAACTCTAAACCAAGA	2258852
CEA_Homo60-p2	ATTCTGGTTCAGGCACT	753718	CEA_Homo284-p1	TGACTGCGCTAGTGATA	2352138
CEA_Homo61-p1	ATAGATTACGAATGTATGG	760777	CEA_Homo284-p2	GTGCCTTCTGAAATAAA	2353016
CEA_Homo61-p2	TGCATTAGCCTCAATAA	761323	CEA_Homo285-p1	AAAAGGCTCAAACTCCA	2353442
CEA_Homo62-p1	ATGGTACAAGCCACAAA	766625	CEA_Homo285-p2	AATTTAGCAGCAATACC	2354252
CEA_Homo62-p2	TGCGTAATCCGAGTTAG	767638	CEA_Homo286-p1	ACTGAATTGCAGCGTAA	2358824
CEA_Homo63-p1	GAAGATGCAGCTAAAGT	783491	CEA_Homo286-p2	ATTGGGTGTTGAGAAGG	2359356
CEA_Homo63-p2	ATGGTCTCCCAAGTTTT	784148	CEA_Homo287-p1	GGGAATCTCCAAACTCA	2363176
CEA_Homo64-p1	AAAGGGATGATTGGTGT	789352	CEA_Homo287-p2	GAAGAGGGTGATGAACG	2363861

CEA_Homo64-p2	GCTTGAATATCTGGGTC	790058	CEA_Homo288-p1	TATACCGCAAGTGTTAC	2379805
CEA_Homo65-p1	GGGAAGATGAAGGTAGA	789640	CEA_Homo288-p2	CTGGTCTTATGGCAGAT	2380758
CEA_Homo65-p2	GCTTGAATATCTGGGTC	790136	CEA_Homo289-p1	TTTAAGTGCCTCTACTC	2392832
CEA_Homo66-p1	GATTTTACTATGAGCGG	798001	CEA_Homo289-p2	TCCTATACTCACTACGG	2393888
CEA_Homo66-p2	TTGTGAACGTGAACAAC	798500	CEA_Homo290-p1	GGCATATTAGCTGCTTT	2397812
CEA_Homo67-p1	AACAACGACGCTGACTA	805674	CEA_Homo290-p2	ACAGGTGGGCTCGTTAG	2398460
CEA_Homo67-p2	TTTCTTTCGGAAGGTTA	807370	CEA_Homo291-p1	AGCATTCTAAGGGTAAG	2421462
CEA_Homo68-p1	TGATAGTAGCAGGATTG	810552	CEA_Homo291-p2	AAGGGATGAGTGTATTG	2422328
CEA_Homo68-p2	TACTTCACTAGGCACAT	813482	CEA_Homo292-p1	TGCTTCAATTAGGGTAA	2430191
CEA_Homo69-p1	TTCCGCAATACCAACTC	831868	CEA_Homo292-p2	TTCAGAAGGCTCAGAAA	2431087
CEA_Homo69-p2	AAAAGGCACAAAACTGT	832510	CEA_Homo293-p1	ATCTAAGTCATCCGATA	2437971
CEA_Homo70-p1	TGCTTCAAGTAGGTGGA	831889	CEA_Homo293-p2	TAATAGGAAAAGCGTTG	2437662
CEA_Homo70-p2	TGAAACGCCTACAAACA	832738	CEA_Homo294-p1	TTTACAGCACTTCTCAG	2456842
CEA_Homo71-p1	CCAAATAGAGCCTGGAA	872834	CEA_Homo294-p2	AGTGAAGGAGCAATCTA	2457822
CEA_Homo71-p2	TGCAAAGAGGTGGAAAC	873482	CEA_Homo295-p1	GGTTTAGGAAATGTTGT	2463883
CEA_Homo72-p1	CATTCCAACACCAGCAA	873924	CEA_Homo295-p2	AAAGTTGGCAGATAGTA	2464436
CEA_Homo72-p2	TGACGATTAGGCAGACA	874556	CEA_Homo296-p1	TTTCTTCCCTTTGTTTC	2466595
CEA_Homo73-p1	AGCCTAAAGAGTATCCA	878229	CEA_Homo296-p2	GTATGGTTCATCCCAAT	2467386
CEA_Homo73-p2	TCTATTCTATGCCAAGT	879019	CEA_Homo297-p1	TAGGCAATACTACAACA	2476454
CEA_Homo74-p1	GTAGAACACGACTTTGGAA	882175	CEA_Homo297-p2	CACCGTCATAAACAGGA	2477554
CEA_Homo74-p2	CTTGATTACCGGACACC	882821	CEA_Homo298-p1	TGTCATTATGCCACCTA	2481492
CEA_Homo75-p1	TACTTTAACATTGACCCAC	890670	CEA_Homo298-p2	TGGGATAATAGCAAGAG	2482040
CEA_Homo75-p2	GAATACGACGAAGCAAA	891230	CEA_Homo299-p1	AATCAATCCGCCGTTAT	2481781
CEA_Homo76-p1	AGTTGGCTTGAAGAGGA	904654	CEA_Homo299-p2	TACTTCAATGGCCGTCT	2482460
CEA_Homo76-p2	ACAAAGGATGGAGGAAT	905573	CEA_Homo300-p1	TTCCTCGCAAGATTTAC	2484926
CEA_Homo77-p1	ATATTGCATATGTAAGG	932713	CEA_Homo300-p2	AAGCATCACAACCGTAT	2485554
CEA_Homo77-p2	AACCATTTAACTGTACC	933391	CEA_Homo301-p1	GCTTCGCATTTACATTA	2486004
CEA_Homo78-p1	ATAGAAGCCATACCTGA	942952	CEA_Homo301-p2	CACCATCTACGCCTATC	2486632
CEA_Homo78-p2	ACTAAGTCTTTCGATGC	943723	CEA_Homo302-p1	CTAAGTCCATCCCTGTA	2490767
CEA_Homo79-p1	GTAGTAGGAAGTGGAGT	946320	CEA_Homo302-p2	ATGGCTGATGAGAAGAT	2491414
CEA_Homo79-p2	TCTTATCTGTATTTGGA	946820	CEA_Homo303-p1	CGAATAAATCTTCTCATC	2491416
CEA_Homo80-p1	TATGTCCCCTTATTGAT	946967	CEA_Homo303-p2	GTAAAGGTTGTTTGTCT	2492066
CEA_Homo80-p2	GGAACTTTTCTAACCCT	948020	CEA_Homo304-p1	TTTTCTTCTATAAGCTAG	2510406
CEA_Homo81-p1	TACTGATAGCATCTTGG	959746	CEA_Homo304-p2	TCGGATAATAAATGGTA	2510927
CEA_Homo81-p2	TATCACTGCTTCCTATT	960278	CEA_Homo305-p1	CGACAAATACGGAAGAC	2518578
CEA_Homo82-p1	AGGTTGCTCTTTGGTAC	961955	CEA_Homo305-p2	CTGTTATTGGCAGCATA	2519282
CEA_Homo82-p2	TTGCTCCTGTTCTTTCT	962795	CEA_Homo306-p1	CTATCGGTTTTCGTCAC	2526175
CEA_Homo83-p1	AGGGTATCCTTAGTGGT	973413	CEA_Homo306-p2	GAGGCTATTCTTGGAGT	2526896
CEA_Homo83-p2	TTGATCCGGTAAATCTC	973930	CEA_Homo307-p1	AACTCAATTACACGCTC	2527175
CEA_Homo84-p1	ACCCTGACTCAGGAAGT	985070	CEA_Homo307-p2	TATAAGCTAGGGGAAAG	2527932
CEA_Homo84-p2	GGTTCTTAGCCTCATTT	985559	CEA_Homo308-p1	ACTTCCCCTTTATTCCA	2528079
CEA_Homo85-p1	GAGGCATCATTAGGAGC	1003782	CEA_Homo308-p2	ACAGCGTTGTTTTCCTT	2528654
CEA_Homo85-p2	ACCTTTGCCCTATCTTC	1004358	CEA_Homo309-p1	TTGCCCATAAGCAGAAA	2530326
CEA_Homo86-p1	TTGAAACGGAAGGAGTG	1011434	CEA_Homo309-p2	GTAGTCCAAGTCCCTCA	2530901

CEA_Homo86-p2	AGAACCCATACAGAGCA	1012045	CEA_Homo310-p1	AAAACTATGGCTAATGG	2549259
CEA_Homo87-p1	TGTCAAAGGCGAGATAC	1036023	CEA_Homo310-p2	ACTACTTGTCCGAATGA	2549878
CEA_Homo87-p2	GGTAAGCGCAGAATAAA	1036739	CEA_Homo311-p1	ATTCTTCAGCCTTATCC	2551614
CEA_Homo88-p1	GGGAGGAACAAAGGATA	1038894	CEA_Homo311-p2	AGTTCCCTTGTCTTCTA	2552282
CEA_Homo88-p2	ACCGCAGATAACTGAATG	1039594	CEA_Homo312-p1	TTAGTAAGCGTTTCGTG	2553342
CEA_Homo89-p1	GATGGAAGATAGTAGGT	1040828	CEA_Homo312-p2	TAAGGCTACTGGGCAAT	2554152
CEA_Homo89-p2	CTTAGACGAATCGTTAG	1041430	CEA_Homo313-p1	GCTGGATTTGTTCTTGG	2555226
CEA_Homo90-p1	CTAGTAGTCCAGCAACA	1047468	CEA_Homo313-p2	TTATTATGGGTGGCTTT	2555986
CEA_Homo90-p2	CTCCCTCTGAAGATTGT	1048317	CEA_Homo314-p1	TCTCCTCTACAGCCATT	2563741
CEA_Homo91-p1	CCAGGAGATGTAAATGG	1049122	CEA_Homo314-p2	ATCATCACATTTACCCA	2564467
CEA_Homo91-p2	CTGTCTGTCCACCACTT	1049820	CEA_Homo315-p1	AAAAGCCGCCTTGGGTA	2564414
CEA_Homo92-p1	AGGCAATCCTGGAGACT	1059914	CEA_Homo315-p2	TCACCGCAAGCGTAGAT	2565133
CEA_Homo92-p2	CCTTGGCTTTGAACTGA	1060758	CEA_Homo316-p1	AACAGCCTTTAACTGCA	2568029
CEA_Homo93-p1	AACCCAACACCAGATGA	1065136	CEA_Homo316-p2	TTCACCATTATCCCATC	2568758
CEA_Homo93-p2	TATAACGCAGCAGCCAC	1065727	CEA_Homo317-p1	TTTCATAGTTTCCCTCC	2571930
CEA_Homo94-p1	GCAGAATTGGCGGATAA	1080050	CEA_Homo317-p2	AAGTCAAATGGCTTCTC	2572774
CEA_Homo94-p2	AAGAGCAGCCGTTCCTA	1080518	CEA_Homo318-p1	TTCCTTTACATGGGTAC	2575172
CEA_Homo95-p1	ATAGGATTGAAGGAACT	1088743	CEA_Homo318-p2	ACATTTGGCTTTGAGTA	2575857
CEA_Homo95-p2	ATTAAGGTTACTATTCC	1089388	CEA_Homo319-p1	ATTCCATCTTCTGATTC	2577239
CEA_Homo96-p1	CATCGCTTCCGTTATTT	1100782	CEA_Homo319-p2	TAAGTAAAGAGCAGGTT	2577806
CEA_Homo96-p2	TGGTTTCGGGTATTAGA	1101536	CEA_Homo320-p1	GCTATGGGCTGTAAATT	2582816
CEA_Homo97-p1	GACCGGGATGGACTGAC	1109282	CEA_Homo320-p2	AGTATGGAGACGGTGAA	2583429
CEA_Homo97-p2	CTGCTGCTTCATACTCTTG	1109897	CEA_Homo321-p1	TGATGGTCACGGAGAAG	2583701
CEA_Homo98-p1	CTTCGTTTGGTACAGAT	1111965	CEA_Homo321-p2	TTGAGTTAAGCCAAAGG	2584411
CEA_Homo98-p2	AGAATTCCTTTTAATCG	1112511	CEA_Homo322-p1	TTCCCTCAGTAACCTCA	2588381
CEA_Homo99-p1	GCAAACATTTAGAAGTT	1113322	CEA_Homo322-p2	TTGTCGCCCAGTAAAAC	2589061
CEA_Homo99-p2	GTAGTTATCTGTGGGAA	1113991	CEA_Homo323-p1	TATAGAAAAGCGACAGA	2598090
CEA_Homo100-p1	TGAAGCAGCAGATAAGA	1117896	CEA_Homo323-p2	GATTCAAATAGTCAAGC	2598938
CEA_Homo100-p2	CCGCTCCACTATTTACAG	1118358	CEA_Homo324-p1	CAATATCCCTCTTATCC	2602287
CEA_Homo101-p1	ACCTACATCAGGACCTT	1135987	CEA_Homo324-p2	TTTGTAGTGGCTTCATT	2602903
CEA_Homo101-p2	TTACATCATAACCAGCC	1136614	CEA_Homo325-p1	AAAATAGAGGCATAGGA	2627511
CEA_Homo102-p1	TATCGTTGGTATCTTAC	1139999	CEA_Homo325-p2	TTTGGATAGTGTAGGGT	2628079
CEA_Homo102-p2	ATCTCCTTTTCTGTAGT	1140537	CEA_Homo326-p1	GCCTCAATTCTTAGTCC	2639768
CEA_Homo103-p1	GATGTGCTCAAAAGGTG	1143402	CEA_Homo326-p2	GAGGTGTTACTCCAGCA	2640698
CEA_Homo103-p2	GTTCAAAATCGTCGTAA	1144010	CEA_Homo327-p1	GCCGTATACTGAACTGC	2650969
CEA_Homo104-p1	AAGACAAGCATAATAACC	1148150	CEA_Homo327-p2	AATTTGTCCTTTCGGAG	2651957
CEA_Homo104-p2	GTATCGCAATATCAGAATA	1149126	CEA_Homo328-p1	AAACTAAACCCTGTGGC	2684229
CEA_Homo105-p1	TCGTATTTAAGAGGCAGAA	1151226	CEA_Homo328-p2	GAAGATAGATTGCTCCC	2685154
CEA_Homo105-p2	TGAAGAAACAGGAGCAT	1152067	CEA_Homo329-p1	TGGAGTGAATTTAGCAT	2699337
CEA_Homo106-p1	TTACCACATGATAATATG	1157190	CEA_Homo329-p2	TGTTTTAGGGTTAGCAA	2699866
CEA_Homo106-p2	TACTGCTCTAGCAACTA	1157818	CEA_Homo330-p1	TGTTACCATTGCAGTAT	2714146
CEA_Homo107-p1	CTATTGTCGCTGCGTGTA	1160306	CEA_Homo330-p2	AGGAGTAGGCTTTAGTT	2714880
CEA_Homo107-p2	ATCTGTAAGGGTTGGTG	1161218	CEA_Homo331-p1	CTATTACTCCCCATCAA	2721850
CEA_Homo108-p1	TATTTGTGGGACGTTGA	1167604	CEA_Homo331-p2	CCGATATTATACGAAAG	2722670

CEA_Homo108-p2	AAGCGCACTCCTCTTAT	1168442	CEA_Homo332-p1	GAGCGAATCCAATACAT	2729826
CEA_Homo109-p1	TTGTATCAGCATCGTTT	1173355	CEA_Homo332-p2	AAACAACCCTAACAGAA	2730562
CEA_Homo109-p2	CTTCCCCTTAGATTGTT	1173872	CEA_Homo333-p1	TCGTTGTCGCTTCACTC	2738771
CEA_Homo110-p1	TGTGGTTCAAGAAACTT	1174562	CEA_Homo333-p2	GACGCTTCAAGCAATAC	2739490
CEA_Homo110-p2	ATCCAGAATAACTGCTG	1175137	CEA_Homo334-p1	CAAGCATAAGCCCACTG	2741633
CEA_Homo111-p1	AGGGACTTGAGTTTGAT	1175858	CEA_Homo334-p2	CGTTCATGTTCACCACC	2742269
CEA_Homo111-p2	TGTATGCTGCACGACTA	1176461	CEA_Homo335-p1	TTCAGGATAAGCAACTG	2752206
CEA_Homo112-p1	TTATTACGGTAGTTGTTTC	1186512	CEA_Homo335-p2	GCTAATAAAGAACCAAAG	2752845
CEA_Homo112-p2	GGTTTGATTGTATTTGG	1187036	CEA_Homo336-p1	TTATCAAGAACACCAGCAT	2757191
CEA_Homo113-p1	AATAAAGGTGTAAAACTGG	1195855	CEA_Homo336-p2	CAGGAGGAAATACCTACAA	2757887
CEA_Homo113-p2	CTACCCGCCATACCAAC	1195383	CEA_Homo337-p1	TATCCTCCTTCAGTGTC	2757505
CEA_Homo114-p1	GAAAAGTGGGGAAAGTA	1217311	CEA_Homo337-p2	AAACATGGGAGTAGTTG	2758369
CEA_Homo114-p2	TATAACCTCGTTGTCGT	1217883	CEA_Homo338-p1	ATATGCTCCTCCTCCCC	2776942
CEA_Homo115-p1	CCCCACTTATAGTGTCC	1224111	CEA_Homo338-p2	TCGGCATCAGGATACTCAG	2777549
CEA_Homo115-p2	AATAATCTCCCAACTCC	1225055	CEA_Homo339-p1	ATCCCCTAAATGTATGA	2780657
CEA_Homo116-p1	ATTTAGGAAAAGCAACTAC	1239692	CEA_Homo339-p2	AATTGTGCTAAGTTGCT	2781398
CEA_Homo116-p2	CATTTATTGACTGACCG	1240676	CEA_Homo340-p1	AAACATCTTTCCCTCCA	2795664
CEA_Homo117-p1	TAAATGGATGGGCAAAC	1242039	CEA_Homo340-p2	ACCTAAGTTCGCCACAG	2796595
CEA_Homo117-p2	AAATCCTCACCCTAACA	1242984	CEA_Homo341-p1	GGAGGTCTTTATTTATG	2801624
CEA_Homo118-p1	GGGATTCTTTAGAGCCA	1255844	CEA_Homo341-p2	GTATCTTGTATTACCTTGA	2802107
CEA_Homo118-p2	AAACCTTATCGCCTTCC	1256605	CEA_Homo342-p1	TAAATGCAGCGAAGAAA	2802424
CEA_Homo119-p1	GCTTCAAGGGCAATAGA	1259745	CEA_Homo342-p2	ATAGCGTTGTCGGGAAG	2803017
CEA_Homo119-p2	TAGCCAATAGCATAGTT	1260480	CEA_Homo343-p1	TATTGCTCCATTCCTAT	2804842
CEA_Homo120-p1	AGTTGGTATTTGCTGAG	1279751	CEA_Homo343-p2	GTTTCTTTCCGTATCTT	2805624
CEA_Homo120-p2	TTGGGACATAGTATTCTTA	1280422	CEA_Homo344-p1	TGTCTTTTGAAAGGGAT	2823633
CEA_Homo121-p1	GCATTAAGATAATCGAG	1293955	CEA_Homo344-p2	TGAATATGGCAGGATGT	2824138
CEA_Homo121-p2	GTGTTTTACAAAAGGGT	1294695	CEA_Homo345-p1	ACCGCTCTTAGTAGTCT	2828179
CEA_Homo122-p1	TCATTTAACCGATAGAG	1307592	CEA_Homo345-p2	TTCTACGTCCATAAAGT	2828894
CEA_Homo122-p2	GTTTCCGTTCATACTTT	1308344	CEA_Homo346-p1	TTTCTTGTGGCAAACAC	2839877
CEA_Homo123-p1	TGAGATTATTGAAACGGAT	1332221	CEA_Homo346-p2	AGCAGTTACATGGCGTA	2840562
CEA_Homo123-p2	GCATTAGGGTACAGACT	1333093	CEA_Homo347-p1	TTCCCATGATAGGTATT	2849180
CEA_Homo124-p1	AATGAAGCGTATAGTATGG	1333270	CEA_Homo347-p2	TTTATGTGGTGGATTGT	2849810
CEA_Homo124-p2	TAGGCATTATTACAGATGG	1333943	CEA_Homo348-p1	TTTTAGATACGCCTCAG	2849527
CEA_Homo125-p1	AGAAAAGGAAGGTATTA	1334716	CEA_Homo348-p2	TTGGCTTGTATGTAGGA	2850305
CEA_Homo125-p2	TTACAAGCCTGCTCACT	1335402	CEA_Homo349-p1	GTTACCTTCGCACTCCA	2857031
CEA_Homo126-p1	TCTAATGTTATTGGGAAAG	1336382	CEA_Homo349-p2	AGGCTGCTTTATTTCGT	2857622
CEA_Homo126-p2	ATCTGCACTACAGGTTGAT	1337296	CEA_Homo350-p1	AACCTCCACAGCTCCAT	2861274
CEA_Homo127-p1	AAGTTACCCAAATCCAG	1347191	CEA_Homo350-p2	TTAAGTGAACGCTCAAA	2862030
CEA_Homo127-p2	TTTCTCCCTTCTTCCAC	1348004	CEA_Homo351-p1	AGCTACTTTAGGGTTAA	2884670
CEA_Homo128-p1	GAAACAAGGTAAAAGCC	1349749	CEA_Homo351-p2	CATAGAATAAATGGAGG	2885391
CEA_Homo128-p2	CGAAAATAATCCCAATC	1350449	CEA_Homo352-p1	GACACCATAACCGAAAC	2886428
CEA_Homo129-p1	TCAATCGTAACTCTTTG	1358842	CEA_Homo352-p2	AAGGTGGCATTGAAGTA	2887328
CEA_Homo129-p2	ACTAATCACCACCTTTT	1359449	CEA_Homo353-p1	CCTCACTTTAATTATTAG	2890281
CEA_Homo130-p1	TGTAGAGTATCGGAAAT	1361030	CEA_Homo353-p2	TTCTAAGTCTTGCGTTT	2890867

CEA_Homo130-p2	TAGTGGGTTATCATCAA	1361828	CEA_Homo354-p1	AAATGAATGGAACCCTA	2904390
CEA_Homo131-p1	ACAAAGACGATTTCTATTG	1362308	CEA_Homo354-p2	TCTCCTTGAATGCAGAG	2905042
CEA_Homo131-p2	ATGGATCAGATGGTGGT	1363022	CEA_Homo355-p1	AGTAATCTTATGGGAGGTA	2910213
CEA_Homo132-p1	TACCCATACCAAAAGAA	1365148	CEA_Homo355-p2	GTCTCATATCCGTGGTC	2910914
CEA_Homo132-p2	CATTAAGTGCAATTACATC	1366238	CEA_Homo356-p1	CTGTAGTTGGATCAGCA	2913762
CEA_Homo133-p1	AAGCAACAGGAGATTTT	1369642	CEA_Homo356-p2	GAATGAAACTTGGAATG	2914357
CEA_Homo133-p2	ATGGTGGTACTATAACTAA	1370372	CEA_Homo357-p1	TCCTTGTAACCGCTGAG	2919085
CEA_Homo134-p1	TCAAATACTTGGCTTAC	1374481	CEA_Homo357-p2	AATCGCTGTTGTTGAGG	2919986
CEA_Homo134-p2	ATAGCTTCCTACCTTTC	1375137	CEA_Homo358-p1	AATTCCTCCTAGTAAAT	2919896
CEA_Homo135-p1	TCCAAAGCCGAGTAATG	1381262	CEA_Homo358-p2	AAGTTTCCCTAGAGTTC	2920480
CEA_Homo135-p2	CGGATGGCAAACAAGAA	1381748	CEA_Homo359-p1	GAGGAGCACAAGTAGGC	2922136
CEA_Homo136-p1	ATGCTAATGCAGGAAAG	1394416	CEA_Homo359-p2	AGATCCAGCGTGTTATT	2922831
CEA_Homo136-p2	GAACCGCACTGTAAATG	1395082	CEA_Homo360-p1	TCCTCAGTTGGCTTTCC	2925799
CEA_Homo137-p1	GCAGGAAAGGCATTTAG	1394420	CEA_Homo360-p2	ATCAATTCCGTTTCGTC	2926385
CEA_Homo137-p2	TCCCATCAAGCACCATA	1395121	CEA_Homo361-p1	CTCTTTGTAGCAATCAC	2960844
CEA_Homo138-p1	GGAATACAGGCAATAGG	1404919	CEA_Homo361-p2	TTGTTGTACCAAGTGAA	2961485
CEA_Homo138-p2	AATTCATAGCCTTCGTT	1405852	CEA_Homo362-p1	AGAATCCGGCGCTAATA	2964618
CEA_Homo139-p1	ATAAGTGCAGCTTCCAT	1413244	CEA_Homo362-p2	GAGGAAAGGCTGGTAAT	2965490
CEA_Homo139-p2	TAGGCTTTTGACTTTCG	1413884	CEA_Homo363-p1	AATCTCAAATCCTCCCA	2974838
CEA_Homo140-p1	TGCGATAAGATGATAAATG	1416557	CEA_Homo363-p2	GAAAACAGCCAGAAATAA	2975742
CEA_Homo140-p2	TTCGGTTTTCTCACAGT	1417274	CEA_Homo364-p1	CCAAACTACATTTCCCTT	2993116
CEA_Homo141-p1	CCTTGGGTTAAAGTAGC	1418435	CEA_Homo364-p2	TGTTTCGGTAAATCAGC	2994164
CEA_Homo141-p2	AGTTCCCTTAGCCTCAT	1419252	CEA_Homo365-p1	CTCCTCCAGATTTACCT	2995377
CEA_Homo142-p1	AAGCAGTAAAGGAAGCT	1446247	CEA_Homo365-p2	ATTAGCAGGACTCGTTG	2995972
CEA_Homo142-p2	CTCCCAATAATAGGTTT	1446830	CEA_Homo366-p1	CGCTTTTATACTATGTC	2997785
CEA_Homo143-p1	GAATACGATAAGGATAT	1451968	CEA_Homo366-p2	GAGTGATTTTGTATGGC	2998487
CEA_Homo143-p2	GACTAAACCTGTAAGCC	1452591	CEA_Homo367-p1	TACGGTTCTTTCATTAC	3004838
CEA_Homo144-p1	AGGATTTATTCTCGCAGTA	1456910	CEA_Homo367-p2	TATGGTTCAGCAGGAGT	3005526
CEA_Homo144-p2	CTTTGGTGGAGCTTTGT	1457601	CEA_Homo368-p1	GATCCCGTATAACCTGT	3009333
CEA_Homo145-p1	GGAAGTGGGAATTGTTC	1458414	CEA_Homo368-p2	TGTTCCATTCTCACCTA	3009936
CEA_Homo145-p2	GTAAGCATCGGCATAGA	1458933	CEA_Homo369-p1	ATTCCTGGCACCTTATT	3012192
CEA_Homo146-p1	TATACTCGGTGCTGGTG	1466303	CEA_Homo369-p2	ATTCGGCTGTGGTAGTT	3012796
CEA_Homo146-p2	TTGGACGTAGTTAAGGTGA	1467000	CEA_Homo370-p1	TTACTATCGGCAACCCA	3017442
CEA_Homo147-p1	AAGAAAAGTAAGCCTATAG	1468126	CEA_Homo370-p2	ACAGACCAAAGGACCAC	3018186
CEA_Homo147-p2	ATCCACTGCAACAACTA	1468685	CEA_Homo371-p1	TATTGCCTGTACTCCAC	3024220
CEA_Homo148-p1	AGCATCTTTACCCATTT	1478228	CEA_Homo371-p2	GACCAAATAGACCACAT	3024995
CEA_Homo148-p2	GAGGACACGCTTTATTC	1478882	CEA_Homo372-p1	CTCCACCTGTTACGAAT	3026545
CEA_Homo149-p1	TAATAAACGGACTTACAGC	1489141	CEA_Homo372-p2	AAGGCTGATTACGAGAA	3027073
CEA_Homo149-p2	ATCCAGGATGACCAGAC	1490306	CEA_Homo373-p1	CTCGAATTAGCCGAAGA	3031161
CEA_Homo150-p1	GGCAAAGTCTGGTCATC	1490300	CEA_Homo373-p2	ACAAGAGGAGCGGTAGA	3031753
CEA_Homo150-p2	CAGCAGAGCCTCCTATT	1491349	CEA_Homo374-p1	TAAAATGCCGGAAACTG	3041644
CEA_Homo151-p1	GCACCATCAAACAAGAC	1491371	CEA_Homo374-p2	GACTGGTCCAATGAAGA	3042554
CEA_Homo151-p2	AGTAGCTCCATGCGAAA	1492335	CEA_Homo375-p1	TTCAACACTTGGGAACT	3054535
CEA_Homo152-p1	ATAAGAGCCAGAGTTGC	1492037	CEA_Homo375-p2	AAAGAATACGCATCAGC	3055334

CEA_Homo152-p2	CTGATAACCCGCTAAAA	1492726	CEA_Homo376-p1	GATTCTGAAAGCACCCT	3057837
CEA_Homo153-p1	GGGGTAAATATGGTTTC	1492560	CEA_Homo376-p2	CAGCCTTTGACTACACCTA	3058738
CEA_Homo153-p2	CTAATGCTCCCTCAAGT	1493553	CEA_Homo377-p1	ACAAGGAGGAAGTGTTA	3060150
CEA_Homo154-p1	GCTTTAAGGCATCAGGG	1494469	CEA_Homo377-p2	CTATGTTTGGAGGAGAA	3061047
CEA_Homo154-p2	CTTCCGAATCAAGCCAC	1495377	CEA_Homo378-p1	TGCTGCTGTAATGAGTC	3083555
CEA_Homo155-p1	TTATGGAATCAGAACCT	1494840	CEA_Homo378-p2	ATGTATTTGTGGGAAGT	3084029
CEA_Homo155-p2	AACAGTAGCAGAAGCAG	1495573	CEA_Homo379-p1	CATACATAAGTTCCGTG	3126216
CEA_Homo156-p1	AAATCGGTGTCAGGTAA	1495045	CEA_Homo379-p2	GTTTTAGAATGTGAGCC	3127100
CEA_Homo156-p2	GAAATAGCATCGCAAAC	1495771	CEA_Homo380-p1	GAATCCTATTTCGATTTAC	3130984
CEA_Homo157-p1	TGCTGCTTCTGCTACTG	1495539	CEA_Homo380-p2	CTCCAGCATTTTATTAGTT	3131625
CEA_Homo157-p2	TACTTGCCGAATCTTTT	1496211	CEA_Homo381-p1	TAAGCACAGCACCTACA	3135703
CEA_Homo158-p1	ATGGAGGTTGCATAAGT	1496287	CEA_Homo381-p2	AGGATAGACTCGGAAGG	3136472
CEA_Homo158-p2	TAGGAGCTACAGGAATT	1497263	CEA_Homo382-p1	TTGTCAGGGAACAAAGA	3144804
CEA_Homo159-p1	GTATTGGCTCTATTCAT	1496947	CEA_Homo382-p2	AGTTAGGCAAAGGTGAA	3145648
CEA_Homo159-p2	TATGTTCTTAGCACCAG	1497760	CEA_Homo383-p1	GCTTTCAACCCTTCTTT	3168583
CEA_Homo160-p1	CAGCGGAGAAGGTAAAT	1497670	CEA_Homo383-p2	AATATGCAGGCTAGTGG	3169308
CEA_Homo160-p2	TTGGGTGTAACAAATAAGC	1498623	CEA_Homo384-p1	AGTCCGCCATACTCTAT	3177650
CEA_Homo161-p1	GTTTCCAGCAGCCATCA	1498492	CEA_Homo384-p2	ATTTGAATGTGGGTGAT	3178373
CEA_Homo161-p2	TTCCAGCGTCAACATCA	1499489	CEA_Homo385-p1	CCTTTACCTGCTTTTCA	3180515
CEA_Homo162-p1	TAGGTGACGGTGTAGCA	1499161	CEA_Homo385-p2	TGGGATAGATGATTTGC	3181173
CEA_Homo162-p2	TTTGAACGTGGAATAGG	1500328	CEA_Homo386-p1	TTAACCTCCCATGTATT	3193383
CEA_Homo163-p1	GCCTACCTCATCTTCTG	1499953	CEA_Homo386-p2	GTTGCTACTTCCCACTA	3194116
CEA_Homo163-p2	TATGCTTCTTCTAATACTC	1500742	CEA_Homo387-p1	CTCACAAAATCTCCACCAG	3194649
CEA_Homo164-p1	GTCGCCAGTTCTTCCTT	1500848	CEA_Homo387-p2	TTCCAAAACCAGTAAAATG	3195226
CEA_Homo164-p2	GATTCCCTTGAATACGC	1501973	CEA_Homo388-p1	TCCTAGCACCAACGACA	3199574
CEA_Homo165-p1	GATAAAGAGGAGCAAAC	1501765	CEA_Homo388-p2	TTGGAGCAAATGGAAGA	3200522
CEA_Homo165-p2	ATGGCATTACTAGAAGA	1503132	CEA_Homo389-p1	TATGTCCCTCCCTTTAA	3208693
CEA_Homo166-p1	ACTTGGTTTACTTTGTTC	1502984	CEA_Homo389-p2	AATTCCTCCAAATCCTG	3209327
CEA_Homo166-p2	CCTTAGTTATTCCTTTTC	1504027	CEA_Homo390-p1	AATGGAATCCGAACGTG	3212004
CEA_Homo167-p1	ACAAGCAGCTAGATTAA	1503934	CEA_Homo390-p2	TCATTTGCTCCCACCAC	3212694
CEA_Homo167-p2	TAGCCATTGAAGTGTAA	1505220	CEA_Homo391-p1	AAGGACGGTATGCTGTT	3223972
CEA_Homo168-p1	GGGTTGACAAATAATAGG	1505039	CEA_Homo391-p2	AAGGCGGAGTTGTTAGT	3224572
CEA_Homo168-p2	ATTACTCCTCCACTCCC	1506028	CEA_Homo392-p1	CAAAACGACATTGTAAAG	3242531
CEA_Homo169-p1	AAACGGCAGTATCTCCA	1505912	CEA_Homo392-p2	TGAAAGGAGTATAGTAGAG	3243176
CEA_Homo169-p2	TAAAGCATTTCCAGCAT	1507383	CEA_Homo393-p1	ATACTGTCCTATCGTTA	3251883
CEA_Homo170-p1	TATTGAGCCTATGATTC	1507075	CEA_Homo393-p2	TTTTATTGATGATGTGG	3252546
CEA_Homo170-p2	GACCTCTGTTCCGTTAG	1508070	CEA_Homo394-p1	TTATGGGAACCTGTATG	3293011
CEA_Homo171-p1	TTTTGAAGCCCACCTCT	1530112	CEA_Homo394-p2	AAAGTATGGAAAGCAGT	3293586
CEA_Homo171-p2	GTCCGATTTCCAACCTTT	1530762	CEA_Homo395-p1	TTTGGCTGGTATGACTA	3302227
CEA_Homo172-p1	CTTTCGATTAAATATCCTG	1534056	CEA_Homo395-p2	ATTTGATATTGCTGGTG	3302911
CEA_Homo172-p2	TCAAAAGTAGTTGCTCC	1534596	CEA_Homo396-p1	ATTTCATACTCGCTGTC	3321183
CEA_Homo173-p1	ATAACCAAGCTAACGAA	1554712	CEA_Homo396-p2	GAGATAATAATCGTGGC	3321879
CEA_Homo173-p2	TCATCACTTTTAGGAGG	1555445	CEA_Homo397-p1	ATCGCATTATGTTTTCC	3331147
CEA_Homo174-p1	TTACCTATAATGAGCCTAG	1565450	CEA_Homo397-p2	TTAACCGATAGGTTGAA	3331807

CEA_Homo174-p2	TCAAGTACCAGCGTTTC	1566074	CEA_Homo398-p1	CTTGACCGTTTACACCC	3340766
CEA_Homo175-p1	CAACATTCACCCGTCTT	1585130	CEA_Homo398-p2	GCTAATACAGCCTACTTTT	3341501
CEA_Homo175-p2	ATTTCCCAAGGTTCACA	1586022	CEA_Homo399-p1	CAACCAAACTTCCCACA	3353076
CEA_Homo176-p1	TCCTCCATTATTCTCAC	1588653	CEA_Homo399-p2	TCCTATTTCCGCTACCA	3353583
CEA_Homo176-p2	TTCACCAGCATACTCTA	1589480	CEA_Homo400-p1	AAGACATTTATCCCTAC	3353466
CEA_Homo177-p1	ATTTACCATCACATACT	1590774	CEA_Homo400-p2	TAGTTATTGTCGCTGAT	3354043
CEA_Homo177-p2	TTTACAAGATTACAAGC	1591466	CEA_Homo401-p1	CCCCAAATACTGCTCCA	3358926
CEA_Homo178-p1	AGGTTGGCAATGCTGTA	1597304	CEA_Homo401-p2	ATACAAGGCACAAATCA	3359716
CEA_Homo178-p2	GCACCTAAGGATGTCCC	1597989	CEA_Homo402-p1	CTCTTCAGGGATTTGTG	3367488
CEA_Homo179-p1	ACAACATTGGGTAGCAT	1624872	CEA_Homo402-p2	AGAGGGTAAACATTGAG	3368068
CEA_Homo179-p2	AAGTTGAACCAAATCCC	1625393	CEA_Homo403-p1	TACTGATTGCTGTTTTG	3384232
CEA_Homo180-p1	TATGTATGCGATGGTGT	1637139	CEA_Homo403-p2	TTCGATGAGCTTGACTC	3384975
CEA_Homo180-p2	TTACTTGGGAGGTTTAG	1637748	CEA_Homo404-p1	ACTCACTGAAATGCCCTAA	3391749
CEA_Homo181-p1	CTTACGCCTAAAGAAAC	1645337	CEA_Homo404-p2	AGGTTGCAGATGGTGCT	3392574
CEA_Homo181-p2	TATTAAATTCAGCCACC	1646012	CEA_Homo405-p1	AAATCAGAGCAGCAGAA	3405340
CEA_Homo182-p1	CTCGTGATGATGATGTC	1646149	CEA_Homo405-p2	TGGAAGACCAGCAGTTA	3406019
CEA_Homo182-p2	ACTATCAATACCCAAAT	1646839	CEA_Homo406-p1	GGCGAACAATTTGGATT	3406622
CEA_Homo183-p1	ATAGAAACTTCCTATGA	1646183	CEA_Homo406-p2	TCAGGCATAACGGCTAC	3407324
CEA_Homo183-p2	ATTAGTCTCCTCAACCA	1647072	CEA_Homo407-p1	GGCAAAATCGCACTTAT	3413953
CEA_Homo184-p1	CTCCTCCGTAATGTTCG	1699334	CEA_Homo407-p2	ACTTGAACGCAACCAGA	3414753
CEA_Homo184-p2	TTCAATAGCTGCAAACA	1699819	CEA_Homo408-p1	CTCTACTGCGTCTCCTA	3456158
CEA_Homo185-p1	TTGGCTTCCTATGTATT	1705386	CEA_Homo408-p2	TCTATGTTGCCACTTCT	3457102
CEA_Homo185-p2	CTCTTGCTTATGTGCTT	1706102	CEA_Homo409-p1	CTAAGAATTGGCTGAAC	3471979
CEA_Homo186-p1	TTCTACAGTACCAAGCT	1707533	CEA_Homo409-p2	AATATGGCTACAAAGGA	3472600
CEA_Homo186-p2	TAGTTCTCATACCCTTT	1708172	CEA_Homo410-p1	TGCTTACTGCTCCTAAC	3477847
CEA_Homo187-p1	CTCTGTAGATAATACCCTT	1711589	CEA_Homo410-p2	CAGTGGGAACAATAACA	3478686
CEA_Homo187-p2	AAGCTAAAACTCCCTAT	1712178	CEA_Homo411-p1	TGAGCCCTTTACCTTTA	3491761
CEA_Homo188-p1	AGACCAGGTCAGCCAGTA	1720719	CEA_Homo411-p2	CCTTGGCATAACTCTTT	3492447
CEA_Homo188-p2	CATCTTTCTCCGCCTTC	1721307	CEA_Homo412-p1	AAGCCTTAGAAGAAGAA	3513190
CEA_Homo189-p1	GAAGGGGAAGGTTGTTA	1722808	CEA_Homo412-p2	TAGCGTATGAAGTTTGT	3513709
CEA_Homo189-p2	TGAGCTTGACTTTGTGC	1723329	CEA_Homo413-p1	CATCAGCATCAGACCCTA	3534981
CEA_Homo190-p1	CCTGATGTAAAGGCACT	1747544	CEA_Homo413-p2	GCGAATACAATGTCCCT	3535816
CEA_Homo190-p2	ACATAAACTTCGCTTCC	1748151	CEA_Homo414-p1	CCCAACTTTATGAACCA	3546210
CEA_Homo191-p1	TATGGCTTCTAACACTA	1753698	CEA_Homo414-p2	TAACAGATGAGGGGCTA	3546720
CEA_Homo191-p2	TACTTCCTCCACTTCTA	1754369	CEA_Homo415-p1	AGCATACCGTAGTTGTT	3554030
CEA_Homo192-p1	TGGGCTTTCTACTAACA	1768007	CEA_Homo415-p2	TAGGCACTTTGGAGTTA	3554700
CEA_Homo192-p2	CCTTCATCGGTAAACATAA	1769182	CEA_Homo416-p1	CTTCCTCCATCTGGTTA	3566355
CEA_Homo193-p1	GTACGCTTTCGGTTCCA	1770254	CEA_Homo416-p2	TTCACATAGCGGACAAT	3566875
CEA_Homo193-p2	CACTACTCAATTCACCC	1775214	CEA_Homo417-p1	TTAATTCCACTGCACGAT	3593365
CEA_Homo194-p1	GTTAAAGAAAGCAGGTC	1774502	CEA_Homo417-p2	CAGCGGTTTGAAAGGTA	3594000
CEA_Homo194-p2	TTCCACCAGTTTCCAATAC	1779869	CEA_Homo418-p1	CATAGCCTCAGTTGATC	3595514
CEA_Homo195-p1	GAAGAGTACGCTTTCGG	1779242	CEA_Homo418-p2	GGACTACAGGGAAACAA	3596166
CEA_Homo195-p2	ATACCATTACCTTGACC	1780116	CEA_Homo419-p1	TATTCTAATCCTGCTGC	3612064
CEA_Homo196-p1	CTGTTACCAGCGTCAGG	1781949	CEA_Homo419-p2	CAGGGATACTTCCAACA	3612754

CEA_Homo196-p2	ATCACCCATTCCACCAA	1782539	CEA_Homo420-p1	CACATCCAGCCATTACG	3614087
CEA_Homo197-p1	ATTGGTGGAATGGGTGA	1782566	CEA_Homo420-p2	AACTTGGACTGCGGACA	3614933
CEA_Homo197-p2	CCGCCAAGACCTAGTAAA	1783195	CEA_Homo421-p1	ATCATAAGCCAGTTCTA	3616814
CEA_Homo198-p1	GGTGGTAATAAGGATGA	1784804	CEA_Homo421-p2	AGTAACATCAGCAGCAT	3617697
CEA_Homo198-p2	TGTATGTCCTTCCATCT	1785420	CEA_Homo422-p1	TAAACCACTCCTTACTT	3620936
CEA_Homo199-p1	AATTTCCGCCTCAATAG	1784896	CEA_Homo422-p2	GGAACATTCCTGTATAA	3621530
CEA_Homo199-p2	AAAGGCTGAGGAGGTTC	1785834	CEA_Homo423-p1	CTGGAATGTTACTATGA	3628796
CEA_Homo200-p1	GAAGGTAAGGGTGCTAT	1786805	CEA_Homo423-p2	AAACTAGAGTAAGCAGT	3629370
CEA_Homo200-p2	TTATCTGCCTGAACAAA	1787399	CEA_Homo424-p1	TTAGGCGGTATGCTTGT	3630915
CEA_Homo201-p1	AAGTTGCAGATTTGCGTAC	1788527	CEA_Homo424-p2	TTTGTTATAGGAGGTTC	3631583
CEA_Homo201-p2	TTCGGATTATGAAGTGCTG	1789074	CEA_Homo425-p1	TAGTATCGCCTAAGGGTG	3639418
CEA_Homo202-p1	AGAATGGGACATTTGAT	1789553	CEA_Homo425-p2	GCACGGGTGATAGTCAT	3640381
CEA_Homo202-p2	GGAGTTCTTATTGCCTA	1790107	CEA_Homo426-p1	AAGGCTTCTTCTTGATT	3663277
CEA_Homo203-p1	GTCCAAGAGGGAGCAGT	1791036	CEA_Homo426-p2	ATAAGTTCCTCGCTGTG	3663856
CEA_Homo203-p2	AGGGATAGATGGCGAAT	1792010	CEA_Homo427-p1	TTGCTCCCATTTCCTTA	3696117
CEA_Homo204-p1	TTGGAAAAGATAGTAAAGC	1792822	CEA_Homo427-p2	TTATGCCACGCTCAAGT	3696799
CEA_Homo204-p2	CTATCGTGGGGAAGAGT	1793868	CEA_Homo428-p1	GGGGATACCCACTTTTA	3697256
CEA_Homo205-p1	TATGATGTCTACAAAGGTT	1796084	CEA_Homo428-p2	ATGCCAGCTACATTTCT	3697780
CEA_Homo205-p2	TCCGAGGTTTTCTACTA	1796941	CEA_Homo429-p1	TTTCTTCCCAGCCAAAG	3700263
CEA_Homo206-p1	GAGGGTATGGCAGCAAA	1798930	CEA_Homo429-p2	TCCGTGCTATTCAAGGTAT	3700924
CEA_Homo206-p2	TGAGTCCGCCGTGTCTA	1799474	CEA_Homo430-p1	AAGTGCTACAGCCTCAT	3739867
CEA_Homo207-p1	TATAAAAGGTGGAGCAT	1801316	CEA_Homo430-p2	AACATCATCGTCCTCAA	3740731
CEA_Homo207-p2	CATTTGTAAACTGGGAT	1802143	CEA_Homo431-p1	TTTCAACGGCGATTTTA	3768063
CEA_Homo208-p1	CTATGAAGTGGCGAAGG	1801385	CEA_Homo431-p2	GTGGAAGCGGAACAGAG	3768632
CEA_Homo208-p2	AACTGCTCAGGACCAAC	1802306	CEA_Homo432-p1	CAACCTTCGCCTTCATC	3799627
CEA_Homo209-p1	GCACAGATAGGGATGGA	1805641	CEA_Homo432-p2	ATGGCAAGACAAGCAGATA	3800138
CEA_Homo209-p2	ACGTAAGCGAACAGATT	1806299	CEA_Homo433-p1	TTTCCAGCACCACATAC	3800287
CEA_Homo210-p1	CGGCTAAAGATACTGAA	1811751	CEA_Homo433-p2	CCTCTTTCCCTTACCAG	3800986
CEA_Homo210-p2	AATAACTATGGCTGGAC	1812513	CEA_Homo434-p1	TAATCGGTTGATCCTCA	3814666
CEA_Homo211-p1	TAAGTGAGGATAAGGGATA	1816763	CEA_Homo434-p2	GTATCCATTCCAAGCAG	3815176
CEA_Homo211-p2	CCCATTCAGAAGGCATA	1817711	CEA_Homo435-p1	ATTGGGTGCGCCTAATA	3817173
CEA_Homo212-p1	AACTGTAGCAGCGGGTGT	1821063	CEA_Homo435-p2	ATTTCGCCATTTCCTTT	3817840
CEA_Homo212-p2	GCATTCTTTGCCTGTATTT	1821559	CEA_Homo436-p1	AGCTGTATTACCCATTT	3833246
CEA_Homo213-p1	TTATTCTACAAAGGGAG	1824617	CEA_Homo436-p2	CTAAGCAGCAATATCAA	3834014
CEA_Homo213-p2	TTAACAGTGTACGCAAT	1825208	CEA_Homo437-p1	ATAAGCATACCATAAAAGG	3849680
CEA_Homo214-p1	TTATTCTACAAAGGGAG	1824654	CEA_Homo437-p2	TTCCGATAAAGAGTCGA	3850447
CEA_Homo214-p2	TTAACAGTGTACGCAAT	1825229	CEA_Homo438-p1	GCACCTTTGTAATAAGC	3867520
CEA_Homo215-p1	TGATTTAGAGTCGGAGGG	1838505	CEA_Homo438-p2	AATGGGTCACAGAGTAG	3868040
CEA_Homo215-p2	TTCGGAAAGTGGTTGAG	1839274	CEA_Homo439-p1	TTTATCCTCCATTTCAG	3897237
CEA_Homo216-p1	AAATATCCCGATGTGAG	1853573	CEA_Homo439-p2	AATAGGCACAGTAGCAG	3897930
CEA_Homo216-p2	TAGCCTCTACTCCATCTAA	1854091	CEA_Homo440-p1	AATGTTCTTACTTCGTC	3938114
CEA_Homo217-p1	ATGGGAACTTGAAATAG	1857936	CEA_Homo440-p2	ATATTAACTATGCCTCC	3938629
CEA_Homo217-p2	AACTCTGCCTTGGGAAT	1858588	CEA_Homo441-p1	CTTGTTTTGTCCCTTTT	p8959
CEA_Homo218-p1	CTGACTACAGCGGACAT	1879385	CEA_Homo441-p2	TTAATACCAGCCCTAAT	p9662

CEA_Homo218-p2	AACAGGCTCATCTAAAA	1880226	CEA_Homo442-p1	ACCTGCCATTTGACTTA	p60894
CEA_Homo219-p1	GGAGGATGTATCAAAGA	1886314	CEA_Homo442-p2	TAGGCGCAGTAGAACAC	p61471
CEA_Homo219-p2	GAAATGACTAACGGCTC	1887363	CEA_Homo443-p1	CATTTCTTGATGCTTTC	p87482
CEA_Homo220-p1	GTTATCTGCCCTTCATT	1886799	CEA_Homo443-p2	TTGCGTTAATAGTGTTAGT	p88221
CEA_Homo220-p2	AACCCAGGACCTTTATT	1887675	CEA_Homo444-p1	AGCCCAAGCTACAGAAT	p126675
CEA_Homo221-p1	TGACATTTATTTTGCATGC	1891893	CEA_Homo444-p2	TGACCCTACAACTAATCCT	p127347
CEA_Homo221-p2	AGCTGGATTTCCCACTT	1892540	CEA_Homo445-p1	TATCAAAGTTCGCAGTA	p131235
CEA_Homo222-p1	AAAAGATGCAGAACAAG	1894881	CEA_Homo445-p2	CTTAAAGGCTTCAAGTA	p131861
CEA_Homo222-p2	CTGATGGCCTAGAGTAA	1895424	CEA_Homo446-p1	GAGTGAGCGTTCTTTAC	p348460
CEA_Homo223-p1	TTTGTAGCTCCTAATGG	1896228	CEA_Homo446-p2	CACCTCCATTACATTTT	p349037
CEA_Homo223-p2	TCTGTATTGGGTTCCTG	1896767	CEA_Homo447-p1	GTTATTTCCGCTGATTT	p185531
CEA_Homo224-p1	TCTTGAAAGGCCGTGAT	1906641	CEA_Homo447-p2	TAGTACAGCTCCACTCG	p186043

* The location in genome: the number means the primer starting sites in EA 2018 genome. “p” indicates megaplasmid in EA 2018.
