# Supplementary figures and images for: Comparative genomic and transcriptomic analysis revealed genetic characteristics related to solvent formation and xylose utilization in Clostridium acetobutylicum EA 2018
Source: BMC Genomics. 2011 Feb 2;12:93. doi: 10.1186/1471-2164-12-93 (PMC3044671; doi:10.1186/1471-2164-12-93)

Additional file 9. The scheme of targeted gene disruption using group II intron.

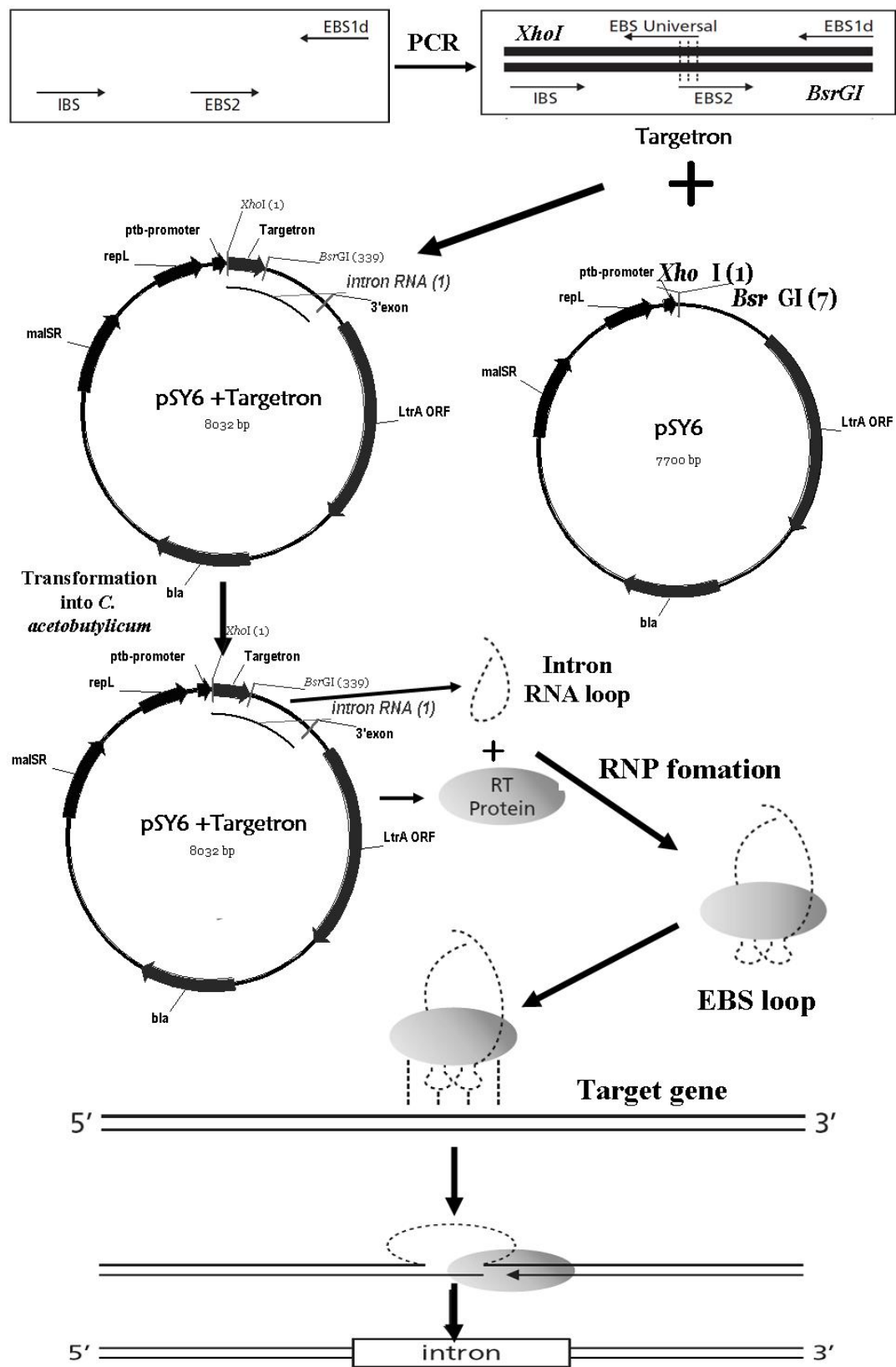

Supplement: Additional file 9 — The scheme of targeted gene disruption using group II intron. The file displays the gene disruption procedure using group II intron. [file 1471-2164-12-93-S9.PDF]
