# Supplementary material for: Comparative genomic and transcriptomic analysis revealed genetic characteristics related to solvent formation and xylose utilization in Clostridium acetobutylicum EA 2018
Source: BMC Genomics. 2011 Feb 2;12:93. doi: 10.1186/1471-2164-12-93 (PMC3044671; doi:10.1186/1471-2164-12-93)
Supplement: Additional file 10 — Targeted gene disruption and verification primers. The file lists the primers for retargeting the RNA portion of the intron for C. acetobutylicum CAC2613 gene disruption and for identifying the insertion mutant.. [file 1471-2164-12-93-S10.PDF]

Additional file 10. Targeted gene disruption and verification primers.

Primers name	Sequences
CAC2613-532-533-IBS	aaaactcgagataattatccttaagcctctctttcgtgcgcccagatagggtg
CAC2613-532-533-EBS1d	cagattgtacaaatgtggtgataacagataagtctctttccataacttacctttctttgt
CAC2613-532-533-EBS2	tgaacgcaagtttctaatttcgattaggcttcgatagaggaaagtgtct
CAC2613-ID-fw	gatggcactttacttga
CAC2613-ID-rev	cctttgatactcctcca
